# Molecular Diversity between Salivary Proteins from New World and Old World Sand Flies with Emphasis on *Bichromomyia olmeca*, the Sand Fly Vector of *Leishmania mexicana* in Mesoamerica

**DOI:** 10.1371/journal.pntd.0004771

**Published:** 2016-07-13

**Authors:** Maha Abdeladhim, Iliano V. Coutinho-Abreu, Shannon Townsend, Silvia Pasos-Pinto, Laura Sanchez, Manoochehr Rasouli, Anderson B. Guimaraes-Costa, Hamide Aslan, Ivo M. B. Francischetti, Fabiano Oliveira, Ingeborg Becker, Shaden Kamhawi, Jose M. C. Ribeiro, Ryan C. Jochim, Jesus G. Valenzuela

**Affiliations:** 1 Vector Molecular Biology Section, Laboratory of Malaria and Vector Research, National Institute of Allergy and Infectious Diseases, National Institutes of Health, Rockville, Maryland, United States of America; 2 Unidad de Investigación en Medicina Experimental, Centro de Medicina Tropical, Facultad de Medicina, Universidad Nacional Autónoma de México, México City, Mexico; 3 Faculty of Health Science, Selahaddin Eyyubi University, Diyarbakir, Turkey; 4 Vector Biology Section, Laboratory of Malaria and Vector Research, National Institute of Allergy and Infectious Diseases, National Institutes of Health, Rockville, Maryland, United States of America; New York University, UNITED STATES

## Abstract

**Background:**

Sand fly saliva has been shown to have proteins with potent biological activities, salivary proteins that can be used as biomarkers of vector exposure, and salivary proteins that are candidate vaccines against different forms of leishmaniasis. Sand fly salivary gland transcriptomic approach has contributed significantly to the identification and characterization of many of these salivary proteins from important *Leishmania* vectors; however, sand fly vectors in some regions of the world are still neglected, as *Bichromomyia olmeca* (formerly known as *Lutzomyia olmeca olmeca*), a proven vector of *Leishmania mexicana* in Mexico and Central America. Despite the importance of this vector in transmitting *Leishmania* parasite in Mesoamerica there is no information on the repertoire of *B*. *olmeca* salivary proteins and their relationship to salivary proteins from other sand fly species.

**Methods and Findings:**

A cDNA library of the salivary glands of wild-caught *B*. *olmeca* was constructed, sequenced, and analyzed. We identified transcripts encoding for novel salivary proteins from this sand fly species and performed a comparative analysis between *B*. *olmeca* salivary proteins and those from other sand fly species. With this new information we present an updated catalog of the salivary proteins specific to New World sand flies and salivary proteins common to all sand fly species. We also report in this work the anti-Factor Xa activity of Lofaxin, a salivary anticoagulant protein present in this sand fly species.

**Conclusions:**

This study provides information on the first transcriptome of a sand fly from Mesoamerica and adds information to the limited repertoire of salivary transcriptomes from the Americas. This comparative analysis also shows a fast degree of evolution in salivary proteins from New World sand flies as compared with Old World sand flies.

## Author Summary

Leishmaniasis is a neglected disease caused by a parasite transmitted to the host by the bite of an infected sand fly. Sand fly saliva contains biologically active components that allow the sand fly to take a blood meal and also the parasite to spread in the host by countering the host immune mechanisms that fights the parasite. Research on sand fly saliva has allowed us to understand the biological functions of some of these proteins, to identify salivary proteins producing an immune response in different hosts and to select potential salivary vaccine that could be used to protect the host against the parasite. However, vectors transmitting different species of *Leishmania* in diverse regions of the world are still neglected. The present work focuses on the identification of the secreted proteins from the saliva of *B*. *olmeca*, a vector of *Leishmania mexicana* in North and Central America. We catalogued these proteins with those previously identified in other sand fly species from Old and New World. We showed here how conserved or divergent are these proteins families when comparing different sand fly species. We also report the anti-Factor Xa activity of Lofaxin, a salivary anticoagulant protein identified in the saliva of this sand fly species.

## Introduction

Leishmaniasis is a tropical neglected disease caused by *Leishmania* parasites transmitted by phlebotomine sand flies. There have been significant advances related to the biology and immune responses to the *Leishmania* parasite but much less studies are devoted to its vector, the sand fly, particularly from areas where the disease is overlooked.

Female sand flies may inoculate the protozoan parasites together with saliva into the host’s skin while acquiring a blood meal. The injected saliva comprises anti-hemostatic components that facilitate the feeding process, as well as bioactive molecules that modulate the host immune system [1,2]. Sand fly saliva was previously shown to enhance *Leishmania* infection in naïve mice, while pre-exposure to sand fly saliva or uninfected sand fly bites confers protection against Leishmanisis in rodents and non-human primate models [3–5]. Importantly, transcriptomic studies of sand fly salivary glands from different regions of the world have significantly contributed to the identification of protective salivary proteins for different animal models of cutaneous and visceral leishmaniasis [1]. Immunogenic sand fly salivary proteins are becoming practical biomarkers of phlebotomine exposure and are becoming an attractive tool for epidemiological and vector surveillance studies [6–9]. There is a significant number of salivary gland transcriptomes from sand flies belonging to five out of twelve subgenera of the genus *Phlebotomus*, including the subgenera Phlebotomus (*Phlebotomus papatasi* and *P*. *duboscqi* [4,10,11]), Larroussius (*P*. *ariasi*, *P*. *perniciosus*, *P*. *orientalis*, and *P*. *tobbi* [12–16]), Euphlebotomus (*Phlebotomus argentipes* [15]), Adlerius (*Phlebotomus arabicus*; [17]), *and* Paraphlebotomus (*Phlebotomus sergenti*; [12]). On the other hand, the number of salivary gland transcriptomes of New World sand flies is limited. Only three transcriptomes are available, two belonging to species of the genus Lutzomyia including the subgenera Lutzomyia (*Lutzomyia longipalpis* [18]) and Helcocyrtomyia (*Lutzomyia ayacuchensis* [19]), and one belonging to the genus Nyssomyia (*Nyssomyia intermedia* [20]). All three species are present in South America.

In order to obtain further insight into the salivary components of New World sand fly species, particularly from uncharted geographical areas, the salivary gland transcriptome of *B*. *olmeca* (formerly known as *Lutzomyia olmeca olmeca*) was analyzed. This sand fly species is present in North and Central America (Mesoamerica region) and is a proven vector of *Leishmania mexicana*, the causative agent of cutaneous leishmaniasis in this region [21]. The comparative analysis of the salivary gland protein families of New World and Old World sand flies were also performed to describe proteins unique to New World sand flies and also to shed light on the evolutionary processes that led to the molecular diversification of such proteins families in vector sand flies.

## Methods

### Sand fly salivary gland dissection

*Bichromomyia olmeca* sand flies were collected from 15 to 18 of February, 2011, in two localities of the Cunduacan municipality in Tabasco, Mexico: Rancho Culico and Dora´s Hacienda (S1 Fig). All the specimens were captured between 18:00 and 22:00 using Shannon traps. The sand flies were identified according to Young and Duncan (1994). The morphology of their spermatheca, the ratio of the pulpus length to the antenna, the thorax color and the measurements of the wing veins were considered. The salivary glands were dissected in saline buffer under sterile conditions and stored in RNA later (Qiagen, Santa Clara, California, USA) at 4°C.

### Construction of the salivary gland cDNA library

Salivary glands mRNA was isolated from 50 pairs of salivary glands of wild-caught sand flies using Micro-FastTrackTM mRNA isolation kit (Invitrogen, San Diego, California, USA). PCR-based cDNA library was performed following the manufacturer’s instructions for the SMART cDNA library construction kit (BD Clontech, Palo Alto, California, USA) with some modifications as previously described [18]. The cDNA library was fractionated into three sets of cDNAs containing large, medium, and small fragments and visualized on an agarose gel. Gigapack III gold packaging extract (Stratagene, La Jolla, California, USA) was used for packaging phage particles. The libraries (large, medium, and small) were plated by infecting log phase XL-1 blue *Escherichia coli* (Clontech). Many plaques from each plate were selected, and a PCR with selected vector-specific primers flanking the inserted cDNA was performed [10]. The presence of recombinants was checked by visualization of PCR products on 1.1% agarose gel with Syber safe (Roche Diagnostics, Mannheim, Germany).

### Sequencing of selected cDNA clones

Plaques were randomly selected from the plated libraries and transferred to a 96 well polypropylene plate containing 30 μl of water per well. The PCR reaction amplified randomly selected cDNAs using FastStart PCR Master Mix (Roche), 3 μl of the phage sample as a template, and the specific vector primers PT2F1 (59-AAG TAC TCT AGC AAT TGT GAG C-3’), which is positioned upstream from the cDNA of interest (5’ end), and PT2R1 (5’- CTC TTC GCT ATT ACG CCA GCT-3’), which is positioned downstream from the cDNA of interest (3’ end). Amplification conditions were as follows: 1 hold of 75°C for 3 min, 1 hold of 94°C for 4 min, and 30 cycles of 94°C for 1 min, 49°C for 1 min, and 72°C for 2 min. The final elongation step lasted for 7 min at 72°C. Reaction products were cleaned using ExcelaPure 96-well UF PCR purification plates (EdgeBiosystems, Gaithersburg, Maryland, USA) and used as templates for cycle-sequencing reaction. Cycle sequencing reactions were performed at the Research Technology Branch at the Rocky Mountain Labs, NIAID.

### Bioinformatics

Bioinformatic analysis was performed as previously described [17]. Raw sequence files were analyzed using a customized program [22] DNA sequences with Phred quality scores lower than 20, including primer and vector sequences, were discarded. Sequences were then grouped into clusters using a customized program based on identity (95% identity) and aligned into contiguous sequences (contigs) using the CAP3 program. Contigs were then analyzed by blastx, blastn, or rpsblast programs and compared to the non-redundant (NR) protein database of the National Center for Biotechnology Information (NCBI), the gene ontology (GO) FASTA subset, and the conserved domains database (CDD) of NCBI, which contains EuKaryotic Orthologous Groups (KOG), protein families (Pfam), and simple modular architecture research tool (SMART) databases.

The three potential translations of each dataset were submitted to the SignalP server to detect signal peptides. All the analyzed sequences were combined in an Excel spreadsheet and manually verified and annotated.

### Sequence alignment

Multiple sequence alignment of putative peptides was accomplished using Clustal Omega. Alignment outputs were converted to rich text files for figure annotation.

### DNA polymorphism, protein divergence, and phylogenetic analysis

Gene conversion and natural selection analyses were performed with the software DnaSP [23]. The parameter ω refers to the rate of non-synonymous nucleotide polymorphisms (Ka) over the synonymous rate of nucleotide polymorphisms (Ks) [24]. Slide window analyses of ω along the nucleotide sequences encoding such proteins were also obtained. For these analyses, sequences encoding the signal peptide and stop codon were excluded. The parameter ψ was also calculated, indicating the average number of informative nucleotide sites per site concerning the sites under gene conversion [25].

The diversity of the protein family sequences was obtained by subtracting the protein identities from 1 (diversity = 1 –identity).

The evolutionary histories of salivary protein families were inferred by using the Maximum Likelihood method and conducted in MEGA6 [26]. The amino acid substitution model was selected based on the best fit provided by the Model Selection tool built in the MEGA 6 software. The bootstrap consensus trees inferred from 1000 replicates [27] were taken to represent the evolutionary history of the taxa analyzed [27]. Branches corresponding to partitions reproduced in less than 50% bootstrap replicates are collapsed. Initial tree(s) for the heuristic search were obtained by applying the Neighbor-Joining method to a matrix of pairwise distances estimated using a JTT model. Only the sequences for the mature proteins were included for the phylogenetic analyses.

### Cloning and protein expression

DNA of the targeted molecules was amplified by polymerase chain reaction (PCR) using a forward primer deduced from the amino-terminus sequence (starting after the signal peptide) and a reverse primer encoding a hexa-histidine motif.

The PCR conditions were: one hold for 5 min at 94°C, two cycles of 30 s at 94°C, 1 min at 46°C, 1 min at 72°C and 23 cycles of 30s at 94°C, 1 min at 52°C, 1 min at 72°C and one hold of 7 min at 72°C. The PCR product was cloned into the VR2001- TOPO vector as previously described [14] and then sequenced. The VR-2010 plasmid coding for the target proteins containing a 6 histidine tag was sent to the Protein Expression Laboratory at NCI-Frederick (Frederick, Maryland) for expression in HEK-293F cells. The supernatant was collected after 72 hours and concentrated from 1 L to 300 ml using a Stirred Ultrafiltration Cell unit (Millipore) with a 10 kDa ultrafiltration membrane (Millipore). The volume was returned to 1 L by the addition of 500 mM NaCL and 10 mM Tris, pH 8.0. The protein was purified by an HPLC system (Biorad, NGC chromatography system) using two 5 ml HiTrap Chelating HP columns (GE Healthcare) in tandem and charged with 0.1 M NiSO4. The protein was detected at 280 nm and eluted by an imidazole gradient from 50 mM to 500 mM. Eluted proteins were collected every minute in a 96-well microtiter plate using a BioFrac fraction collector (Biorad). Fractions corresponding to peak(s) were selected and run on a NuPage Bis-Tris 4–12% Gel (Novex) with MES running buffer under reducing conditions as per manufacturer’s instructions. Afterwards, the gel was stained with Coomassie Blue (0.025%) to visualize proteins. Appropriate fractions as determined by molecular weight compared to a standard in the gel were pooled and concentrated to 1 ml using a 10 kDa Amicon Ultra Centrifugal Filter (Millipore). The protein sample was then injected into a g2000sw molecular sieving column (Tosoh Biosciences) with a 1 ml loop connected to the HPLC (DIONEX) with a phosphate buffer (PBS) pH 7.2 as the buffer for further purification. The protein was detected at 280 nm and the fractions were collected as described above. Appropriate fractions were determined as described above and pooled. Concentration was measured by using a NanoDrop ND-1000 spectrophometer at 280 nm and calculated using the extinction coefficient of the protein.

### Surface Plasmon Resonance (SPR)

All SPR experiments were carried out in a T100 instrument (Biacore Inc., Uppsala, Sweden) following the manufacturer's instructions. For immobilization using an amine coupling kit (Biacore), CM5 chips were activated with 1-ethyl-3-(dimethylaminopropyl) carbodiimide, and N-hydroxysuccinimide before injection of FXa (30 μg/mL) in acetate buffer, pH 5. Remaining activated groups were blocked with 1 M ethanolamine, pH 8.5, resulting in a final immobilization of 1455.7 RU. Kinetic experiments were carried out by injecting Lofaxin for a contact time of 180 seconds at a flow rate of 30 μL/minute at 25°C. For all runs, HBS-P buffer was used (10 mM HEPES, 150 mM NaCl, 0.005% surfactant P20, pH 7.4). FXa-Lofaxin complex dissociation was monitored for 600 seconds, and the sensor surface was regenerated by a pulse of 30 seconds of 10 mM Glycine pH 3 at 30 μL/minute. Blank flow cells were used to subtract the buffer effect on sensorgrams. After subtraction of the contribution of bulk refractive index and nonspecific interactions with the CM5 chip surface, the individual association (*ka*) and dissociation (*kd*) rate constants were obtained by global fitting of data using the 1:1 model (Langmuir) interaction model using BIAevaluation™ (Biacore, Inc.). Values were then used to calculate the equilibrium constant (*KD*). The values of average squared residual obtained were not significantly improved by fitting data to models that assumed other interactions. Conditions were chosen so that the contribution of mass transport to the observed values of *KD* was negligible. Also, models in the T100 evaluation software fit for mass transfer coefficient to mathematically extrapolate the true *ka* and *kd*.

### Recalcification time assay

A pool of human citrated plasma (NIH blood bank) was added (30 ul) in a 96 well plate. An equal volume of HBSS buffer (30 ul) was added with or without rLofaxin protein (1ng, 5ng, 10ng and 20ng/reaction). The plate was incubated at 37°C for 10 min. To initiate the blood coagulation cascade, 30 μl of 10 mM calcium chloride was added to the plasma. The reaction was followed at 405 nm using a Versamax microplate reader (Molecular devices) at 37°C every 5 seconds until a clot was observed. The onset time was the time it took for the clot to form at OD (405 nm).

### Hemolytic assay for complement activation

To evaluate the effect of the salivary recombinant proteins LolSALOa and LolSALOd on complement activation, we performed a classical pathway-mediated lysis assay by using antibody-coated sheep erythrocytes (CompTech). Normal human serum (CompTech; final concentration of 2.5%) was incubated with or without rLolSALOa or rLolSALOb (final concentration 0.6 μM) and 5 x 10^6^ erythrocytes (final volume of 62.5 μL) in GVB^++^ solution (0.1% gelatin, 5 mM Veronal, 145 mM NaCl, 0.025% NaN_3_, 0.15 mM CaCl_2_ and 0.5 mM MgCl_2_, pH 7.4 –CompTech^R^). As a control, PBS was added instead of recombinant proteins. After 30 min at 37°C, 100 μL of cold phosphate-buffered saline (PBS) was added and samples were centrifuged. The supernatant (100 μL) was transferred to a 96-well flat bottom plate and the optical density was determined at 415 nm. Incubation of erythrocytes with human serum in the absence of recombinant proteins was considered as 100% of hemolysis. To compare the extension of complement inhibition, we also performed this assay with recombinant *Lutzomyia longipalpis* SALO (0.6 μM), a recently reported classical pathway complement inhibitor from *Lutzomyia longipalpis* [28].

## Results and Discussion

The primary objective of this work was to identify the repertoire of salivary proteins of *B*. *olmeca*, a New World sand fly from Mesoamerica, and to compare it to those of other New and Old World sand flies. A *B*. *olmeca* cDNA library was constructed from salivary glands of wild-caught female sand flies collected on Tabasco State, Mexico. Transcripts from the salivary gland cDNA library were isolated, sequenced, and analyzed using an in-house bioinformatics pipeline as previously described [28,29]. Assembly of 1765 high-quality transcripts led to the identification of 607 contigs, including 418 singletons. Annotation of these contigs indicated that 40% of the transcripts are coding for putative secreted proteins, 43% are coding for housekeeping proteins, and 17% for other unknown products. The latter may derive from incomplete mRNAs in the library. **Table 1**shows the analyzed contigs in a descending order (from the largest to the smallest number of sequences per contig) and the resulting similarities identified using the ‘‘basic local alignment search tool” (BLAST) in the non-redundant (NR) or Transcriptome Shotgun Assembly (TSA-NR) databases. The most abundant transcripts on this table match those of secreted proteins previously identified in salivary gland transcriptomes from various sand fly species, suggesting that these proteins are targeted for secretion and may represent the inoculated proteins into the host skin when a sand fly attempts to get a blood meal.

**Table 1:** Analyzed contigs in a descending order and the resulting similarities.

Contig Number	Number of sequences in contig	Best match to NR database	E-values of NR match	Comments
54	128	hypothetical protein	2.9	Toxin-like protein
73	106	9.6 KDa salivary protein	1E-012	SALO-like-protein
109	71	hypothetical protein	14	Hypotetical protein
131	58	9.6 KDa salivary protein	2E-012	SALO-like protein
70	48	9.6 KDa salivary protein	4E-013	SALO-like protein
76	26	16.3 kDa salivary protein	4E-030	C-type-lectin-like protein
83	24	16.3 kDa salivary protein	3E-028	C-type-lectin-like protein
8	23	10 kDa salivary protein SP13	0.77	RGD-like protein
123	18	initiation factor 2 subunit	2.0	Toxin-like protein
51	17	hypothetical protein	0.26	Toxin-like protein
130	16	9.6 KDa salivary protein	5E-012	SALO-like protein
21	15	SL1 protein	7E-042	Small OBP-like protein
80	15	16.3 kDa salivary protein	2E-029	C-type-lectin-like protein
58	13	hypothetical protein	2.9	Toxin-like protein
116	12	hypothetical protein BRAFLDRAFT_275402	1.3	Toxin-like protein
121	12	hypothetical protein BRAFLDRAFT_275402	8.1	Toxin-like protein
140	12	16.4 kDa salivary protein	6E-009	C-type-lectin-like protein
160	12	43.2 kDa salivary protein	1E-166	Yellow-like protein
172	12	hypothetical protein	5E-008	RGD-like protein
211	12	unnamed protein product	4.3	ML-domain protein
72	12	9.6 KDa salivary protein	3E-013	SALO-like protein
143	11	16.4 kDa salivary protein	3E-010	C-type-lectin-like protein
27	11	SL1 protein	2E-041	Small OBP-like protein
55	11	hypothetical protein	2.9	Toxin-like protein
9	11	10 kDa salivary protein SP13	0.26	RGD-like protein
167	10	14.2 kDa salivary protein	5E-005	14.2-like protein
178	9	antigen 5-related protein	1E-126	Antigen-5 related protein
5	9	10 kDa salivary protein SP13	0.77	RGD-like protein
84	9	16.3 kDa salivary protein	4E-028	C-type-lectin-like protein
91	9	16.3 kDa salivary protein	1E-028	C-type-lectin-like protein
120	8	hypothetical protein BRAFLDRAFT_275402	8.0	Toxin-like protein
15	8	10 kDa salivary protein SP13	0.45	RGD-like protein
16	8	SL1 protein	0.046	Small OBP-like protein
181	8	29.2 kDa salivary protein	2E-057	Silk-related protein
182	8	29.2 kDa salivary protein	1E-057	Silk-related protein
217	8	voltage-dependent calcium channel type D subunit alpha-1-like isoform 2	9.0	Toxin-like protein
71	8	9.6 KDa salivary protein	4E-013	SALO-like protein
78	8	16.3 kDa salivary protein	4E-030	C-type-lectin-like protein
87	8	anticoagulant	1E-029	Lufaxin-like protein
90	8	anticoagulant	2E-029	Lufaxin-like protein
1	7	10 kDa salivary protein SP13	1.00	RGD-like protein
118	7	conserved Plasmodium membrane protein, unknown function	2.5	Toxin-like protein
18	7	SL1 protein	4E-042	Small OBP-like protein
241	7	hypothetical protein	5.9	Toxin-like protein
246	7	9.6 KDa salivary protein	2E-013	SALO-like protein
6	7	10 kDa salivary protein SP13	2.9	RGD-like protein
119	6	DNA-directed RNA polymerase subunit beta''; AltName: Full=PEP; AltName:	0.73	Toxin-like protein
154	6	conserved hypothetical protein	4.4	ML-domain protein
166	6	14.2 kDa salivary protein	6E-005	14.2-like protein
179	6	antigen 5-related protein	1E-126	Antigen-5 related protein
220	6	Serpentine Receptor, class X family member (srx-22)	4.4	Toxin-like protein
11	5	10 kDa salivary protein SP13	0.59	RGD-like protein
141	5	16.4 kDa salivary protein	5E-009	C-type-lectin-like protein
177	5	antigen 5-related protein	1E-126	Antigen-5 related protein
197	5	10 kDa salivary protein	0.77	RGD-like protein
234	5	anticoagulant	5E-029	Lufaxin-like protein
248	5	hypothetical protein LAU_0420	4.0	ML-domain protein
297	5	oxytocin/vasopressin-like peptide	3.8	Toxin-like protein
312	5	43.2 kDa salivary protein	1E-104	Yellow-like protein
82	5	16.3 kDa salivary protein	4E-030	C-type-lectin-like protein
85	5	16.3 kDa salivary protein	1E-028	C-type-lectin-like protein
89	5	anticoagulant	1E-029	Lufaxin-like protein
13	4	10 kDa salivary protein SP13	0.75	RGD-like protein
138	4	16.4 kDa salivary protein	6E-009	C-type-lectin-like protein
17	4	10 kDa salivary protein SP13	0.77	RGD-like protein
2	4	10 kDa salivary protein SP13	0.77	RGD-like protein
252	4	flagelliform silk protein	0.002	Toxin-like protein
26	4	SL1 protein	5E-042	Small OBP-like protein
304	4	hypothetical protein PSYPI_16920	1.9	Toxin-like protein
31	4	trans-sialidase, putative	2.9	
343	4	similar to MPA2 allergen	5E-006	ML-domain protein
4	4	10 kDa salivary protein SP13	0.99	RGD-like protein
52	4	hypothetical protein	0.44	Toxin-like protein
88	4	anticoagulant	8E-030	Lufaxin-like protein

We further analyzed full-length transcripts coding for secreted proteins grouping them by families and describing their predicted molecular weight (MW), isoelectric point (pI), the organism or sand fly with the best match, the accession number of the protein of best match and the percentage of identity (**Tables 2–16**). **Table 16**includes truncated proteins that matched previously reported secreted proteins in other sand flies. The presence of truncated proteins is most likely due to the limitations of the sequencing approach used in the current work. New technologies such as RNAseq should improve the number of full-length sequences of large proteins from salivary gland transcriptomes.

**Table 2:** Putative secreted SALO salivary protein family from *Bichromomyia olmeca*.

				Best match to NR database		
Sequence name	NCBI accession number	MW	pI	Species of best match	Accession number of best match	% Identity
SALO protein family						
LolSALOa	KX011350	8.393	5.65	*Lutzomyia longipalpis*	AAR99724	35%
LolSALOb	KX011351	8.077	5.84	*Lutzomyia longipalpis*	AAR99724	36%
LolSALOc	KX011352	8.742	4.74	*Nyssomyia intermedia*	AFP99234	35%
LolSALOd	KX011353	9.865	5.35	*Nyssomyia intermedia*	AFP99253	60%
LolSALOe	KX011354	11.180	9.63	*Nyssomyia intermedia*	AFP99249	56%
LolSALOf	KX011355	8.875	4.88	*Lutzomyia longipalpis*	AAR99724	36%

**Table 3:** Putative secreted RGD salivary protein family from *Bichromomyia olmeca*.

				Best match to NR database		
Sequencename	NCBI accession number	MW	pI	Species of best match	Accession number of best match	% Identity
RGD protein family						
LolRGD	KX011356	4.775	3.62	*Nyssomyia intermedia*	AFP99242	52%
LolnoRGD	KX011357	4.706	3.38	*Nyssomyia intermedia*	AFP99242	43%

**Table 4:** Putative secreted 5kDa salivary protein family from *Bichromomyia olmeca*.

				Best match to NR database		
Sequencename	NCBI accession number	MW	pI	Species of best match	Accession number of best match	%**Identity**
5kDa protein family						
Lol5	KX011358	5.079	3.83	*Nyssomyia intermedia*	AFP99240	66%

**Table 5:** Putative secreted C-type lectin salivary protein family from *Bichromomyia olmeca*.

				Best match to NR database		
Sequence name	NCBI accession number	MW	pI	Species of best match	Accession number of best match	% Identity
C-Type lectin protein family						
LolCTLa	KX011359	17.127	8.38	*Nyssomyia intermedia*	AFP99244	44%
LolCTLb	KX011360	17.167	8.38	*Nyssomyia intermedia*	AFP99244	46%
LolCTLc	KX011361	14.325	9.79	*Corethrella appendiculata*	JAB55018	58%
LolCTLd	KX011362	16.153	8.8	*Nyssomyia intermedia*	AFP99271	59%
LolCTLe	KX011363	17.445	9.13	*Nyssomyia intermedia*	AFP99236	70%

**Table 6:** Putative secreted 14.2kDa salivary protein family from *Bichromomyia olmeca*.

				Best match to NR database		
Sequence name	NCBI accession number	MW	pI	Species of best match	Accession number of best match	% Identity
14.2kDa protein family						
Lol14.2a	KX011364	11.684	5.29	*Nyssomyia intermedia*	AFP99254	61%
Lol14.2b	KX011365	10.323	4.72	*Lutzomyia longipalpis*	AAS16907	43%
Lol14.2c	KX011366	10.085	4.40	*Lutzomyia longipalpis*	AAS16907	41%

**Table 7:** Putative secreted ML domain salivary protein family from *Bichromomyia olmeca*.

				Best match to NR database		
Sequence name	NCBI accession number	MW	pI	Species of best match	Accession number of best match	% Identity
ML domain protein family						
LolMLa	KX011367	17.698	8.42	*Corethrella appendiculata*	JAB54888	50%
LolMLb	KX011368	15.448	8.75	*Nyssomyia intermedia*	AFP99248	78%
LolMLc	KX011369	17.209	8.88	*Nyssomyia intermedia*	AFP99264	42%
LolMLd	KX011370	14.617	9.86	*Nyssomyia intermedia*	AFP99241	66%

**Table 8:** Putative secreted Toxin-like salivary protein family from *Bichromomyia olmeca*.

				Best match to NR database		
Sequence name	NCBI accession number	MW	pI	Species of best match	Accession number of best match	% Identity
Toxin-like family of peptides						
LolToxA	KX011371	6.326	5.99	*Chilobrachys guangxiensis*	B1P1E0	57%
LolToxB	KX011372	6.440	6.77	*Pelinobius muticus*	D5J6X1	58%
LolToxC	KX011373	5.144	4.32	*Grammostola rosea*	BAN13505	45%
LolToxD	KX011374	5.642	4.93	*Nyssomyia intermedia*	AFP99257	64%
LolToxE	KX011375	5.329	4.18	*Nyssomyia intermedia*	AFP99263	52%
LolToxF	KX011376	5.649	7.55	*Nyssomyia intermedia*	AFP99272	73%
LolToxG	KX011377	6.059	7.57	*Nyssomyia intermedia*	AFP99252	48%
LolToxH	KX011378	6.108	6.65	*Nyssomyia intermedia*	AFP99252	55%
LolToxI	KX011379	5.621	8.26	*Nyssomyia intermedia*	AFP99252	59%
LolToxJ	KX011380	6.630	8.85	*Nyssomyia intermedia*	AFP99252	68%
LolToxK	KX011381	6.991	7.61	*Nyssomyia intermedia*	AFP99269	66%

**Table 9:** Putative secreted Small Odorant Binding salivary protein family from *Bichromomyia olmeca*.

				Best match to NR database		
Sequence name	NCBI accession number	MW	pI	Species of best match	Accession number of best match	% Identity
Small Odorant Binding protein (OBP)						
LolSOBPa	KX011382	14.061	9.42	*Nyssomyia intermedia*	AFP99232	76%
LolSOBPb	KX011383	13.956	9.13	*Nyssomyia intermedia*	AFP99232	60%
LolSOBPc	KX011384	14	9.61	*Nyssomyia intermedia*	AFP99266	67%

**Table 10:** Putative secreted Large Odorant Binding salivary protein family from *Bichromomyia olmeca*.

				Best match to NR database		
Sequence name	NCBI accession number	MW	pI	Species of best match	Accession number of best match	% Identity
Large Odorant Binding protein (OBP) D7 protein family						
LolD7	KX011385	25.938	8.41	*Lutzomyia longipalpis*	AAL16051	59%

**Table 11:** Putative secreted Antigen-5 salivary protein family from *Bichromomyia olmeca*.

				Best match to NR database		
Sequence name	NCBI accession number	MW	pI	Species of best match	Accession number of best match	% Identity
Antigen-5 protein family						
LolAg-5	KX011386	28.463	9.23	*Nyssomyia intermedia*	AFP99231	88%

**Table 12:** Putative secreted Silk-related salivary protein family from *Bichromomyia olmeca*.

				Best match to NR database		
Sequence name	NCBI accession number	MW	pI	Species of best match	Accession number of best match	% Identity
Silk-related protein family						
Lolsilk	KX011387	21.893	10.00	*Nyssomyia intermedia*	AFP99238	61%

**Table 13:** Putative secreted Yellow salivary protein family from *Bichromomyia olmeca*.

				Best match to NR database		
Sequence name	NCBI accession number	MW	pI	Species of best match	Accession number of best match	% Identity
Yellow protein family						
LolYLWa	KX011388	44.47	5.69	*Lutzomyia ayacuchensis*	BAM69110	63%
LolYLWb	KX011389	43.018	9.40	*Lutzomyia ayacuchensis*	BAM69111	79%
LolYLWc	KX011390	44.46	9.18	*Nyssomyia intermedia*	AFP99235	79%

**Table 14:** Putative secreted salivary Apyrase from *Bichromomyia olmeca*.

				Best match to NR database		
Sequence name	NCBI accession number	MW	pI	Species of best match	Accession number of best match	% Identity
Apyrase						
LolApy	KX011391	35.7	9.3	*Nyssomyia intermedia*	AFP99246	72%

**Table 15:** Putative secreted Lufaxin-like protein family from *Bichromomyia olmeca*.

				Best match to NR database			
Sequence name	NCBI accession number	MW	pI	Species of best match	Accession number of best match		% Identity
Lufaxin-like protein family							
Lofaxin	KX011392	32.741	8.92	*Lutzomyia ayacuchensis*	BAM69204	62%	

**Table 16:** Putative secreted truncated salivary proteins from *Bichromomyia olmeca*.

				Best match to NR database		
Sequence name	NCBI accession number	MW	pI	Species of best match	Accession number of best match	% Identity
Truncated proteins						
**Hyaluronidase**						
LolHyaz	KX011393	35.278	8.7	*Lutzomyia longipalpis*	AAD32195	70%
**Endonuclease**						
LolEndo	KX011394	32.786	9.39	*Nyssomyia intermedia*	AFP99255	80%
**Adenosine Deaminase**						
LolADA	KX011395	24.423	6.45	*Lutzomyia longipalpis*	AAF78901	53%
**Lol56.6**						
Lol56.6	KX011396	33.364	4.33	*Lutzomyia longipalpis*	AAS16908	25%
**Lol71**						
Lol71	KX011397	33.874	5.10	*Lutzomyia longipalpis*	AAS16911	91%
**Lol38.8**						
Lol38.8	KX011398	6.872	8.65	*Phlebotomus tobbi*	ADJ54098	65%

Following is an in depth description of these proteins families.

### New World-specific salivary protein families

Based on the transcripts identified in *B*. *olmeca* salivary gland cDNA library and the homologues identified in salivary gland libraries from other New World sand flies, we have cataloged seven protein families specific to New World sand flies. All the proteins we have identified to be specific to New World sand fly species are catalogued in **Table 17**.

**Table 17:** Salivary proteins shared by New and Old world sand flies.

Family of proteins	*B*. *olmeca*	*Lu*. *longipalpis*	*Lu*. *ayacuyensis*	*N*. *intermedia*	*Phlebotomus*
Proteins specific to *New World* sand flies					
**Toxins family**	**☑**	**-**	**-**	**☑**	**-**
**RGD-containing**	**☑**	**☑**	**☑**	**☑**	**-**
**C-type lectin**	**☑**	**☑**	**☑**	**☑**	**-**
**Maxadilan peptide**	**-**	**☑**	**-**	**☑**	**-**
**14kDa**	**☑**	**☑**	**-**	**☑**	**-**
**ML domain peptide**	**☑**	**-**	**-**	**☑**	**-**
**5’ Nucleotidase**	**-**	**☑**	**-**	**-**	**-**
**10 kDa family**	**☑**	**☑**	**-**	**☑**	**-**
**11.5 kDa protein**	**☑**	**☑**	**☑**	**-**	**-**
**71 kDa proteins**	**☑**	**☑**	**-**	**-**	**-**
**Molecules or Proteins specific to *Old World* sand flies**					
**Adenosine**	**-**	**-**	**-**	**-**	☑
**Glutathione 5 transferase**	**-**	**-**	**-**	**-**	☑
**Pyrophosphatase**	**-**	**-**	**-**	**-**	☑
**Phospholipase A2**	**-**	**-**	**-**	**-**	☑
**12kDa protein of unknown function**	**-**	**-**	**-**	**-**	☑
**3kDa protein of unknown function**	**-**	**-**	**-**	**-**	☑
**27kDa protein of unknown function**	**-**	**-**	**-**	**-**	☑
**Proteins common to *Lutzomyia* and *Phlebotomus* sand flies**					
**Small Odorant Binding Proteins**	**☑**	**☑**	**☑**	**☑**	☑
**Yellow proteins**	**☑**	**☑**	**☑**	**☑**	☑
**Antigen 5 related proteins**	**☑**	**☑**	**☑**	**☑**	☑
**Lufaxin**	**☑**	**☑**	**☑**	**☑**	☑
**Large OBP/D7- related proteins**	**☑**	**☑**	**☑**		☑
**Apyrase**	**☑**	**☑**	**☑**	**☑**	☑
**Endonuclease**	**☑**	**☑**	**☑**	**☑**	☑
**hyaluronidase**	**☑**	**☑**	**-**	**☑**	☑
**Silk related/collagen binding proteins**	**☑**	**☑**	**☑**	**-**	☑
**Adenosine deaminase**	**☑**	**☑**	**-**	**-**	☑
**56.6kDa proteins**	**☑**	**☑**	**-**	**-**	☑
**38.8kDa proteins**	**☑**	**-**	**-**	**-**	☑

(☑) Protein or function described

(-) protein or function not described

#### SALO protein family

**Fig 1:**
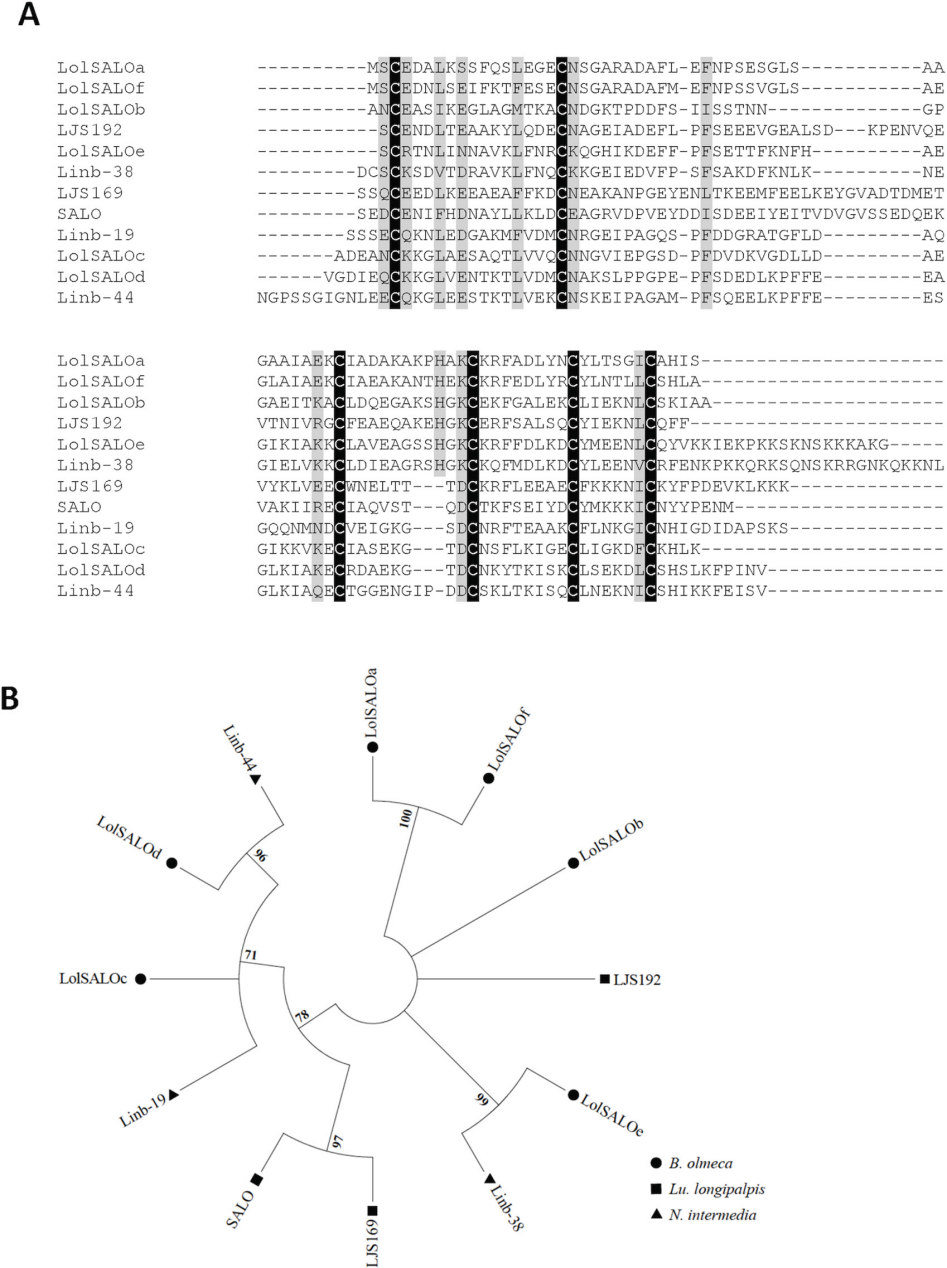
Multiple sequence alignment and molecular phylogenetic analysis of the sand fly SALO protein family. (**A**) Multiple sequence alignment. Six different SALO-like proteins (LolSALOa-f) identified from the *B*. *olmeca* salivary gland cDNA library were aligned with similar proteins described in New World sand flies *Lu*. *longipalpis* (LJS, SALO) and *N*. *intermedia* (Linb-). Black background shading represents identical amino acids. Grey background shading represents similar amino acids. (**B**) The phylogenetic relationship among the sand fly SALO proteins is displayed. Whereas a large branch encompasses the more closely related homologs, other more divergent paralogs are also observed for all the three sand fly species. The evolutionary history was inferred based on the Whelan And Goldman model [62]. Sand fly species are indicated by the different symbols in the legend on the right.

This family belongs to a novel family of anti-complement proteins recently described in the saliva of *Lu*. *longipalpis* [28]. SALO, a salivary protein of 11 kDa from *Lu*. *longipalpis*, was characterized as a specific inhibitor of the classical pathway of complement [28]. Two other members of this family of proteins in *Lu*. *longipalpis*, LJS169 and LJS192, did not show anti-complement activity [28]. SALO, formerly known as LJM19, was also characterized as a vaccine candidate against *Leishmania infantum* [30] and *L*. *braziliensis* [31] in hamsters. Six proteins from this family were identified in this *B*. *olmeca* cDNA library and were named LolSALOa, LolSALOb, LolSALOc, LolSALOd, LolSALOe, and LolSALOf (**Table 2**). These proteins display divergent amino acid sequences, yet contain six stereotypical cysteines (**S2 Fig)**, following the CX_14_CX_26-41_CX_8-11_CX_8_CX_6_C amino acid consensus signature. Importantly, this family of proteins is present in *Lu*. *longipalpis* and *N*. *intermedia* (**Fig 1A**). The phylogenetic tree depicted a single clade of SALO-like orthologs, encompassing *Lu*. *longipalpis* (SALO and LJS169), *N*. *intermedia* (Linb-19 and Linb-44), and *B*. *olmeca* (LolSALOc and LolSALOd) proteins **(Fig 1B**). The presence of multiple species-specific gene duplications, such as SALO and LJS169 (*Lu*. *longipalpis*) and LolSALOa and LolSALOf (*B*. *olmeca*), or taxa specific gene duplications (*B*. *olmeca* LolSALOc and LolSALOd and *N*. *intermedia* Linb-19 and Linb-44), points to events of gene expansion after speciation of the common ancestor between taxa (**Fig 1B)**. We evaluated the effect of two salivary recombinant proteins LolSALOa and LolSALOd on complement activation to determine if these two proteins have the same activity of SALO from *Lu*. *longipalpis*. We did not observe anti-complement activity from either LolSALOa or LolSALOd (**S3 Fig**), suggesting these two proteins do not have the same properties as SALO from *Lu*. *longipalpis*. It is possible that one of the other 4 members of the LolSALO family may have anti-complement activity and this needs to be further investigated.

The SALO family of salivary proteins seems to be evolving at a relatively rapid rate, as there are very limited amino acid similarities between sequences besides the conserved cysteine residues (**S2 Fig**). Due to the vaccine potential of SALO [30,31] identified from *Lu*. *longipalpis*, the immunogenicity of the above-mentioned orthologs in other vector species deserves further investigation.

#### RGD-containing peptide family

**Fig 2:**
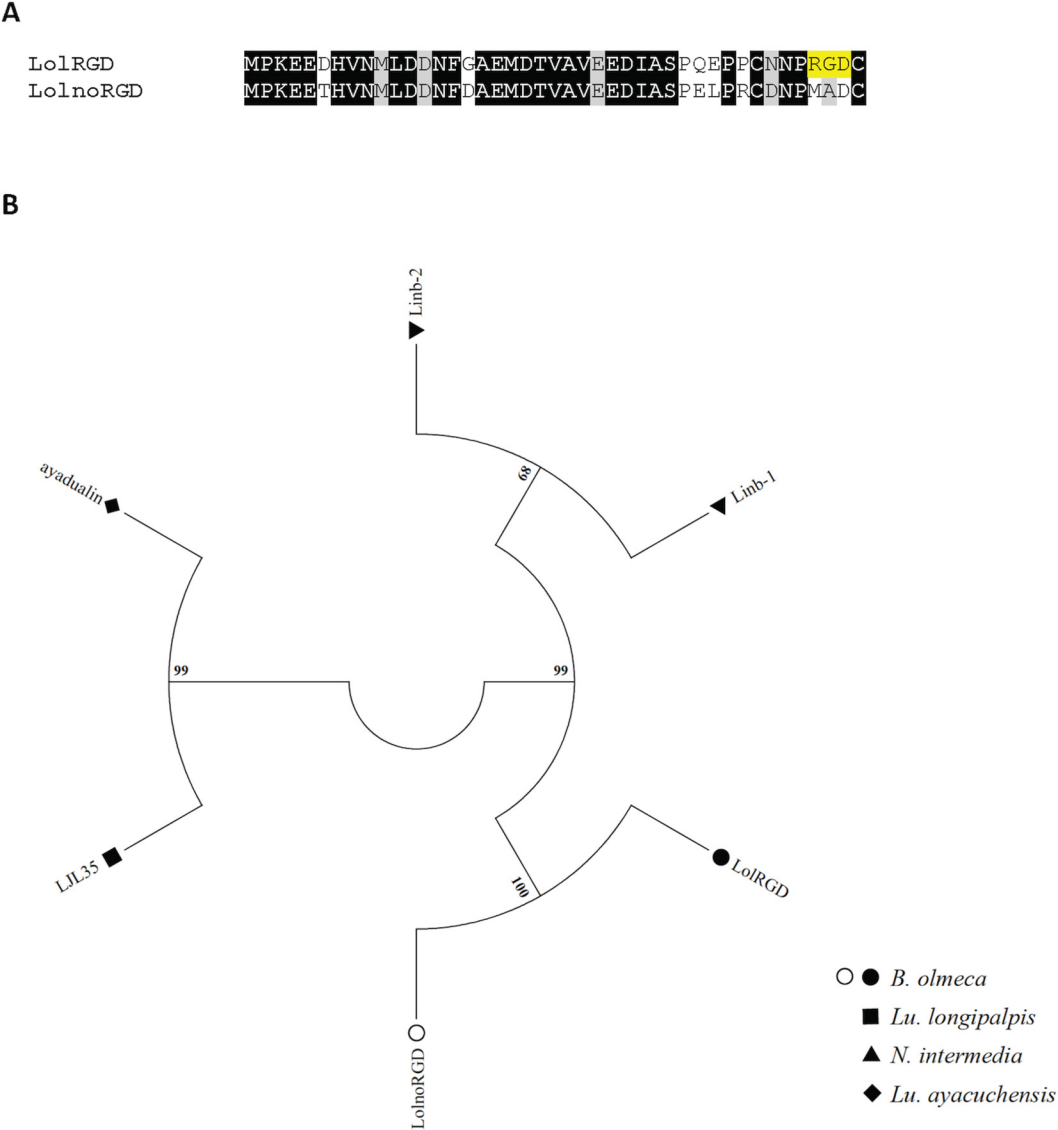
Multiple sequence alignment and molecular phylogenetic analysis of the sand fly RGD protein family. (**A)** Alignment of the LolRGD and the LolnoRGD identified from the *B*. *olmeca* salivary gland transcriptome. Black background shading represents identical amino acids. Grey background shading represents similar amino acids. Yellow background shading highlights the RGD motif. (**B**) The RGD protein phylogeny depicts three distinct branched, one encompassing *N*. *intermedia* sequences, another one including *Lu*. *longipalpis* and *Lu*. *ayacuchensis* proteins, and a third clade unique to *B*. *olmeca*, which includes the gene copy devoid of the RGD motif as well as the one containing such a motif. The evolutionary history was inferred based on the Whelan And Goldman model [62]. Sand fly species are indicated by the different symbols in the legend on the right.

Peptides containing RGD (Arginine-Glycine-Aspartate) sequence motifs have been previously identified in the salivary gland of New World sand flies: *Lu*. *longipalpis* [18], *N*. *intermedia* [20], and *Lu*. *ayacuchensis* [19]. Recently, Kato and colleagues (2015) described a dual role for a *Lu*. *ayacuchensis* RGD containing protein called Ayadualin [32]. This protein inhibits platelet aggregation by binding to the integrin α_IIb_β_3_, an RGD-dependent function, and prevents blood coagulation by targeting FXII activation, an RGD-independent role [32]. In *B*. *olmeca* salivary gland cDNA library, two related gene copies were identified (**Fig 2A**); one displays a RGD motif (LolRGD) at the carboxy-terminal end but the other is devoid of such a motif (LolnoRGD) (**Table 3**). Interestingly, LolnoRGD is the first described salivary proteins from this family to lack a RDG motif, contrasting with all homologs already described in other New World sand flies. Phylogenetic tree analysis shows that the *B*. *olmeca* RGD-like sequences are more similar to each other than to RGD proteins from other sand fly species (**Fig 2B**). The RGD family of *B*. *olmeca* cluster together with homologs of *N*. *intermedia*. On the other hand, *Lu*. *longipalpis* and *Lu*. *ayacuchensis* RGD homologs cluster together in a distinct clade (**Fig 2B**). The emergence of LolnoRGD in *B*. *olmeca* might be a specialization to prevent only blood coagulation, whereas LolRGD may prevent both platelet aggregation and blood coagulation.

#### 5kDa protein family (non-Maxadilan-like)

**Fig 3:**
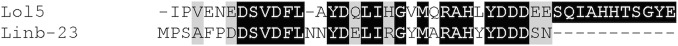
Multiple sequence alignment of the sand fly 5kD protein family. Multiple sequence alignment of the Lol5 identified from the *B*. *olmeca* salivary gland transcriptome and Linb-23 from *N*. *intermedia*. Black background shading represents identical amino acids. Grey background shading represents similar amino acids.

Proteins belonging to the 5kDa family have been formerly identified only in the salivary gland of *N*. *intermedia* [20], but they were catalogued previously as a RGD family of proteins although they do not have an RGD sequence or have similarities to LolnoRGD. Therefore, we are reassigning them to the 5kDa protein family. One member of this 5kDa protein family was identified in *B*. *olmeca* salivary gland cDNA library (**Table 4**). Multiple sequence alignment displays the similarities between such *B*. *olmeca* sequence (Lol5) and the *N*. *intermedia* proteins Linb-23 (**Fig 3**). These molecules seem to be unique to sand flies of the subgenera Nyssomyia and Bichromomyia and their biological function still remains to be determined.

#### C-type lectin-like protein family

This family of proteins is only found in New World sand flies and its signature is the presence of a C-type lectin domain, their molecular weight ranging from 13kDa to 17kDa (**Tables 1 and 5**). In the *B*. *olmeca* salivary gland transcriptome, five C-Type lectin homologs were identified and named LolCTLa, LolCTLb, LolCTLc, LolCTLd and LolCTLe. The multiple sequence alignment of sand fly C-type lectin-like salivary proteins revealed low amino acid similarities among the homolog sequences, yet four stereotypical cysteine residues were identified in an addition to a conserved ligand binding surface which is an essential part of the carbohydrate binding domain (**S4A Fig)**. These same cysteines residues were conserved in C-Type lectin-like proteins from *Lu*. *longipalpis*, *Lu*. *ayacuchensis* and *N*. *intermedia*, and also with a protein named tfiid from *Corethrella appendiculata* a frog-biting fly (**S4B Fig)**. The amino acid consensus signature for the salivary C-type lectin family is CX_70-91_CX_8-14_CX_4_CX_7_C. The phylogenetic analysis highlights the existence of multiple C-Type lectin lineages (**S4C Fig)**. In the clade shared only by *N*. *intermedia* and *B*. *olmeca* species as well as in the clades unique to *Lu*. *longipalpis* and *B*. *olmeca*, gene duplication events have taken place after the emergence of such species (**S4C Fig)**. In addition, the expression of C-type lectin-like transcripts in phylogenetic divergent species such as sand flies and culicoids may be due to neofunctionalization [33,34] of paralogs, which had the expression diverted to the salivary glands independently in each species. The function of the C-type lectin-like proteins from the saliva of sand flies remains to be characterized.

#### 14.2 kDa protein family

This family of proteins found only in sand flies of New World was described in *Lu*. *longipalpis* and *N*. *intermedia* as a 14.2kDa protein and its function remains to be elucidated. This protein family is represented by three homologues in the *B*. *olmeca* cDNA library that are dissimilar to each other in protein sequence, displaying only four conserved cysteine residues (**Table 6, S5A Fig**). Hence, the consensus cysteine signature of such family of salivary proteins is CX_11-13_CX_18-21_CX_12-14_C (**S5A Fig**). The phylogenetic relationship among members of such a family revealed two main clades: one encompassing *Lu*. *longipalpis* and *B*. *olmeca* proteins and the other composed of *B*. *olmeca* and *N*. *intermedia* counterparts (**S5B Fig)**. As *Lu*. *ayacuchensis* is devoid of 14.2kDa transcripts, and sequences of *Lu*. *longipalpis* and *N*. *intermedia* are lacking in either one of the clades, 14.2kDa protein gene losses have occurred in such species. On the other hand, not only was the aforementioned gene loss avoided in *B*. *olmeca* but also a unique *B*. *olmeca* duplication event might have given rise to Lol14.2b and Lol14.2c. The latter two proteins are identical in the N-terminus part but display very divergent C-termini, which could also point to alternative splicing (**S5A Fig)**. The function of this family of salivary proteins remains to be elucidated.

#### ML domain protein family

**Fig 4:**
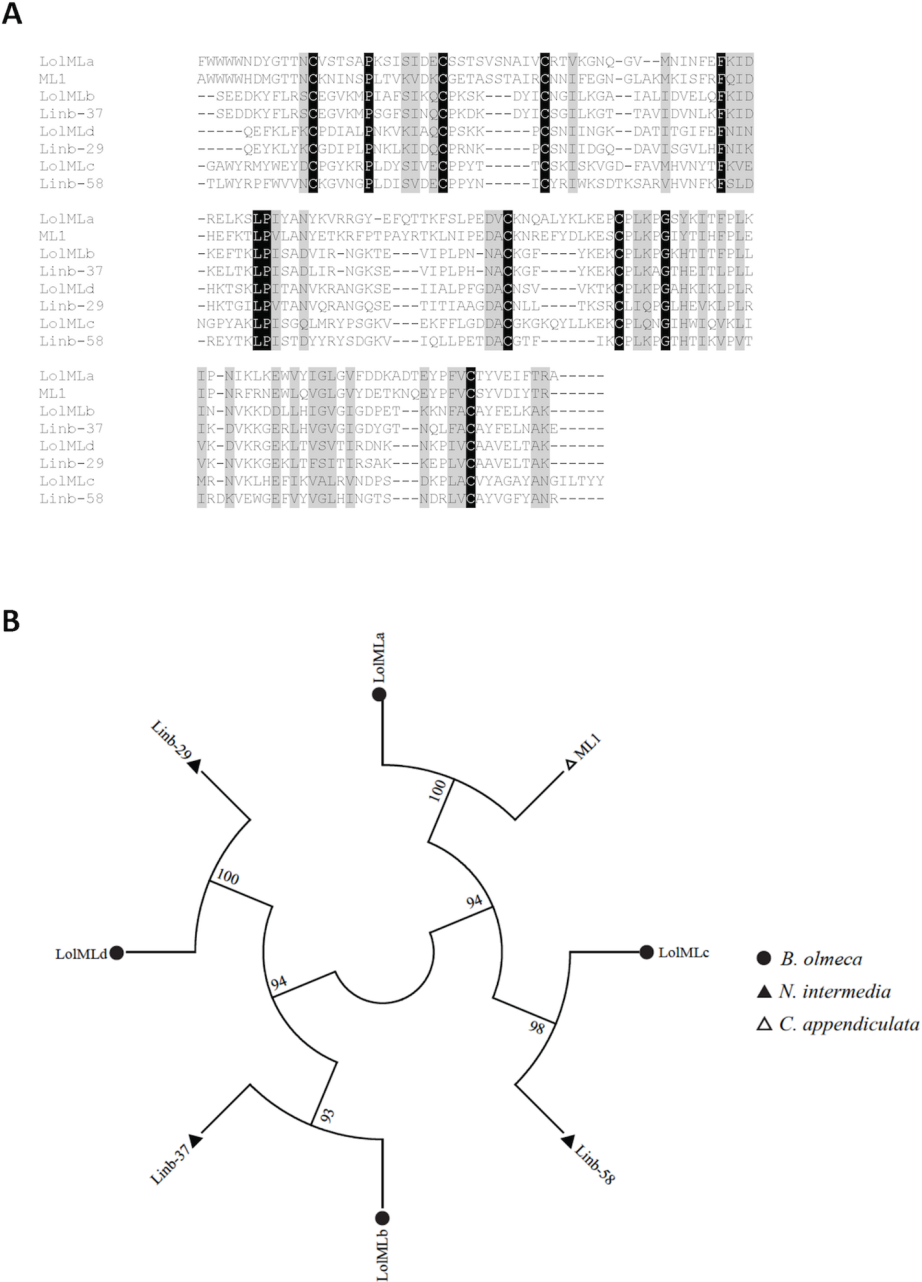
Multiple sequence alignment and molecular phylogenetic analysis of the salivary sand fly ML domain protein family. (**A**) Multiple sequence alignment of the four different ML-domain-like proteins (LolMLa-d) identified from the *B*. *olmeca* salivary gland transcriptome with similar proteins described from *N*. *intermedia* (Linb29, 37 and 58), and ML1 from a frog bitting fly *Corethrella apppendiculata*. Black background shading represents identical amino acids. Grey background shading represents similar amino acids. **(B)** The phylogenetic tree depicts four different branches, three of which encompass ML domain orthologs in *B*. *olmeca* and *N*. *intermedia* and one such a clade containing the ML domain protein of the frog biting fly and the counterpart in *B*. *olmeca*. The evolutionary history was inferred based on the Whelan And Goldman model [62]. Sand fly species are indicated by the different symbols in the legend on the right.

The ML domain or MD-2-related lipid recognition (ML) domain is present in proteins from different organisms: plants, animals, fungus and insects. The protein containing this a domain is very common in tick salivary gland transcriptomes [35]. Members of the ML family of proteins are involved in lipopolysaccharide signaling and lipid recognition. Representative of these lipid-binding proteins include the mammalian secretory protein (Human Epididymal secretory protein E1) HE1 known to bind cholesterol [36] and MD-2 that binds to lipopolysaccharides [37]. In sand flies, ML domain containing proteins was first described in *N*. *intermedia* [20]. In the current *B*. *olmeca* salivary gland library we identified four members of the ML family of proteins (**S6 Fig)**, sharing for the most part orthologs with *N*. *intermedia* ML-domain protein transcripts (**Fig 4A**). One of these proteins is a homolog of the ML-domain protein (ML1) expressed in the salivary glands of *Corethrella appendiculata*, a frog-biting fly [38] (**Table 7, Fig 4A**). Proteins of the ML family display 6 cysteine residues, following the consensus cysteine signature CX_13_CX_5-10_CX_42-47_CX_5-11_CX_39-43_C. The multiple sequence alignment shows six conserved cysteines and similar amino acid residues (**Fig 4A**). *B*. *olmeca* LolMLb, LolMLc, and LolMLd cluster independently with *N*. *intermedia* counterparts (**Fig 4B**). A single clade is shared by *B*. *olmeca* LolMLa and *C*. *appendiculata* ML1 (**Fig 4B**). It seems that ML domain proteins have emerged in an ancestor common to Bichromomyia and Nyssomyia, and independently in *C*. *appendiculata*. Thereby, the expression of similar transcripts in phylogenetic divergent species may be another case of neofunctionalization [33,34]. The biological function of this family of proteins in arthropods, including sand flies, remains to be elucidated.

#### Spider-toxin like proteins

**Fig 5:**
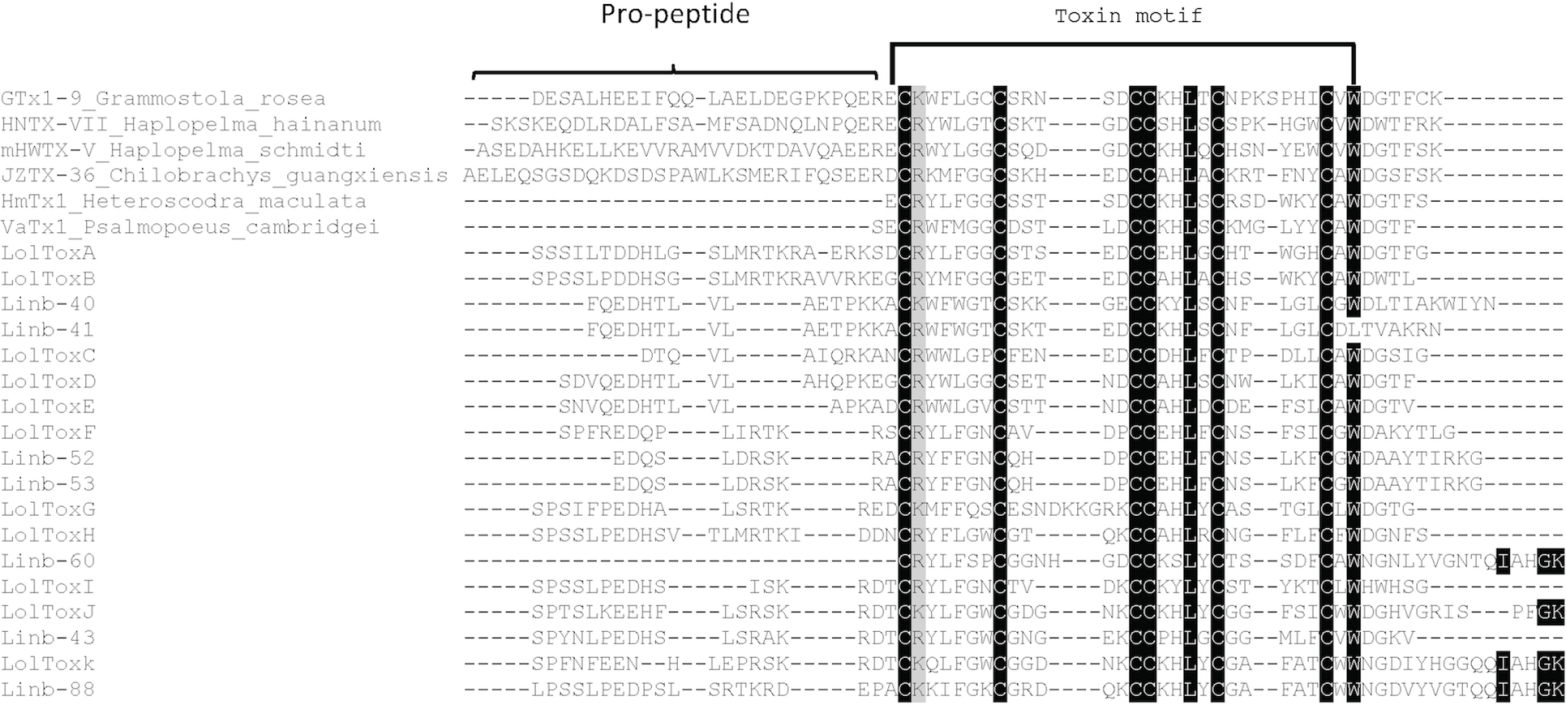
Multiple sequence alignment of the salivary sand fly Toxin-like protein family. Multiple sequence alignment of the Toxin-like protein from *B*. *olmeca* (LolToxA-K) with homologs from *N*. *intermedia* (Linb-40, 41, 43, 52, 53, 60 and 88), and from other spiders *Grammostola rosea*, *Haplopelma hainanum*, *Haplopelma schmidti*, *Chilobrachys guangxiensis*, *Heteroscodra maculate* and *Psalmopoeus cambridgei*. Brackets indicate the pro-peptide. And Toxin conserved motif is represented by half a rectangle. Black background shading represents identical amino acids. Grey background shading represents similar amino acids.

Toxin like proteins have been identified only in the salivary gland of *N*. *intermedia* [20], they are short proteins with a molecular weight ranging from 4 kDa to 6 kDa. Spider toxins have been described as agonists and antagonists of cationic channels [39–41]. Whereas some spider toxins are capable of blocking potassium channels causing pray paralysis [40,41], others are activators of the capsaicin receptor (TRPV1), eliciting predator pain and inflammation [39]. It may be possible that sand fly spider-like toxins could cause local muscle paralysis or antagonize pain if their function is similar to spider’s protein, which would prevent the sand fly from being perceived by the vertebrate during blood feeding. In the *B*. *olmeca* salivary gland cDNA library, transcripts coding for 11 related proteins (spider-toxin like peptides) of approximately 6kDa were identified and named LolToxA, LolToxB, LolToxC, LolToxD, LolToxE, LolToxF, LolToxG, LolToxH, LolToxI, LolToxJ and LolToxK (**Table 8**). The spider toxin-like peptides identified in the saliva of New World sand flies show similarities to the family E of spider toxins [42]. Toxins of the E family display both pro-peptide and mature peptide sequences, the latter portion being the pharmacologically active compound (**Fig 5**). In sand flies, this family of peptides seems to be unique to New World sand flies of the genera Nyssomyia and Bichromomyia, as homologs are only shared with *N*. *intermedia* (**Fig 5**). The sand fly spider-like toxins display six conserved cysteine residues and follow the stereotypical cysteine signature CX_6_CX_4-9_CCX_4_CX_5_C. Such a cysteine residue signature is highly conserved when compared with spider protein sequences (**Fig 5**), and contrasts to the high degree of polymorphisms among the non-cysteine amino acid residues (**Fig 5**). The presence of eleven toxin-encoding genes in *B*. *olmeca* and seven homologs in *N*. *intermedia* point to multiple events of gene expansion after the emergence in such species. Also, the expression of genes encoding such proteins in sand flies salivary glands and spider venom glands likely emerged independently; therefore, expression of such similar genes in both organisms would represent another case of neofunctionalization [33,34].

### Proteins shared by New World and Old World species

Using the transcripts from *B*. *olmeca* cDNA library and transcriptomes from salivary gland of other sand fly species we have identified seven families of proteins that are shared between New World and Old World sand flies (**Table 17)**. These include:

#### Small odorant binding protein-like families

These families of proteins seem to be specific to saliva of sand flies, as they have not been reported in any other blood-sucking insect [11]. One member of this family of proteins, PpSP15 from the sand fly *Phlebotomus papatasi*, was previously shown to induce an immune response that protect mice against *L*. *major* infection [4]. In a recent study its homolog in *P*. *duboscqi* (PdSP15) was shown to protect nonhuman primates against cutaneous leishmaniasis (CL) transmitted by *L*. *major* infected *P*. *duboscqi* bites [5]. Linb-7, a salivary protein from *N*. *intermedia* and member of this family of proteins was shown to produce a strong humoral and cellular immune response in mice [20]. *P*. *duboscqi* PdSP15 was recently shown to be an inhibitor of the contact activation of the coagulation cascade and to bind heparin and negatively charged molecules, in addition to its protective role against CL [43].

Members of the Small OBP-like protein family are present across the New World (**S7A Fig)** and Old World sand flies (**S7B Fig**) and bear multiple identical and similar amino acids along with six stereotypical cysteine residues. The consensus cysteine signature for the Small OBP-like protein is CX_10_CX_3_CX_46_CX_15_CX_8_ (**S7A and S7B Fig)**. A phylogenetic tree of sequence that have more than 40% identity shows two main clades: one encompassing New World sand fly Small OBP-like sequences and the other Old World sand fly ones (**S7C Fig)** [11,13,17] pointing out that the genes that encode small OBP-like proteins diverged in New World and Old World sand flies after the split from a common ancestor (**S7C Fig)**. In the New World sand fly branch, three distinct sub-clades are noticed (**S7C Fig)**. Whereas two such sub-clades were shared by proteins of *N*. *intermedia* and *B*. *olmeca*, the other sub-clade encompasses proteins belonging to members of the genus *Lutzomyia*: *Lu*. *longipalpis* and *Lu*. *ayacuchensis* (**S7C Fig)**. Clearly, the small OBP-like ortholog shared between species within the genus *Lutzomyia* and *N*. *intermedia* and *B*. *olmeca* was lost (**S7C Fig)**. On the other hand, gene expansion events have taken place after the emergence of the genera Bichromomyia and Nyssomyia, as noticed by the existence of multiple paralogs in *B*. *olmeca* (LolSOBPa, LolSOBPb and LolSOBPc) (**Table 9**) and *N*. *intermedia* (Linb-7, Linb-8, Linb-28, and Linb-59). Intriguing, only one small OBP-like has been identified in *Lu*. *longipalpis* (LuloOBP) to date, which is most likely due to the limited number of sequences generated from the *Lu*. *longipalpis* sand fly library [18]. In the Old World sand fly branch, gene duplication events were only observed in taxonomic closely *P*. *sergenti* (PsSP15 and PsSP11) and *P*. *papatasi* (PPTSP14 and PPTSP12; **S7C Fig)**. Overall, the sand fly small OBP-like protein phylogeny resembles the sand fly species phylogeny [44], which displays New World and Old World sand flies in distinct clades as well as members of the Phlebotomus/Paraphlebotomus and Larroussius/Adlerius/Euphlebotomus subgenera as more closely related phylogenetically [44].

#### Large odorant binding/ D7 protein family

D7 proteins were identified as a blood coagulation inhibitor affecting the activation of the plasma contact system in the *Anopheles stephensi* saliva [45], and recently this protein was shown to effectively bind to Thromboxane A2 [46]. In *Anopheles gambiae* and *Aedes aegypti* the salivary D7 protein was shown to strongly bind biogenic amines including serotonin, histamine, and norepinephrine [47]. Amino acids essential for leukotriene binding activity identified in mosquitoes seem to be conserved in all sand fly D7 proteins (**S8A Fig)**. To date the function of sand fly D7 proteins remains to be elucidated in sand flies.

This family of proteins is expressed in salivary glands of all sand fly species [48]. In the *B*. *olmeca* salivary gland transcriptome, a single LolD7 protein was identified (**Table 10**). The D7 protein family sequences are characterized by the presence of ten stereotypical cysteine residues, following the CX_25-27_CX_3_CX_44-46_CX_49-50_CX_6-12_CX_3_CX_13-16_CX_9_CX_8_C consensus signature (**S8A Fig)**. The Phylogenetic analysis of the D7 protein family suggests considerably divergence among sand flies species [44] (**S8B Fig)**. The Phlebotomus/Paraphlebotomus clade is more similar to the New World sand fly D7 clade, than to Larroussius/Adlerius D7 clades (**S8B Fig**). Although, three clades belonged to the Old World sand flies of the sub-genera Larroussius and Adlerius, a single D7 protein lineage was observed in New World species and Old World sand flies belonging to the subgenera Phlebotomus and Paraphlebotomus, accounting for gene losses (**S8B Fig)**. For Larroussius/Adlerius subgenera, intra-specific gene duplication events were also noticed in the largest branch and are likely to have taken place after emergence of the species (**S8B Fig**). Thus, specific selective pressures might be shaping sand fly D7 protein-encoding genes so that the pattern of evolution of such gene families diverged considerably from the pattern observed for the sand fly species phylogeny [44]. The analysis of natural selection based of the rate of non-synonymous over synonymous replacements (ω) in the D7-protein encoding transcripts pointed out that the clade belonging to Phlebotomus/Paraphlebotomus subgenera displayed a coding region under positive selection (ω > 1) as well as another coding region under relaxed purifying selection (ω ≅ 1). Relaxed purifying selection was also noticed for the paralog sequences that out-grouped the main clade belonging to the Larroussius/Adlerius/Euphlebotomus subgenera as well as the New World sand fly clade (**S9 Fig**).

#### Antigen-5 protein family

Antigen-5 family of proteins belong to the cysteine rich secretory proteins, a family of proteins present in the saliva of most blood-sucking insects and also in hookworms [49]. Proteins of this family have been described as related to venom allergens in social wasps and ants [11,19] and many other blood-sucking insects [50]. In addition, their sequences are also highly conserved. Recently the activity of this family of proteins was characterized in kissing bugs [51]. Antigen 5 salivary protein from *Dipetalogaster maxima* and *Triatoma infestans* was shown to be a Cu(2+)-dependent antioxidant enzyme. The antioxidant activity of this salivary protein inhibits neutrophil oxidative burst and inhibits platelet aggregation [51]. These functions are yet to be unveiled for sand fly salivary Antigen-5 proteins.

In the *B*. *olmeca* transcriptome we identified a cluster coding for a full length Antigen-5-like protein (LolAg5) with a molecular weight close to 29kDa (**Table 11**). Multiple sequence alignment between LolAg5 and homologues in other sand fly species showed that these molecules are quite conserved (**S10A Fig**); the Antigen-5 sequences displayed 14 stereotypical conserved cysteine residues that follow the consensus signature CX_4_CX_9-13_CX_9-10_CX_59_CX_6_CX_5_CX_71_CX_18_CX_2_CX_15_CX_2_CX_4_CX_7_C. Phylogenetic analysis of the Antigen-5 protein followed the pattern of sand fly phylogeny [44] in which New World and Old World sequences branched apart, and where the Antigen-5 sequences of closely related species within these taxa clustered together (**S10B Fig**). For instance, for the Old World sand fly Antigen-5, one cluster encompassed sequences of the closely related Phlebotomus and Paraphlebotomus subgenera whereas the other included for the most part the Antigen-5 sequences specific to the subgenera Larroussius and Adlerius (**S10B Fig**). A single species-specific gene duplication event was noticed in *P*. *duboscqi*, pointing to gene expansion after emergence of such a species.

#### Silk-related protein family

**Fig 6:**
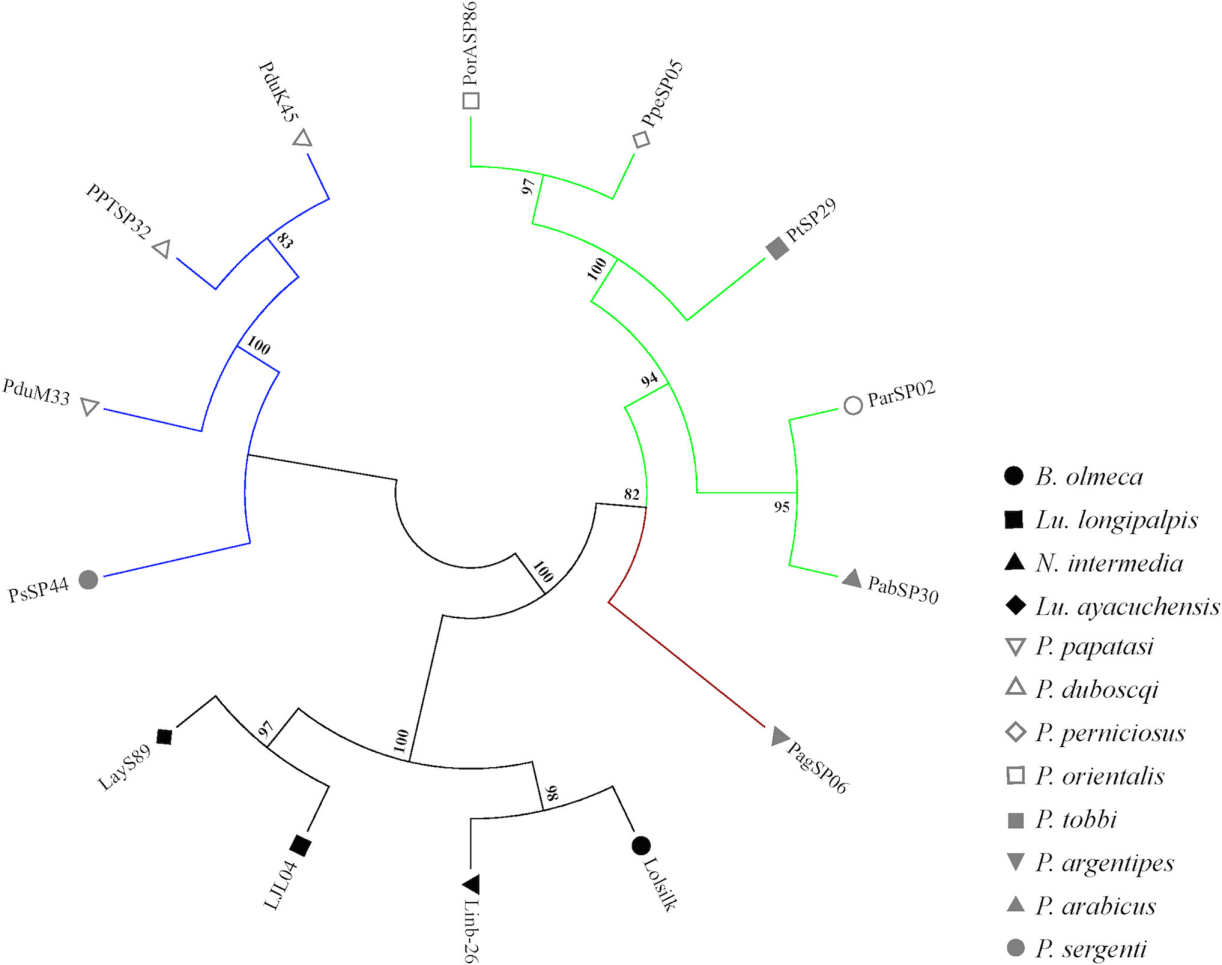
Molecular phylogenetic analysis of the salivary sand fly silk-related protein family. Phylogenetic analysis of the sand fly Silk protein family shows that proteins belonging to sand flies of the Larrossius sub-genus clustered together and were out-grouped by the Silk protein from the closely related *P*. *argentipes*. Such a clade is out-grouped by sequences belonging to New World sand flies; this large cluster is out-grouped by sequences belonging to sand flies of the Phlebotomus and Paraphlebotomus sub-genera. The evolutionary history of the sand fly Silk proteins was inferred based on the Jones et al. w/freq. model [63]. Sand fly species are indicated by different symbols. Tree branches were color-coded so as to represent specific taxa: Green color represents the Larroussius and Adlerius subgenera; Red color indicates the Euphlebotomus subgenus; Blue color points to proteins of the Phlebotomus and Paraphlebotomus subgenera; and Black color indicates the proteins belonging to New World sand flies.

Silk-related protein transcripts have been identified only in sand flies, no transcript coding for this protein has been reported in other blood-sucking insects to date. A member of the silk related protein in *P*. *papatasi*, the salivary protein PpSP32, was recently shown to be a biomarker for sand fly exposure and specifically for *P*. *papatasi* exposure [6]. Individuals bitten naturally by *P*. *papatasi* were shown to have specific antibodies to PpSP32 [6]. This salivary protein is becoming an important tool for epidemiological studies in areas where this sand fly species is prevalent [9]. A member of the Silk-related protein family (Lolsilk) was identified in the *B*. *olmeca* salivary gland transcriptome. Lolsilk encodes a protein of 21kDa (**Table 12**) with high similarity to a silk protein from the golden orb-web spider *Nephila clavipes* [11], and to collagen binding proteins [18]. Multiple sequence alignment between *B*. *olmeca* silk-related protein and its homologs in other sand fly species showed that these molecules are conserved only in the N- and C-termini domains of the protein and divergent in the middle segment (**S11 Fig)**. Phylogenetic analysis showed that New World sand fly Silk-related proteins cluster together in a different clade to those of Old World sand fly silk-related proteins (**Fig 6**). Interestingly, the Old World sand fly clade belonging to the subgenera Larroussius/Adlerius/Euphlebotomus is more closely related to that of New World sand flies than to the clade belonging to the Phlebotomus/Paraphlebotomus species (**Fig 6**). Thus, specific selective pressures might be shaping Silk-related protein-encoding genes (like the D7 gene family) so as to evolve in a different pattern to the observed for the sand fly species phylogeny [44]. In fact, sign of positive selection (ω > 1) were observed for some coding sequence stretches along the Silk-encoding sequence in the New World sand fly clade as well as in the Old World sand fly clades encompassing the Phlebotomus/Paraphlebotomus subgenera and Larroussius/Adlerius/Euphlebotomus subgenera (**S12 Fig**). Further studies are needed to determine if the silk related proteins from other sand fly species are also immunogenic and potentially be used as markers of sand fly exposure.

#### Yellow family of proteins

Yellow-related proteins are abundantly expressed in salivary glands of sand flies. The biological function of the *Lu*. *longipalpis* LJM11, LJM17 and LJM111 Yellow-related proteins was characterized as biogenic amine binding proteins [52]. Although those identified from *B*. *olmeca* share a few identical amino acids with yellow proteins from all other described species, the amino acids responsible for binding to biogenic amines are highly conserved (**S13 Fig**) [11,52]. This suggests that their function may be conserved in all sand flies species and working as anti-inflammatory agents. Additionally, *Lu*. *longipalpis* proteins from this family were described as biomarkers of sand fly exposure; people exposed to sand fly bites develop a high humoral immune response against LJM11 and LJM17 [8]. PpSP42 and PpSP44 yellow proteins from *P*. *papatasi* were first shown to prime a high humoral immune response and to exacerbate disease outcome in mice vaccinated with these proteins after challenge with *L*. *major* parasites together with sand fly saliva [53]. In contrast, LJM11 from *Lu*. *longipalpis* confers a protective cellular immunity in mice vaccinated with it and challenged with infected sand fly bites [54].

**Fig 7:**
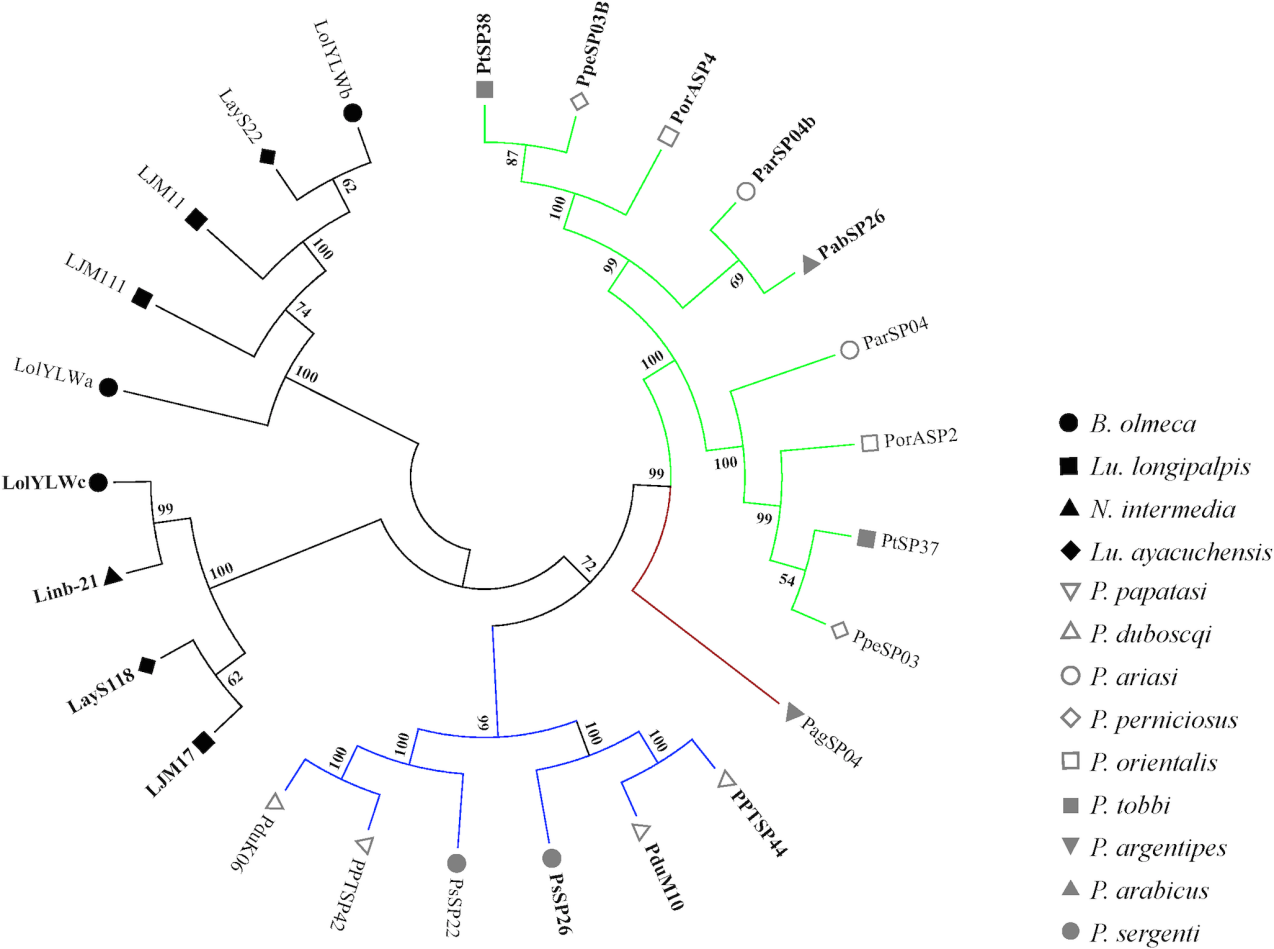
Molecular phylogenetic analysis of the salivary sand fly Yellow protein families. Phylogenetic tree shows a clear separation in distinct clades between the 42kDa and the 44kDa yellow protein. On the other hand, branches of yellow proteins tended to cluster together with branches containing their paralogs than orthologs, as clearly seem for the Old World sand fly sequences. The evolutionary history was inferred based on the Whelan and Goldman model [62]. Sand fly species are indicated by different symbols and follow the legend on the right. Tree branches were color-coded so as to represent specific taxa: Green color represents Larroussius and Adlerius subgenera; Red color indicates the Euphlebotomus subgenus; Blue color points to proteins of the Phlebotomus and Paraphlebotomus subgenera; and Black color indicates the proteins belonging to New World sand flies. Sequences belonging to the 44kDa protein family are highlighted in bold.

Three clusters of transcripts for the Yellow proteins were identified in the *B*. *olmeca* salivary gland transcriptome, LolYLWa, LolYLWb, LolYLWc (**S13 Fig**). Their putative molecular weights were 42.5 kDa (LolYLWa), 43kDa (LolYLWb), and 44.5 kDa (LolYLWc) (**Table 13**). Phylogenetic analysis depicted a clear separation between the 42kDa and 44kDa orthologs for both New World and Old World sand flies (**Fig 7**). For Old World sand flies, two main clades were shown, pertaining to sand flies of different subgenera: one clade belongs to the Phlebotomus and Paraphlebotomus subgenera and the other encompasses sequences belonging to the Larroussius, Euphlebotomus, and Adlerius subgenera. Overall, the Yellow-related proteins phylogeny is in accordance to the sand fly species phylogeny [44]. Interestingly, the Yellow proteins belonging to either New World or Old World sand flies were clustered in the same branches along with their paralogs, rather than within branches with their orthologs (**Fig 7**). This can be explained by either independent origins of the Yellow protein-encoding genes or by gene conversion. The latter seems to be a more parsimonious explanation. In fact, 19 gene conversion tracts were identified in paralogs of Old World sand fly 42kDa and 44kDa Yellow protein-encoding genes with an average length of 40.3 nucleotides (range 2–207) and ψ value of 0.065 [25]. In addition, the sequence similarity signatures between paralogs due to gene conversion have been reinforced by purifying selection (ω = 0.232; [24]), which is an important factor so as to maintain the gene conversion-derived similarities for longer periods of time [25]. Intra-specific paralogs were also noticed for *Lu*. *longipalpis* (LJM11 and LJM111), pointing to gene lineage expansions after emergence of such species.

#### Apyrase

Apyrase proteins are present in all blood-feeding arthropods tested so far including sand flies. The apyrase from sand flies saliva belongs to the Cimex family of apyrases [55,56]. Apyrases (EC 3.6.1.5) catalyze the hydrolysis of ATP and ADP into AMP and inorganic phosphate. The salivary sand flies apyrase activity is strictly calcium dependent and it has been shown to inhibit ADP-dependent platelet aggregation by destroying the agonist ADP [55–59].

In the *B*. *olmeca* salivary gland transcriptome, apyrase transcripts encode for a protein of 35kDa (**Table 14**). New World and Old World sand fly apyrases are conserved as shown by multiple sequence alignment (**S14A Fig**). Phylogenetic analysis shows that New World sand fly apyrases separate in a different clade from Old World sand fly sequences (**S14B Fig**), and it is overall in accordance to the sand fly phylogeny [44]. Overall, only one apyrase isoform is represented for each species. Nonetheless, events of intra-species gene duplication have taken place among the apyrase encoding genes in Old World sand fly *P*. *orientalis* (PorMSP4 and PorASP15) and *P*. *duboscqi* (PduM38 and PduM39), pointing to gene duplication after divergence.

#### Lufaxin-like protein family

**Fig 8:**
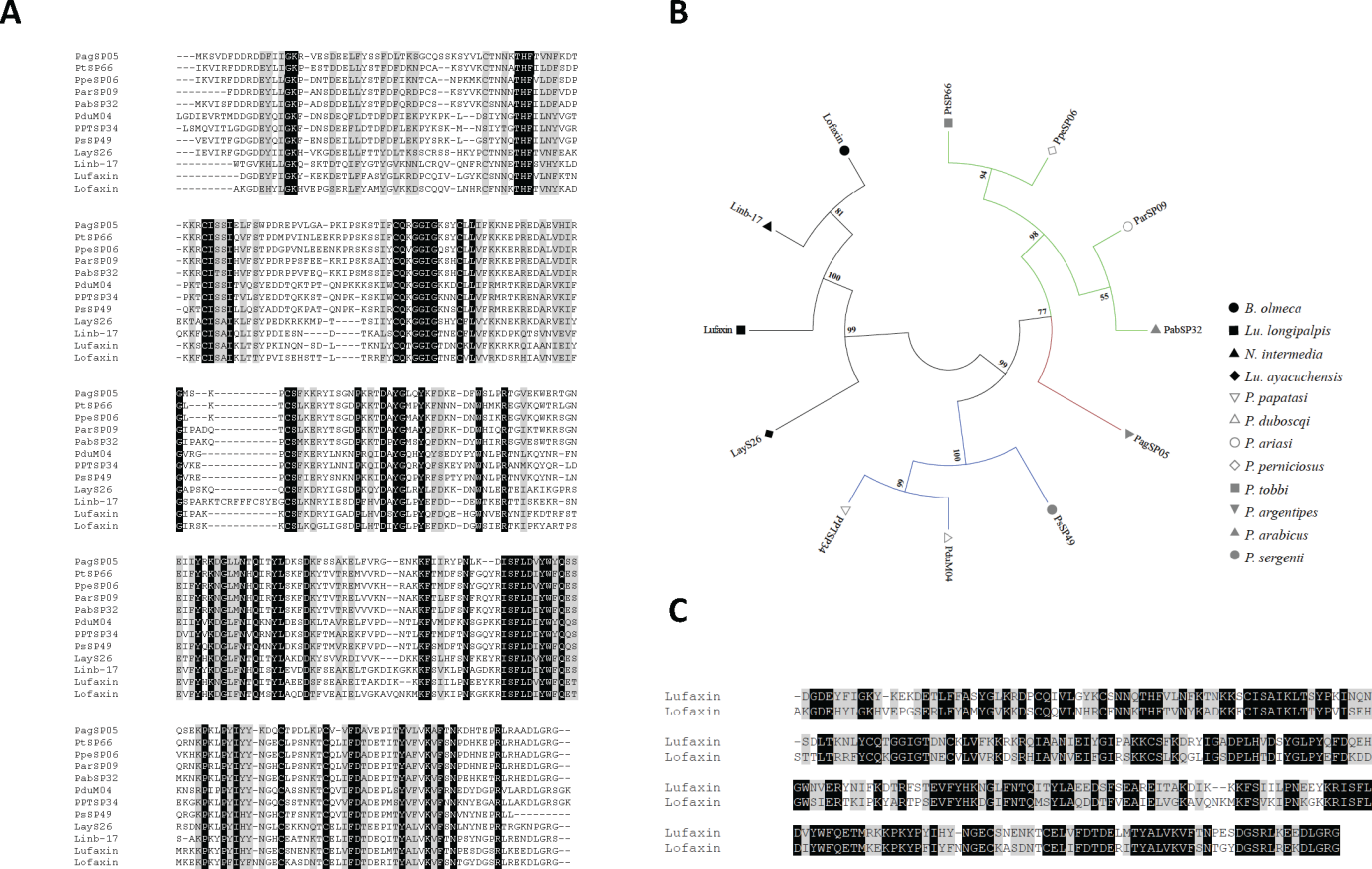
Multiple sequence alignment and molecular phylogenetic analysis of the salivary sand fly Lufaxin-like protein family. (**A**) Multiple sequence alignment of the Lufaxin-like protein from *B*. *olmeca* (Lofaxin) and the Lufaxin-like proteins reported to date. *Lu*. *longipalpis* (Lufaxin), *Lu*. *ayacuchensis* (LayS26), *N*. *intermedia* (Linb-17), *P*. *argentipes* (PagSP05), *P*. *ariasi* (ParSP09), *P*. *tobbi* (PtSP66), *P*. *perniciosus* (PpeSP06), *P*. *arabicus* (PabSP32), *P*. *sergenti* (PsSP49), *P*. *papatasi* (PPTSP34), *P*. *duboscqi* (PduM04). Black background shading represents identical amino acids. Grey background shading represents similar amino acids. (**B**) The Lufaxin phylogeny depicts a clear separation of New World and Old World sand flies in different clades as well as the split of Lufaxin proteins belonging to divergent subgenera in distinct branches. The evolutionary history based on the JTT matrix-based model [63]. Sand fly species are indicated by different symbols. Tree branches were color-coded so as to represent specific taxa: Green color represents the Larroussius and Adlerius subgenera; Red color indicates the Euphlebotomus subgenus; Blue color points to proteins of the Phlebotomus and Paraphlebotomus subgenera; and Black color indicates the proteins belonging to New World sand flies. (**C**) Alignment of Lufaxin from *Lu*. *longipalpis* with Lofaxin from saliva of *B*. *olmeca*. Black background shading represents identical amino acids. Grey background shading represents similar amino acids.

**Fig 9:**
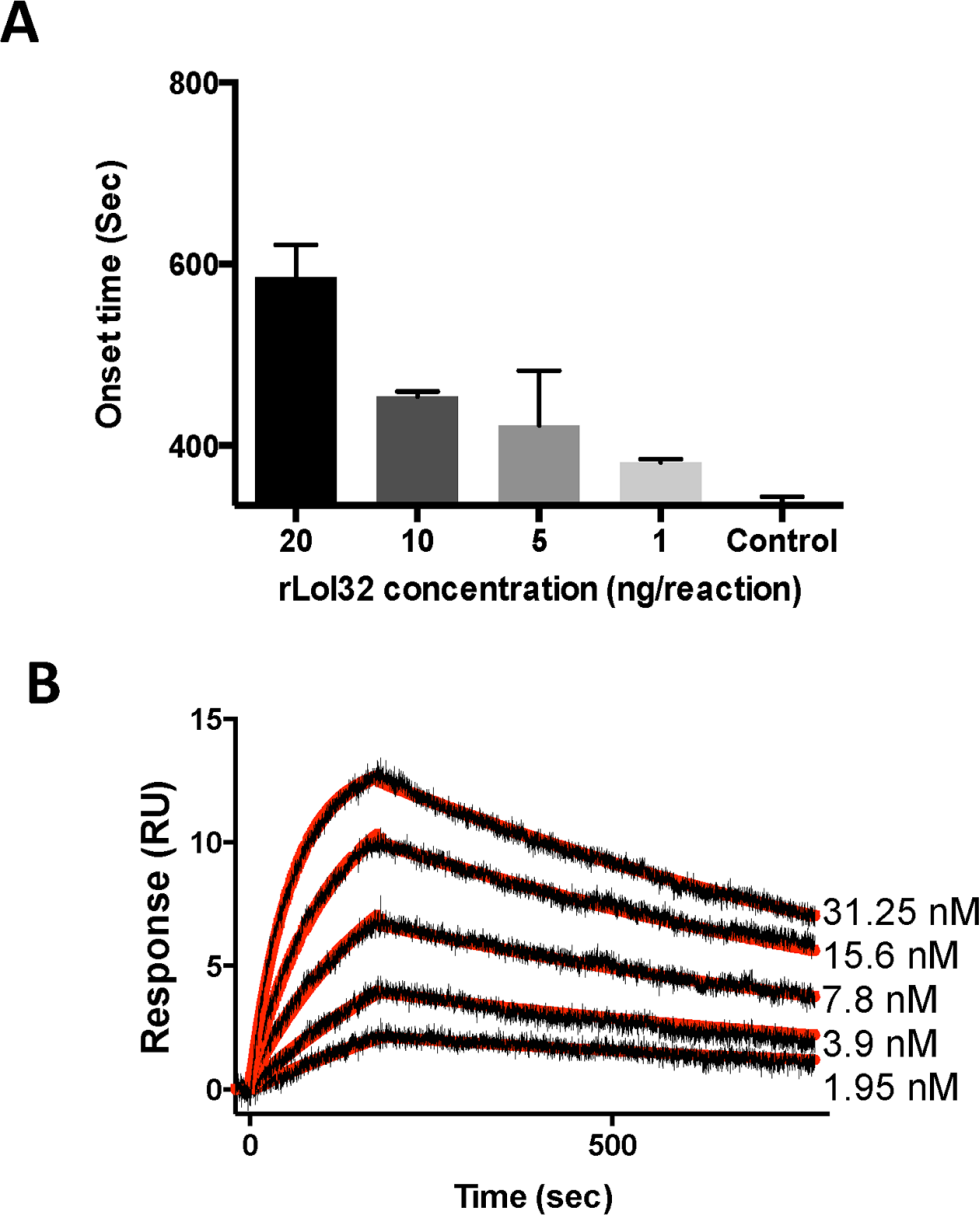
Lofaxin is an anticoagulant inhibiting Factor Xa. **(A)** Lofaxin inhibits the blood coagulation cascade in a dose dependent manner. Increasing amounts of Lofaxin (1–20 ng per reaction) increased the onset time (sec) of coagulation of human plasma. Human plasma alone is used as a negative control and Factor Xa with no proteins represents maximum coagulation. (B) Kinetics of Lofaxin interactions with immobilized FXa. Lofaxin binds to immobilized FXa at 31.25 nM (a), 15.6 nM (b), 7.8 nM (c), 3.9 nM (d) and 1.95 nM (e) as measured by SPR. Representative sensograms are shown in black, and fitting of the data points using the Langmuir equation is depicted in red.

The Lufaxin-like proteins are present in all sand fly species described to date, and have not been described in any other blood-sucking insect. The *Lu*. *longipalpis* salivary anti-coagulant Lufaxin is an effective factor Xa inhibitor [60], even though it is not highly abundant in the salivary gland of sand flies [18]. In the *B*. *olmeca* salivary gland transcriptome, we found a member of this family of proteins. This protein, named Lofaxin, has a putative molecular weight of 32.7kDa and an isoelectric point of 8.92 (**Table 15**). Protein sequence alignment showed that Lufaxin-like proteins of different sand fly species are very similar among each other (Mean identity 0.50; range 0.335–0.837) (**Fig 8A**). Phylogenetic analysis showed two main clades encompassing either New World or Old World sand fly Lufaxin-like proteins. Overall, the current Lufaxin-like protein phylogeny resembles the sand fly species phylogeny [44]. Lufaxin-like proteins from closely related species cluster together in sub-clades, for instance: Lofaxin and Linb-17 belong to the genera Nyssomyia and Bichromomyia; PPTSP43 and PduM04 belong to the subgenus Phlebotomus and are out-grouped by PsSP49 from the closely related subgenus Paraphlebotomus; and members of the Larroussius also cluster together and are out-grouped by PagSP05 from the closely related sub-genus Euphlebotomus (**Fig 8B**). Sequence alignment between Lufaxin from *Lu*. *longipalpis* and the *B*. *olmeca* Lufaxin-like protein shows these two proteins are very similar (Identity 0.58) (**Fig 8C**) suggesting *B*. *olmeca* Lofaxin may have the same anti-coagulant properties present in *Lu*. *longipalpis* Lufaxin. In order to test this hypothesis we expressed a recombinant Lofaxin (rLofaxin) in HEK cells and purified it by HPLC (**S15 Fig**). The purified rLofaxin increased the coagulation time (onset time) or time to form a clot in a dose dependent manner, demonstrating to be an inhibitor of the blood coagulation cascade (**Fig 9A**). To determine if Lofaxin also exerts its anticoagulant activity by binding to Factor Xa (similar to Lufaxin), we tested this potential interaction by Surface Plasmon Resonance (SPR). Factor Xa was immobilized in a carboxy-methylated dextran sensor chip, and rLofaxin was used as analyte. Sensograms of dose dependent binding between Factor Xa and rLofaxin are shown in (**Fig 9B)**. The equilibrium constant (KD) for Lofaxin and Factor Xa was calculated to be 1.83nM, very similar to the KD calculated for Lufaxin [60]. The fact that *B*. *olmeca* Lofaxin, has the same activity as Lufaxin, and the presence of this protein family in all sand fly species studied so far, suggests its role as an anticoagulant common to all sand flies, emphasizing conservation of function in a scenario of primary structure divergence.

### Comparative evolution of New World and Old World sand fly salivary proteins

**Fig 10:**
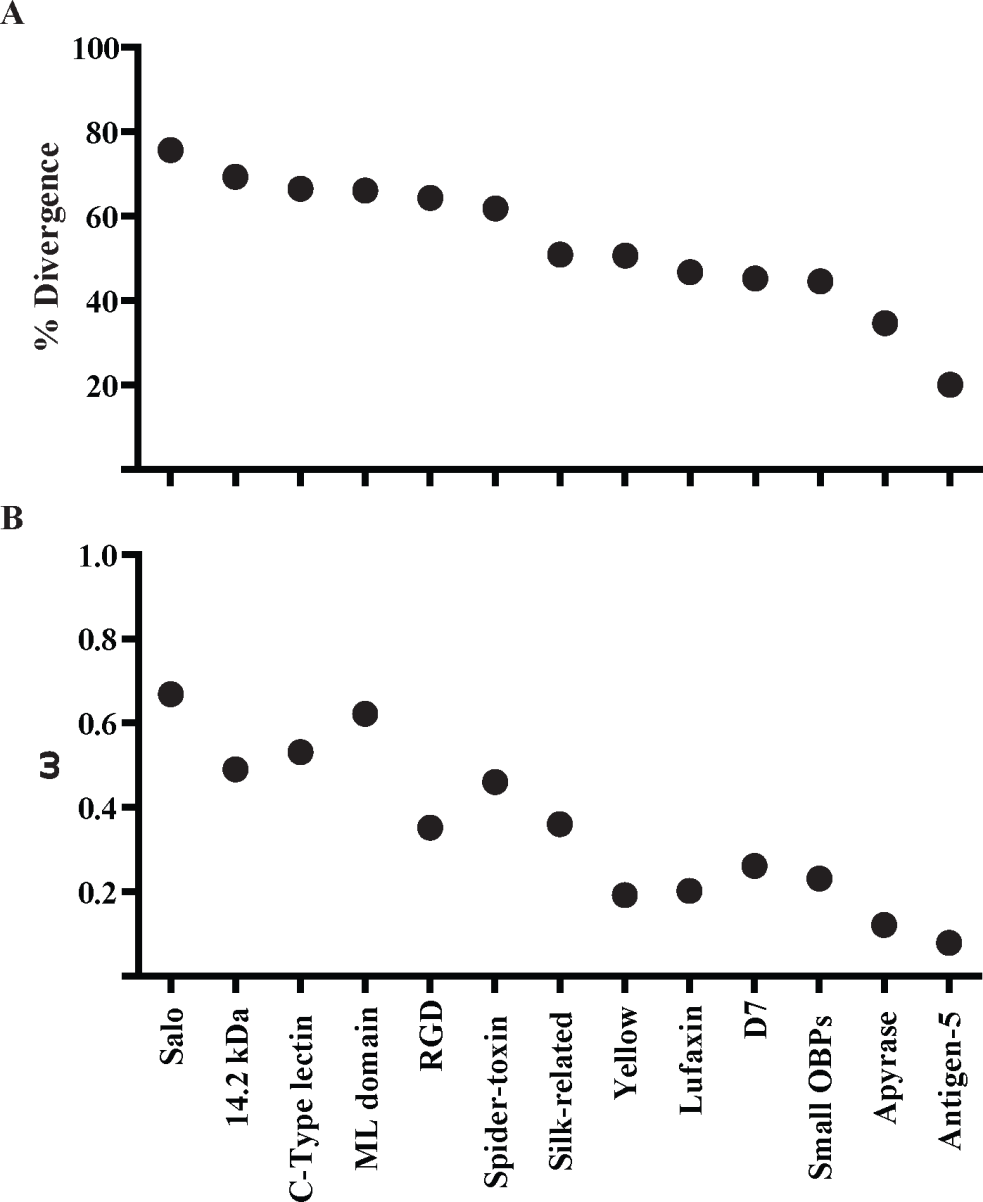
Evolutionary rate of salivary proteins in New World sand flies. The evolutionary properties of salivary proteins measured by **(A)** mean amino acid divergence (1—identity) and **(B)** the mean rate of non-synonymous over the rate of synonymous replacements (ω) of protein families unique to New World sand flies as well as the New World sand fly protein sequences shared with Old World sand flies.

**Fig 11:**
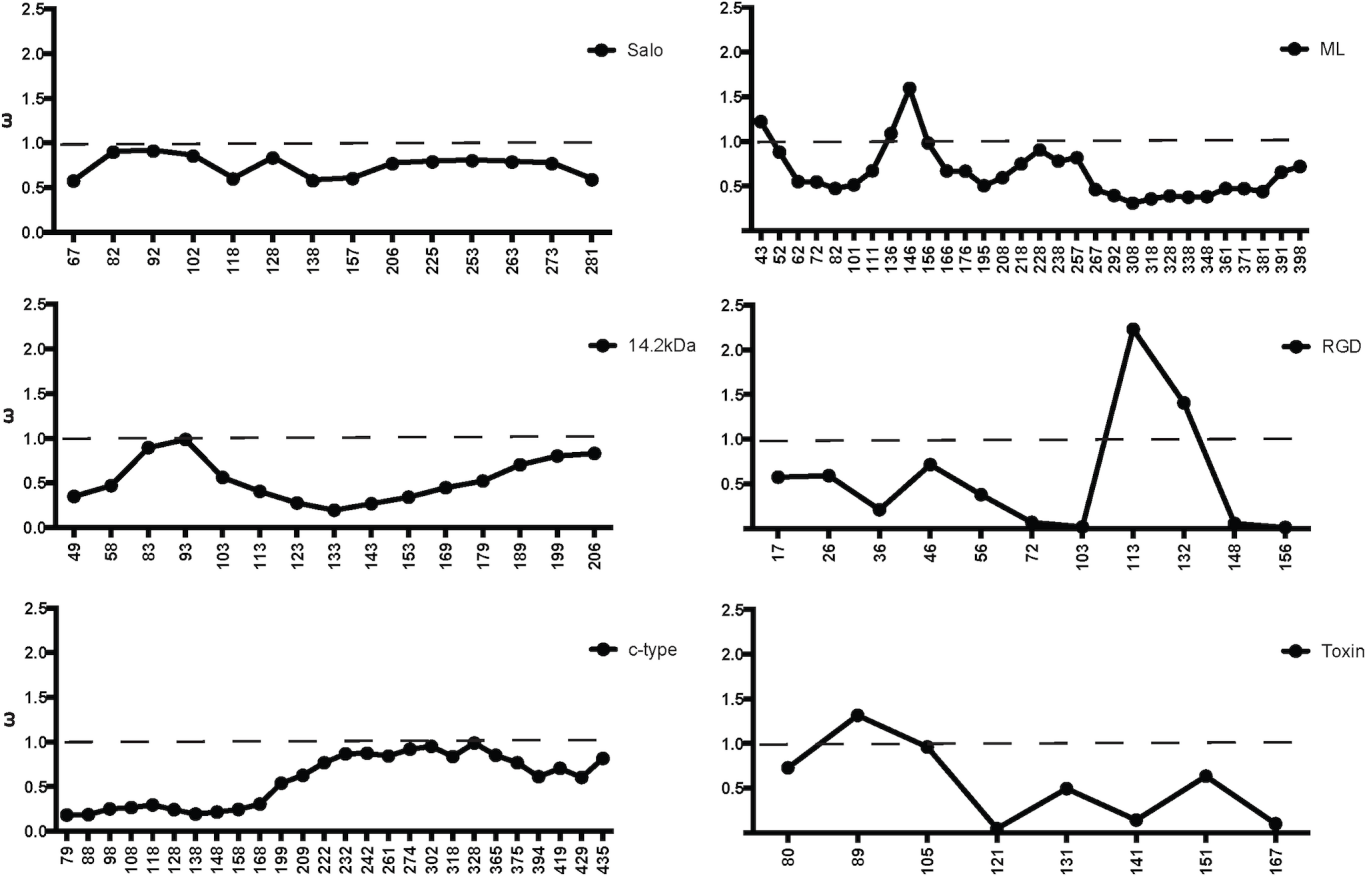
Evolutionary rate along length of salivary proteins in New World sand flies. Slide Window analyses of the ω values were performed for the protein families unique to New World sand flies. Dashed bars indicate the threshold for positive selection. X-axes indicate nucleotide positions.

In the current analysis of sand fly salivary transcriptomes, seven salivary protein families unique to New World sand flies and other seven proteins shared between New World and Old World sand flies were compared. Members of the sand fly salivary gland protein families unique to New World sand flies have been diverging at an overall faster rate at the sequence level (mean divergence 0.67; mean ω = 0.53; **Fig 10)** than the sequences shared with Old World sand flies (mean divergence 0.42; mean ω = 0.21; S15 Fig). By the same token, multiple nucleotide codons (or the overall sequences) of the genes encoding the proteins unique to New World sand flies are under positive (ω > 1) or relaxed purifying selection (ω ≅ 1; **Fig 11**), which is rarely seen for the protein families shared with Old World sand flies (**S16 Fig**). Also, multiple events of gene duplication (SALO, Spider Toxin-like, ML domain, C-type Lectin, Small OBP-like) are noticed across species for the genes that encode salivary proteins unique to New World sand flies. This contrasts to the very few events of gene duplication observed for salivary protein encoding genes shared with Old World species.

It is noteworthy that five out of seven protein families unique to New World sand flies display conserved cysteine residues (**S1 Table**). Such a pattern is in sharp contrast to the evolution of sand fly salivary proteins shared with Old World species; only three out of seven protein families bear conserved cysteine residues (**S1 Table**). This suggests that the *de novo* emergence of salivary gland protein families in New World sand flies seems to rely on cysteine building blocks.

Another interesting aspect of the salivary gland protein families unique to New World sand flies is the fact that proteins with similar primary structure are only shared with distantly related organisms, such as spiders (Spider Toxin-like) and the frog-biting fly *C*. *appendiculata* (ML domain and C-type Lectin). The expression of such proteins in the sand fly salivary glands and the emergence of the genes encoding such proteins may be due to neofunctionalization [33,34]. It is more parsimonious to assume that such genes expressed in such distantly related organism are derived from a common ancestral sequence that diverged after independent gene duplications to be expressed in the salivary glands than by *De novo* origin. For instance, there might be other C-type Lectin genes not expressed in the salivary gland that upon independent gene duplication events in each species gave rise to the C-type Lectin gene copies expressed in the salivary glands of sand flies and culicomorpha.

Among the protein families shared between New World and Old World sand fly species, most of them displayed phylogenies similar to the sand fly species phylogeny constructed based on the ITS-2 sequence [44]. In fact, natural selection has to be extremely strong relative to random drift to distort neutral genealogies [61]. Although the phylogeny for the Yellow family of proteins resembles the sand fly species phylogeny, the closer intra-taxa relationship between the Old World sand fly 42KDa and 44KDa proteins suggests that gene conversion might be shaping the genes encoding such proteins so that the intra-taxa paralogs look more similar to each other than to their inter-taxa orthologs. On the other hand, the D7 and Silk protein family phylogenies diverged considerably from the sand fly species phylogeny. Thereby, concerted evolution for the Yellow as well as positive and relaxed purifying selection for the D7 and Silk protein encoding genes have been driving the evolution of these protein so as to cope with the feeding habits of such sand fly species.

Although most of the salivary protein family phylogenies were similar to the sand fly species phylogeny, some clades have undergone faster protein diversification than others (**S17 Fig**). The Phlebotomus/Paraphlebotomus clades displayed similar to higher rates on non-synonymous replacements than the other clades (**S17 Fig**). Although signs of positive selection (ω > 1) are also noticed in a few codons for the Yellow family in the Phlebotomus/Paraphlebotomus clade (**S18 Fig**), protein divergence are likely overridden by gene conversion [34]. On the other hand, signs of positive selection were noticed only in the Phlebotomus/Paraphlebotomus clades in some codons for the protein families Antigen-5 and Apyrase. Contrasting to New World sand flies, some lineages of Old World sand flies rely on the diversification of more ancient protein families to adapt to new ecological niches.

Overall, the transcriptome of *B*. *olmeca* salivary glands and the comparative analysis between New World and Old World sand fly salivary proteins generated insights on a variety of molecular innovations that allowed for niche adaptations of the New World sand fly species to the American continent

## Supporting Information

S1 Fig Mexico map with an extension of the state of Tabasco. Sand flies were collected at the Cunduacan municipality, highlighted in grey.
(PDF)

S2 Fig Multiple sequence alignment of the sand fly SALO proteins. Multiple sequence alignment of the different SALO-like proteins (LolSALO) identified from the *B*. *olmeca* salivary gland transcriptome represented by (LolSALOa-f). Black background shading represents identical amino acids. Grey background shading represents similar amino acids.
(PDF)

S3 Fig Recombinant LolSALOa and LolSALOd have no effect on the complement classical pathway. Normal human serum (2.5%) was incubated with or without rSALO, rLolSALOa and LolSALOd (0.6 μM) and sensitized sheep erythrocytes (5 x 10^6^) for 30 min. For control reaction, sheep erythrocytes were incubated with human serum in the absence of the recombinant proteins and this condition was considered as 100% of hemolysis. Erythrocyte lysis was measured at 414 nm. Results are shown as mean +/- SD.
(PDF)

S4 Fig Multiple sequence alignment and molecular phylogenetic analysis of the sand fly C-type lectin protein family. (**A**) Multiple sequence alignment of the different C-type lectin-like proteins (LolCTLa-e) identified from the *B*. *olmeca* salivary gland transcriptome. Black background shading represents identical amino acids. Grey background shading represents similar amino acids. (**B**) Multiple sequence alignment of the sand fly C-type lectin protein families. Multiple sequence alignment of the different C-type lectin-like proteins (LolCTLa-e) identified from the *B*. *olmeca* salivary gland transcriptome with homologs identified from *Lu*. *longipalpis* (LJL18, LJL91, LJS142, LJM06), *Lu*. *ayacuchensis* (LayS127), *N*. *intermedia* (Linb-14, 15, 22, 48 and 63) sand flies and *C*. *appendiculata* (tfiid) frog-biting fly. Black background shading represents identical amino acids. Grey background shading represents similar amino acids. (**C**). Phylogenetic tree depicts multiple distinct branches containing C-type lectin orthologs in New World sand flies as well as multiple paralogs are noticed within the branches. The evolutionary history was inferred based on the Le Gascuel 2008 model [64]. Sand fly species are indicated by the different symbols in the legend on the right.
(PDF)

S5 Fig Multiple sequence alignment and molecular phylogenetic analysis of the sand fly 14.2kDa protein family. (**A**) Multiple sequence alignment of the different 14.2-like proteins (Lol14.2a-c) identified from the *B*. *olmeca* salivary gland transcriptome with homologs identified from *Lu*. *longipalpis* (LJM114) and *N*. *intermedia* (Linb-45) sand flies. Black background shading represents identical amino acids. Grey background shading represents similar amino acids. Gene alternative splicing was shown in the C-terminus part of the Lol14.2b and Lol14.2c sequences. (**B**) The phylogenetic tree shows two distinct branches, to which orthologs between *B*. *olmeca* and *N*. *intermedia* and *B*. *olmeca* and *Lu*. *longipalpis* belong. The evolutionary history was inferred based on the Whelan And Goldman model [62]. Sand fly species are indicated by the different symbols in the legend on the right.
(PDF)

S6 Fig Multiple sequence alignment of the salivary sand fly ML domain proteins. Multiple sequence alignment of the different ML-domain-like proteins (LolMLa-d) identified from the *B*. *olmeca* salivary gland transcriptome. Black background shading represents identical amino acids. Grey background shading represents similar amino acids.
(PDF)

S7 Fig Multiple sequence alignment and molecular phylogenetic analysis of the salivary sand fly Small Odorant Binding Protein family. (**A**) Multiple sequence alignment of the different small OBP proteins (LolSOBPa-c) identified from the *B*. *olmeca* salivary gland transcriptome with homologs identified from *Lu*. *longipalpis* (LuloOBP), *Lu*. *ayacuchensis* (LayS58) and *N*. *intermedia* (Linb-7, 8, 28 and 59) sand flies. Black background shading represents identical amino acids. Grey background shading represents similar amino acids. (**B**) Multiple sequence alignment of the different small OBP proteins (LolSOBPa-c) identified from the *B*. *olmeca* salivary gland transcriptome with homologs identified from New World and Old World sand flies. Black background shading represents identical amino acids. Grey background shading represents similar amino acids. (**C**) The phylogenetic analysis shows the split of New World and Old World sand fly proteins in distinct clades. Although multiple paralogs are noticed for the New World sand fly sequences, only *P*. *sergenti* displays a paralog amongst the Old world sand fly Small OBPs. The evolutionary history was inferred based on the Whelan And Goldman model [62]. Sand fly species are indicated by different symbols. Tree branches were color-coded so as to represent specific taxa: Green color represents the Larroussius and Adlerius subgenera; Red color indicates the Euphlebotomus subgenus; Blue color points to proteins of the Phlebotomus and Paraphlebotomus subgenera; and Black color indicates the proteins belonging to New World sand flies.
(PDF)

S8 Fig Multiple sequence alignment and molecular phylogenetic analysis of the salivary sand fly D7 protein family. (**A**) Multiple sequence alignment of the D7-like proteins (LolD7) identified from the *B*. *olmeca* salivary gland transcriptome with homologs identified from *Lu*. *longipalpis* (LJL13), *Lu*. *ayacuchensis* (LayS101) and *N*. *intermedia* (Linb-42) New World species and *P*. *arabicus* (PabSP20, 54, 59 and 84), *P*. *tobbi* (PtSP42, 54, 57 and 58), *P*. *papatasi* (PPTSP28a and 28c), *P*. *ariasi* (ParSP12 and 16), *P*. *perniciosus* (PpeSP04 and 04B), *P*. *orientalis* (PorMSP28, 38 and 43) and *P*. *sergenti* (PsSP4 and 7) Old World species. Black background shading represents identical amino acids. Grey background shading represents similar amino acids. * Indicates the essential amino acids for leukotriene binding activity in the mosquito D7 protein. (**B**) The phylogeny depicts a large clade encompassing D7 proteins of sand flies belonging to the Larrossius sub-genus, out-grouped by other clades belonging to either New World sand fly D7 proteins or their counterparts of members of the Phlebotomus and Paraphlebotomus sub-genera. All such sequences were out-grouped my D7 proteins belonging to sand flies of the Larrossius sub-genus. The evolutionary history was inferred according to the Jones et al. w/freq. model [63]. Sand fly species are indicated by different symbols. Tree branches were color-coded so as to represent specific taxa: Green color represents the Larroussius and Adlerius subgenera; Red color indicates the Euphlebotomus subgenus; Blue color points to proteins of the Phlebotomus and Paraphlebotomus subgenera; and Black color indicates the proteins belonging to New World sand flies.
(PDF)

S9 Fig Evolutionary rate along length of the D7 salivary protein family in sand flies. (**A**-**D**) Slide Window analyses of the ω values were performed for the D7 protein family in all sand flies (**A**) as well as only for the sequences belonging to the New World (**B**), Phlebotomus/Paraphlebotomus (**C**), Larroussius/Adlerius/Euphlebotomus (**D**), and the Larroussius/Adlerius/Euphlebotomus not belonging to the main clade (ω-L/A/E—outgrup). X-axes indicate nucleotide positions.
(PDF)

S10 Fig Multiple sequence alignment and molecular phylogenetic analysis of the salivary sand fly Antigen-5 protein family. (**A**) Multiple sequence alignment of the Antigen 5-like protein (LolAg5) identified from the *B*. *olmeca* salivary gland transcriptome with homologs identified from *Lu*. *longipalpis* (LuloAg5), *Lu*. *ayacuchensis* (LayS79) and *N*. *intermedia* (Linb-13), *P*. *ariasi* (ParSP05), *P*. *arabicus* (PabSP4), *P*. *orientalis* (PorASP74), *P*. *perniciosus* (PpeSP07), *P*. *argentipes* (PagSP05), *P*. *papatasi* (PPTSP29), *P*. *duboscqi* (PduM48, PduK107). Black background shading represents identical amino acids. Grey background shading represents similar amino acids. (**B**) The phylogenetic analysis displays New World and Old World sand flies in distinct branches. For the Old World sand flies, Antigen-5 protein belonging to sand flies of closely related sub-genera clustered together. The Whelan And Goldman model [62] was used to infer the evolutionary history of the sand fly Antigen-5 proteins. Sand fly species are indicated by different symbols. Tree branches were color-coded so as to represent specific taxa: Green color represents the Larroussius and Adlerius subgenera; Red color indicates the Euphlebotomus subgenus; Blue color points to proteins of the Phlebotomus and Paraphlebotomus subgenera; and Black color indicates the proteins belonging to New World sand flies.
(PDF)

S11 Fig Multiple sequence alignment of the salivary sand fly silk-related protein family. (**A**) Multiple sequence alignment of the Silk-related protein (Lolsilk) identified from the *B*. *olmeca* salivary gland transcriptome with homologs identified from *Lu*. *longipalpis* (LJL04), *Lu*. *ayacuchensis* (LayS89) and *N*. *intermedia* (Linb-26), *P*. *argentipes* (PagSP06), *P*. *tobi* (PtSP29), *P*. *perniciosus* (PpeSP05), *P*. *orientalis* (PorMSP15, PorASP86), *P*. *ariasi* (ParSP02), *P*. *arabicus* (PabSP30), *P*. *sergenti* (PsSP44), *P*. *duboscqi* (PduM33, PduK46), *P*. *papatasi* (PPTSP32). Black background shading represents identical amino acids. Grey background shading represents similar amino acids.
(PDF)

S12 Fig Evolutionary rate along length of the Silk salivary protein family in sand flies. (**A**-**D**) Slide Window analyses of the ω values were performed for the Silk protein family in all sand flies (**A**) as well as only for the sequences belonging to the New World (**B**), Phlebotomus/Paraphlebotomus (**C**), and Larroussius/Adlerius/Euphlebotomus (**D**) clades. X-axes indicate nucleotide positions.
(PDF)

S13 Fig Multiple sequence alignment of the salivary sand fly Yellow protein families. Multiple sequence alignment of yellow-related proteins from *B*. *olmeca* (LolYLWa-c) Black background shading represents identical amino acids. Grey background shading represents similar amino acids. * Indicates essential amino acids for binding biogenic amines.
(PDF)

S14 Fig Multiple sequence alignment and molecular phylogenetic analysis of the salivary sand fly Apyrase protein family. (**A**) Multiple sequence alignment of the Apyrase (LolApy) identified from the *B*. *olmeca* salivary gland transcriptome with homologs identified from *Lu*. *longipalpis* (LJL23), *Lu*. *ayacuchensis* (LayS17) and *N*. *intermedia* (Linb-35) New World species and *Psergenti* (PsSP42), *P*. *papatasi* (PPTSP36), *P*. *duboscqi* (PduM38 and 39), *P*. *argentipes* (PagSP03), *P*. *orientalis* (PorMSP3,4 and PorASP15), *P*. *perniciosus* (PpeSP01 and 01B), *P*. *tobbi* (PtSP4), *P*. *ariasi* (ParSP01) and *P*. *arabicus* (PabSP40) Old World species. Black background shading represents identical amino acids. Grey background shading represents similar amino acids. (**B)** Le_Gascuel_2008 model [64] was used to infer the evolutionary history of the sand fly Apyrase protein family. The phylogenetic tree depicted New World and Old World sand fly Apyrases in distinct branches. For the Old World sand flies Apyrases, sequences from phylogenetically close sand flies clustered together. Larroussius and Adlerius sequences are out-grouped by a Euphlebotomus one, which is out-grouped by Phlebotomus and Paraphlebotomus sequences. Sand fly species are indicated by different symbols. Tree branches were color-coded so as to represent specific taxa: Green color represents the Larroussius and Adlerius subgenera; Red color indicates the Euphlebotomus subgenus; Blue color points to proteins of the Phlebotomus and Paraphlebotomus subgenera; and Black color indicates the proteins belonging to New World sand flies.
(PDF)

S15 Fig Lofaxin protein Coomassie blue gel. 5ug of rLofaxin protein was run in a 4–12 NuPage gel, colored with coomassie stain for 1h and destained to show the purified protein as a single band.
(PDF)

S16 Fig Evolutionary rate along length of salivary proteins shared between New World and Old World sand flies. Slide Window analyses of the ω values were performed for the protein families shared between New World and Old World sand flies. Dashed bars indicate the threshold for positive selection. X-axes indicate nucleotide positions.
(PDF)

S17 Fig Evolutionary rate of salivary proteins for the families shared between New World and Old World sand flies. The mean rates of non-synonymous over the rate of synonymous replacements (ω) of protein families are depicted for the protein-encoding gene sequences of all sand flies (ω-All) as well as only for the sequences belonging to the New World (ω-NW), Phlebotomus/Paraphlebotomus (ω-P/P), and Larroussius/Adlerius/Euphlebotomus (ω-L/A/E) clades.
(PDF)

S18 Fig Evolutionary rate along length of the Yellow salivary protein family in sand flies. Slide Window analyses of the ω values were performed for the Yellow protein family for the sequences belonging to the Phlebotomus/Paraphlebotomus (ω-P/P) clades. Dashed bars indicate the threshold for positive selection. X-axes indicate nucleotide positions.
(PDF)

S1 Table Cysteine residue signatures among salivary protein families. The protein families that bear a scaffold of cysteine residues are listed as well as the number of cysteine residues, the presence or absence in New World and Old World sand flies, and the cysteine residue signatures.
(PDF)
