# Supplementary figures and images for: Molecular Diversity between Salivary Proteins from New World and Old World Sand Flies with Emphasis on Bichromomyia olmeca, the Sand Fly Vector of Leishmania mexicana in Mesoamerica
Source: PLoS Negl Trop Dis. 2016 Jul 13;10(7):e0004771. doi: 10.1371/journal.pntd.0004771 (PMC4943706; doi:10.1371/journal.pntd.0004771)

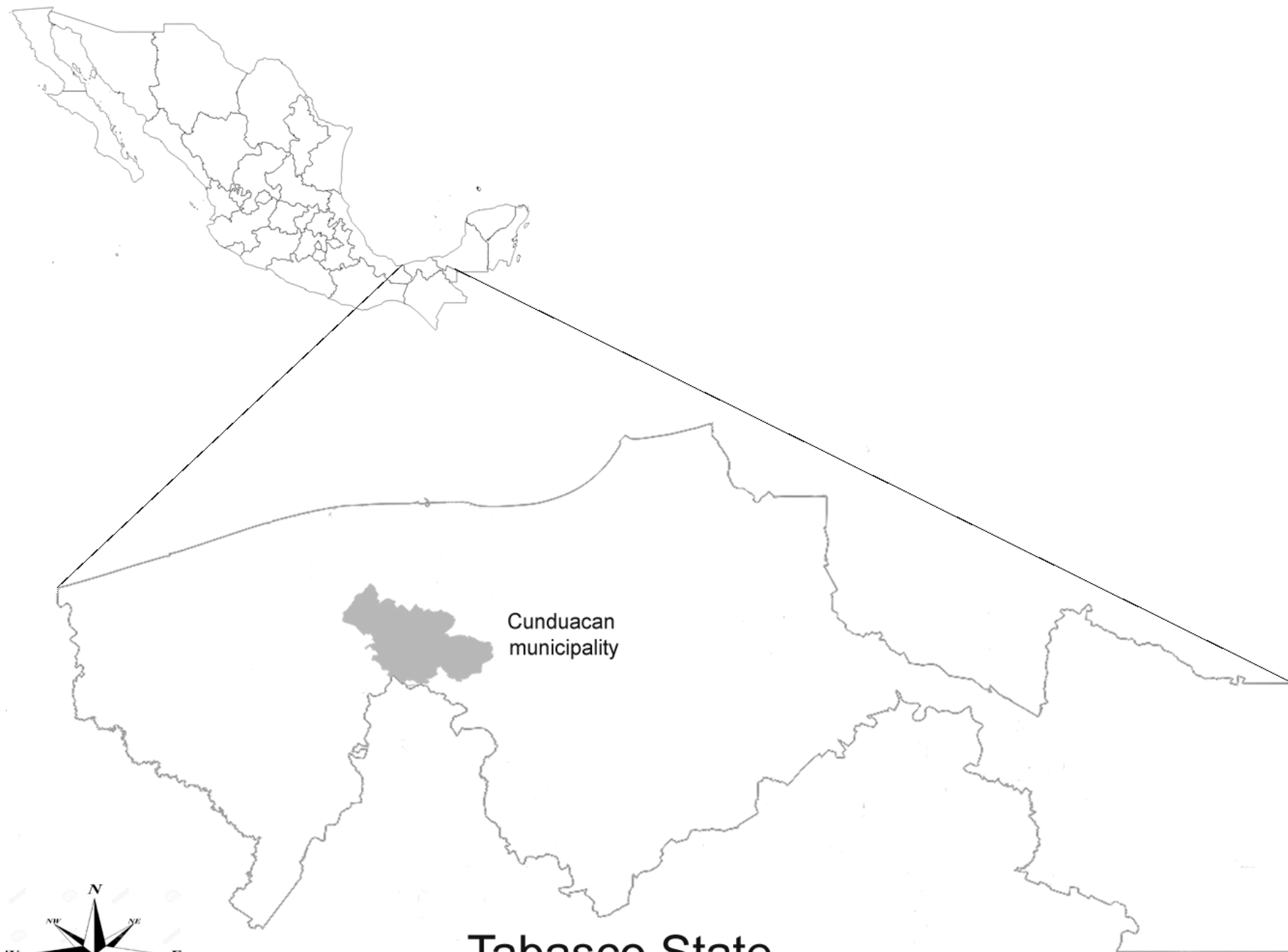

Cunduacan  
municipality

Tabasco State

1:500 000

Supplement: S1 Fig — Sand flies were collected at the Cunduacan municipality, highlighted in grey. (PDF) [file pntd.0004771.s001.pdf]

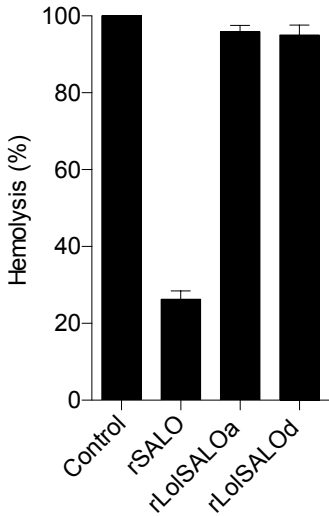

Supplement: S3 Fig — Normal human serum (2.5%) was incubated with or without rSALO, rLolSALOa and LolSALOd (0.6 μM) and sensitized sheep erythrocytes (5 x 10^6^) for 30 min. For control reaction, sheep erythrocytes were incubated with human serum in the absence of the recombinant proteins and this condition was considered as 100% of hemolysis. Erythrocyte lysis was measured at 414 nm. Results are shown as mean +/- SD. (PDF) [file pntd.0004771.s003.pdf]

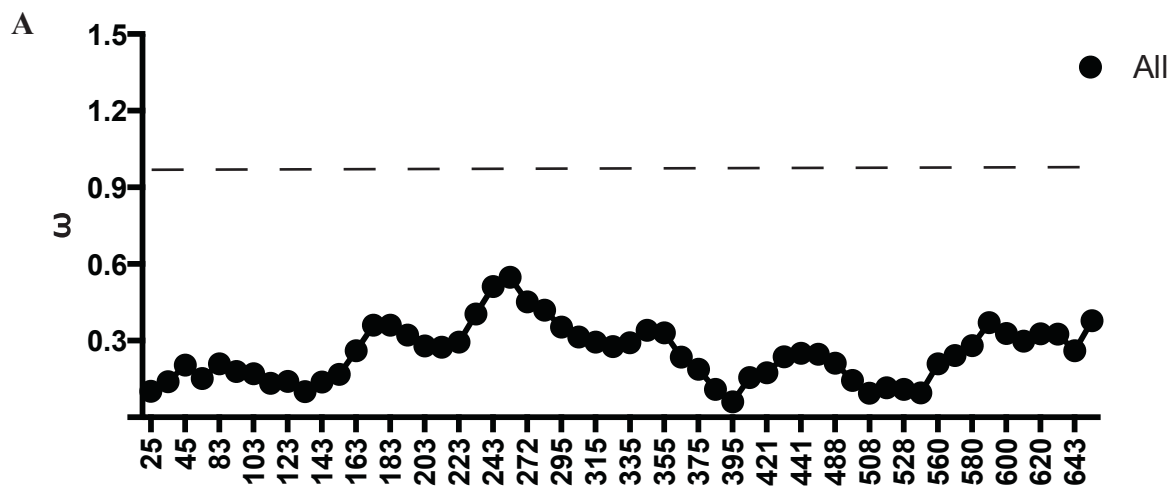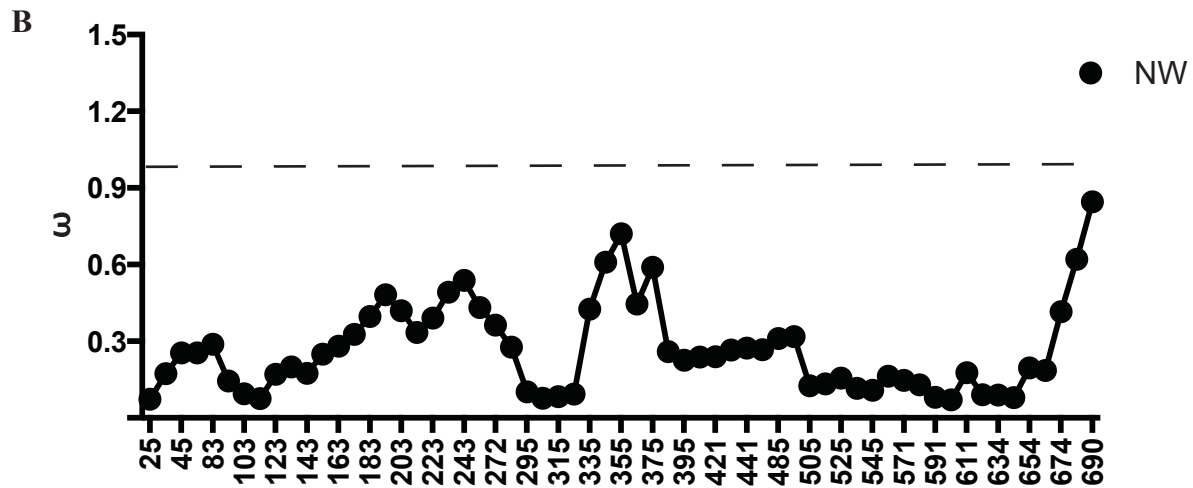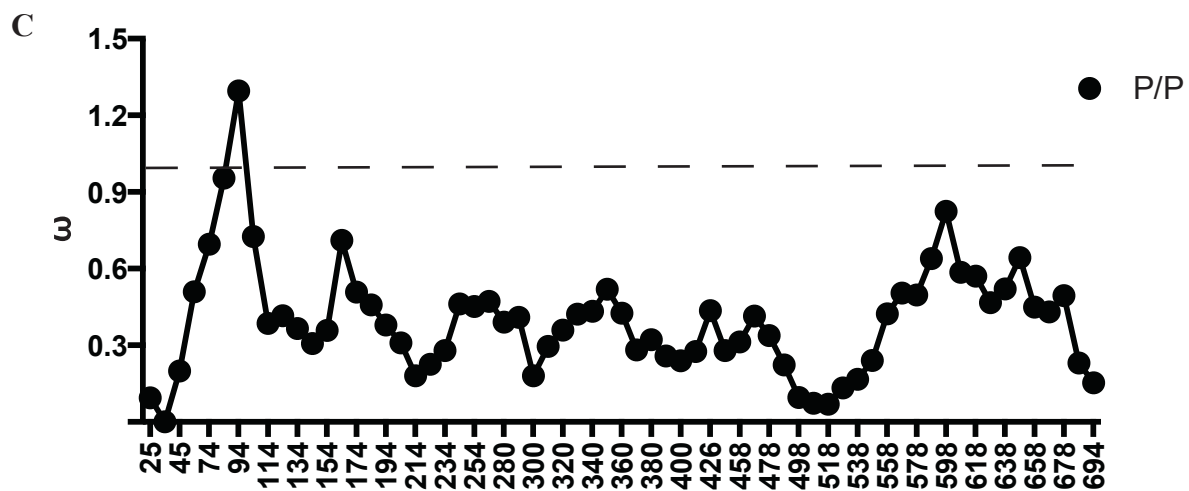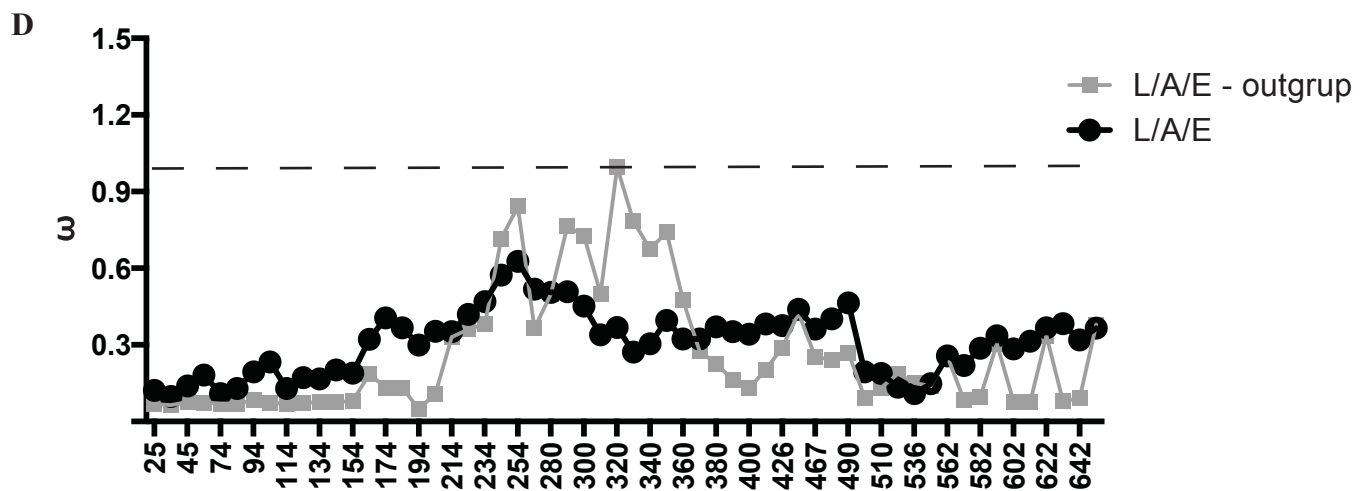

Supplement: S9 Fig — (A-D) Slide Window analyses of the ω values were performed for the D7 protein family in all sand flies (A) as well as only for the sequences belonging to the New World (B), Phlebotomus/Paraphlebotomus (C), Larroussius/Adlerius/Euphlebotomus (D), and the Larroussius/Adlerius/Euphlebotomus not belonging to the main clade (ω-L/A/E—outgrup). X-axes indicate nucleotide positions. (PDF) [file pntd.0004771.s009.pdf]

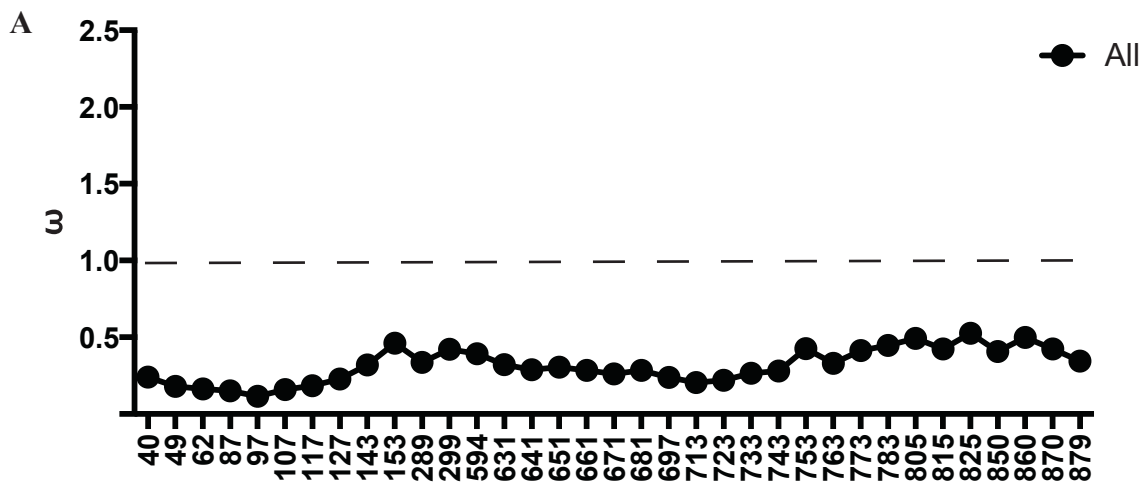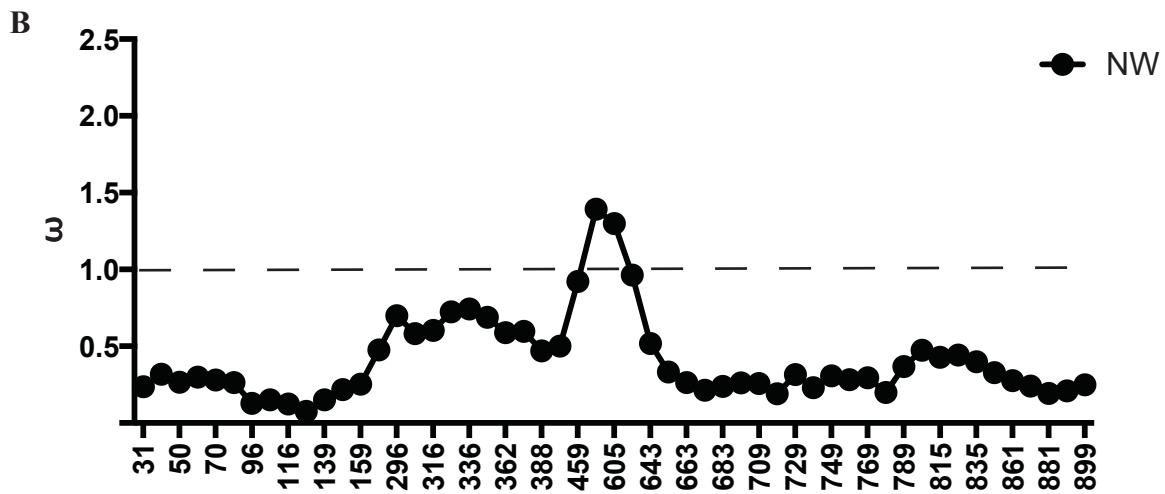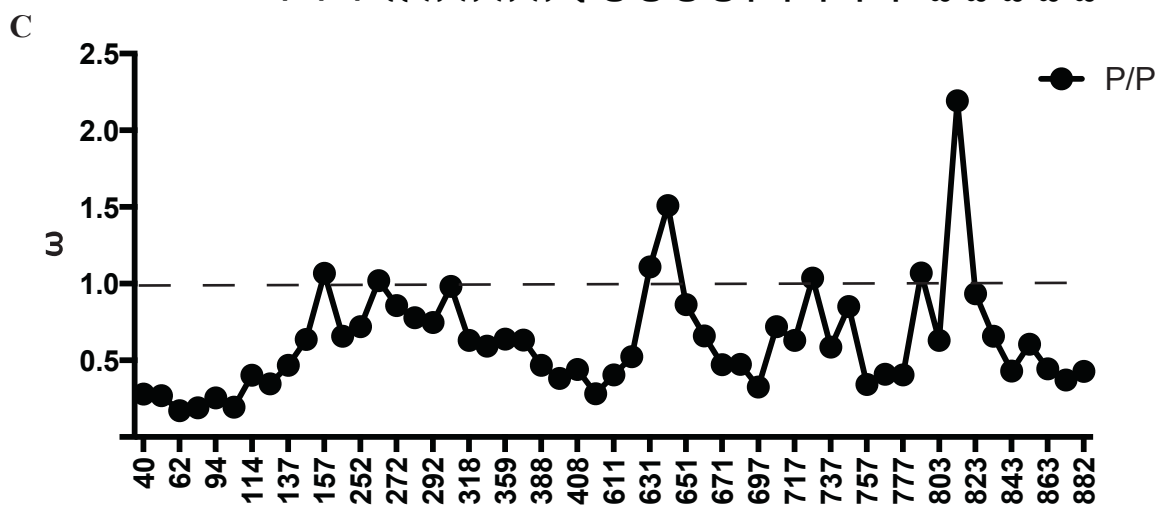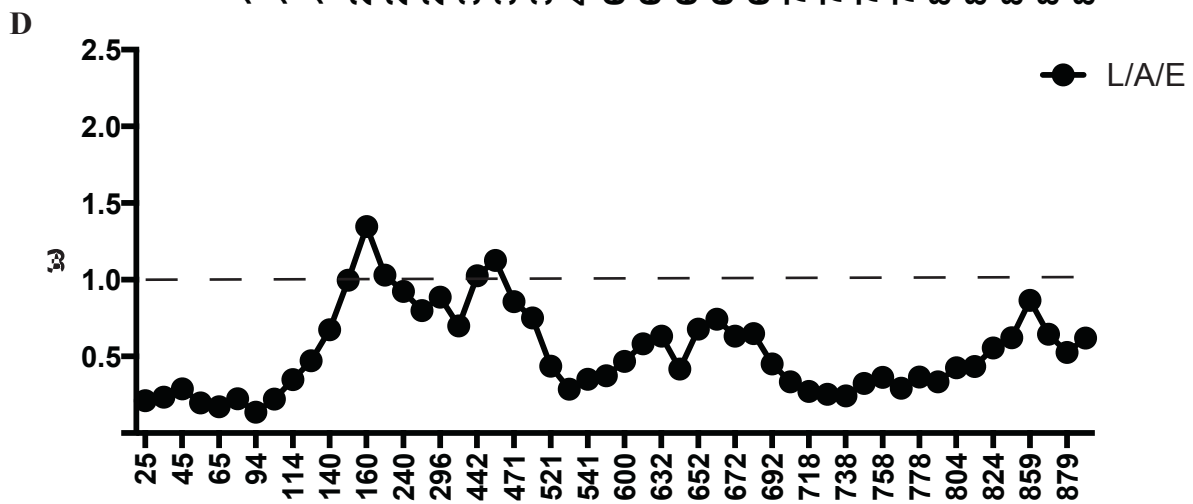

Supplement: S12 Fig — (A-D) Slide Window analyses of the ω values were performed for the Silk protein family in all sand flies (A) as well as only for the sequences belonging to the New World (B), Phlebotomus/Paraphlebotomus (C), and Larroussius/Adlerius/Euphlebotomus (D) clades. X-axes indicate nucleotide positions. (PDF) [file pntd.0004771.s012.pdf]

MW

Lofaxin

188

98

62

49

38

28

17

14

6

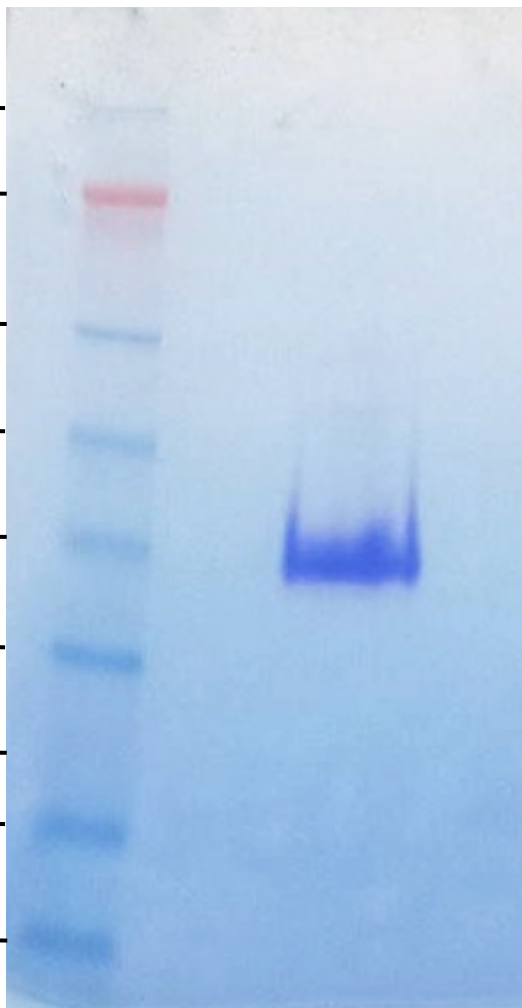

Supplement: S15 Fig — 5ug of rLofaxin protein was run in a 4–12 NuPage gel, colored with coomassie stain for 1h and destained to show the purified protein as a single band. (PDF) [file pntd.0004771.s015.pdf]

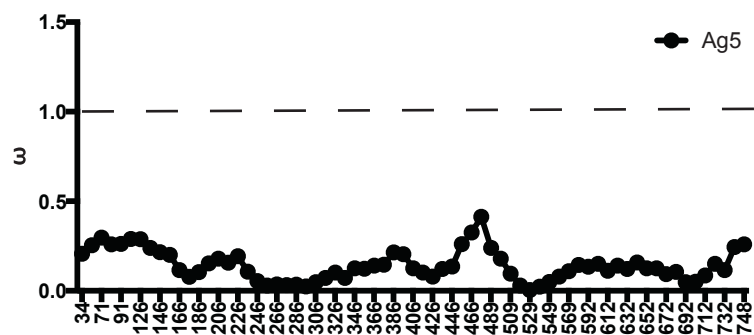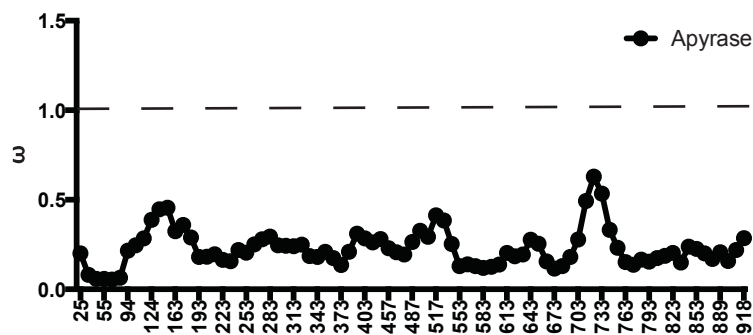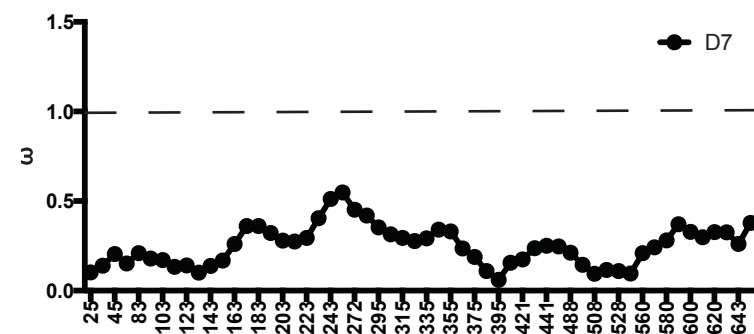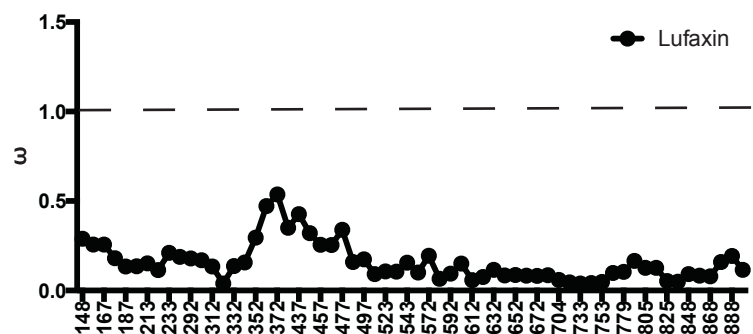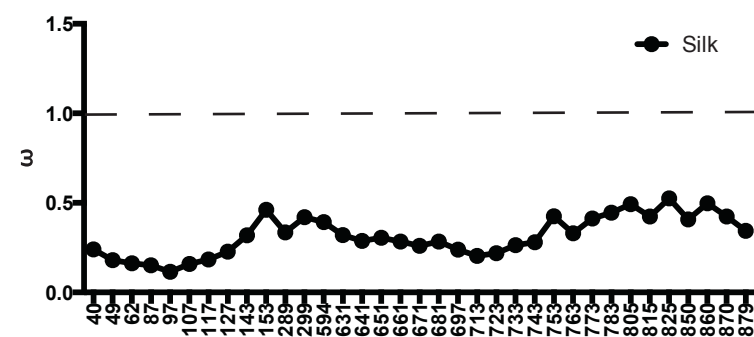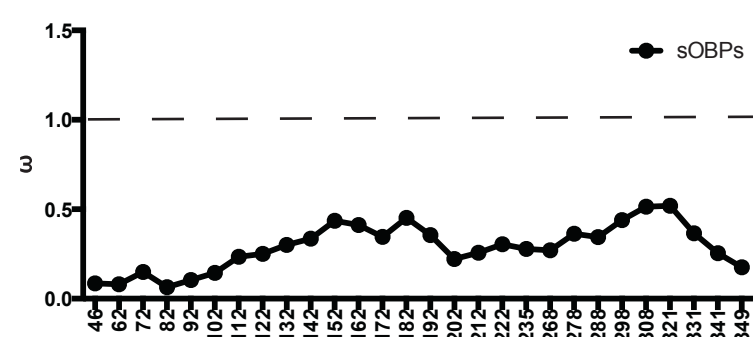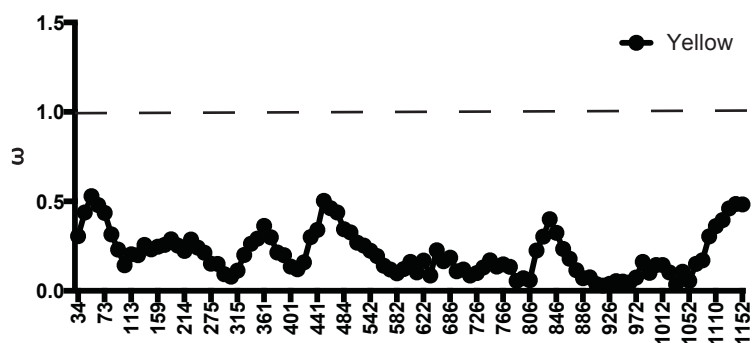

Supplement: S16 Fig — Slide Window analyses of the ω values were performed for the protein families shared between New World and Old World sand flies. Dashed bars indicate the threshold for positive selection. X-axes indicate nucleotide positions. (PDF) [file pntd.0004771.s016.pdf]

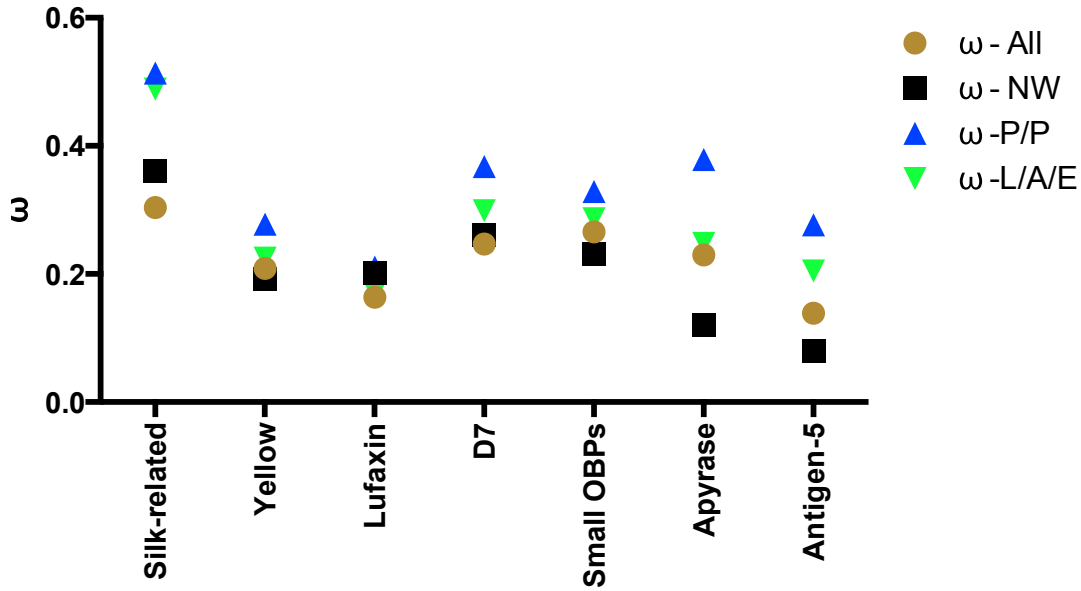

Supplement: S17 Fig — The mean rates of non-synonymous over the rate of synonymous replacements (ω) of protein families are depicted for the protein-encoding gene sequences of all sand flies (ω-All) as well as only for the sequences belonging to the New World (ω-NW), Phlebotomus/Paraphlebotomus (ω-P/P), and Larroussius/Adlerius/Euphlebotomus (ω-L/A/E) clades. (PDF) [file pntd.0004771.s017.pdf]

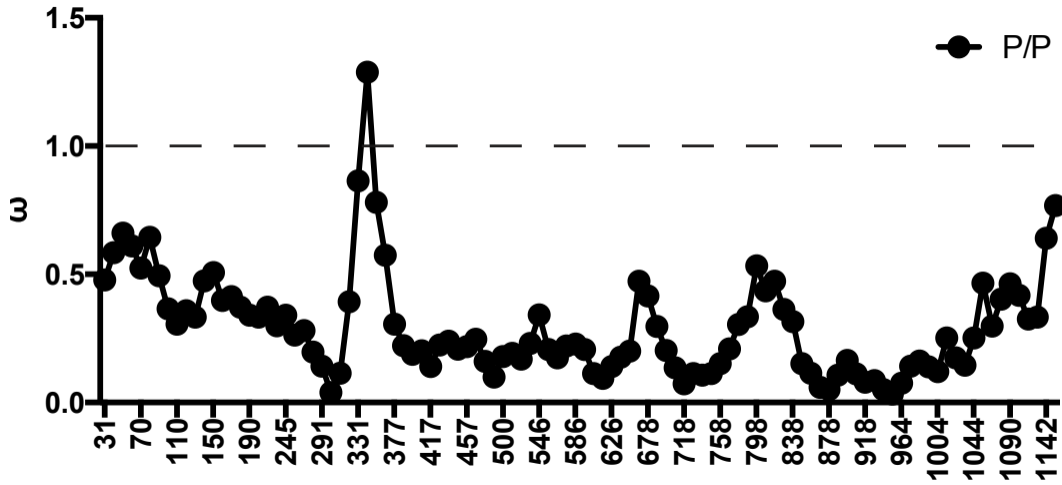

Supplement: S18 Fig — Slide Window analyses of the ω values were performed for the Yellow protein family for the sequences belonging to the Phlebotomus/Paraphlebotomus (ω-P/P) clades. Dashed bars indicate the threshold for positive selection. X-axes indicate nucleotide positions. (PDF) [file pntd.0004771.s018.pdf]
