# Supplementary material for: Molecular Diversity between Salivary Proteins from New World and Old World Sand Flies with Emphasis on Bichromomyia olmeca, the Sand Fly Vector of Leishmania mexicana in Mesoamerica
Source: PLoS Negl Trop Dis. 2016 Jul 13;10(7):e0004771. doi: 10.1371/journal.pntd.0004771 (PMC4943706; doi:10.1371/journal.pntd.0004771)
Supplement: S2 Fig — Multiple sequence alignment of the different SALO-like proteins (LolSALO) identified from the B. olmeca salivary gland transcriptome represented by (LolSALOa-f). Black background shading represents identical amino acids. Grey background shading represents similar amino acids. (PDF) [file pntd.0004771.s002.pdf]

```
LolSALOd      VGDIEQCKKGLVENTKTLVDMCNAKSLPPGPEPFSDEDLKPFFEEAGLKIAKECRDAEKG
LolSALOc      -ADEANCKKGLAESAQTLVVQCNNGVIEPGSDPFDVDKVGDLLDAEGIKKVKECIASEKG
LolSALOb      ----ANCEASIKEGLAGMTKACNDGKTPDDFSIISSTN-----NGPGAEITKACLDQEGA
LolSALOe      -----SCRTNLINNAVKLFNRCKQGHIKDEFFPFSETTFKN-FHAEGIKIAKKCLAVEAG
LolSALOf      ----MSCEDNLSEIFKTFESECNSGARADAFMEFNPSS-VG-LSAEGLAIAEKCIAEAKA
LolSALOa      ----MSCEDALKSSFQSLEGECNSGARADAFLEFNPSE-SG-LSAAGAAIAEKCIADAKA


LolSALOd      T---DCNKYTKISKCLSEKDLCSHSLKFPINV-----------
LolSALOc      T---DCNSFLKIGECLIGKDFCKHLK-----------------
LolSALOb      KSHGKCEKFGALEKCLIEKNLCSKIAA----------------
LolSALOe      SSHGKCKRFFDLKDCYMEENLCQYVKKIEKPKKSKNSKKKAKG
LolSALOf      NTHEKCKRFEDLYRCYLNTLLCSHLA-----------------
LolSALOa      KPHAKCKRFADLYNCYLTSGICAHIS-----------------
```
