# Supplementary material for: Molecular Diversity between Salivary Proteins from New World and Old World Sand Flies with Emphasis on Bichromomyia olmeca, the Sand Fly Vector of Leishmania mexicana in Mesoamerica
Source: PLoS Negl Trop Dis. 2016 Jul 13;10(7):e0004771. doi: 10.1371/journal.pntd.0004771 (PMC4943706; doi:10.1371/journal.pntd.0004771)

A

```
LolCTLc     -----------------YTISKEKKSWKDALKDCHRHRKTLVTIHSELEYKKLAEKFPNEVK-PADVPVWINGHNHNGQHGHS
LolCTLe     QRVNDKFVVKTLPTGKVIYISKTFMSWYGGLDYCNKFGLSLVSLNSQNENKQLSNVLQKVLP-RNEVHCWLGGYKFQDENGFN
LolCTLd     -----DLNAAEHLTGKTVLVSKVKKNWNDAKNFCKTKGYNLAMTKTKQEQYELSIIIE---KWTLEQHIWVGGHKDAK---DN
LolCTLb     -----DFHDKQEFKGKDIYISRIKKNWFDALDYCRKKKYTLLTIKSSEENANALASIEKLMEKKDEDHIWIGGFQFRPDK-PK
LolCTLa     -----DMHDKQVFKGKEIYISKVKKNWFDALDYCIKKDYSLLTIKSSEENENALAAIKKIMEKKDEDHIWIGGFQFQPDK-PK

LolCTLc     N-----------KHGNHTSYTNWAGGEPKMD--LAKSCVQI------KDGGKWYTADCNTPNYYACTKRRRRSRKHKKH
LolCTLe     SLRWVNGGR-------NITFTNWAKSEPSHG---NEHCLEMYYRQYRGDVATWNDRYCHDKHPFVCEKRSHGNAKRC—
LolCTLd     DFKWNDDRKLVAKEKTQNTFANWAKGKPGN--LKGEACMELEFNKSKNIYGEWNSADCSKQHIFVCEKRSA--------
LolCTLb     AYRWVSDLK-----KIVDVFTDWAPGEPNHGRNINEFCMEFFTKKSANIDMKWNDHVCSNEQYFICEKRKN--------
LolCTLa     TYRWVQNLR-----KIVDVYTDWAPGEPNHGRNINEFCMEFFTKKSANIDMKWNDHVCSNQQYFICEKRIK--------
```

Ligand binding surface

B

```
LolCTLc     -----------------YTVSKEKKSWKDALKDCHRHRKTLVTIHSELEYKKLAEKFPNEVKPA--------
tfiid       -----------------YTVSKDKKSYEDAVKDCRSKHQTLATIHSEDENKKLADEIPKEAQSTESKADNTT
LJL91       ----ADLTEKELSDGKKIFISKAELSWFDALDACTEKDLTLLTIKSARENEEVTKAVRAEVHLPDTK-----
LolCTLa     -----DMHDKQVFKGKEIYISKVKKNWFDALDYCIKKDYSLLTIKSSEENENALAAIKKIME--KKD-----
LolCTLb     -----DFHDKQEFKGKDIYISRIKKNWFDALDYCRKKKYTLLTIKSSEENANALASIEKLME--KKD-----
LolCTLd     -----DLNAAEHITGKTVLVSKVKKNWNDAKNFCKTKGYNLAMTKTKQEQYELSIIIEK-----WTL-----
Linb-63     -----NLNTGEHVTGKTIHVSKIKKSWKDARDYCSKNGYNLATIKSKKEQYEILIIITK-----WTM-----
Linb-48     -----ELNAEYELTGKSIYISKIKKNWFDAVDYCKNNGYELATIENEQEDSEIINALNK-----EKP-----
Linb-14     -----DFQKKQNLSGKTVFVSKMKKTWFDAKDYCLNKGYTLATVKSAKENGELTNVLKT-----MPV-----
Linb-15     -----DFNPEHKLTGKTILVSKIRKNWFDALDYCKNKGMTLATVRGRRENRELTNALRN-----SPV-----
LayS127     ----ENFVVRKLSSGKVIYVSKTFLSWYDALDFCNKYNLSLVSINSAKENTELAKTIRPIL---PEN-----
LolCTLe     QRVNDKFVVKTLPTGKVIYISKTFMSWYGGLDYCNKFGLSLVSLNSQNENKQLSNVLQKVL---PRN-----
Linb-22     ----QNLVVRKLPSGKTIYISKTYMSWYEGLDFCNRKGMSLVSIANDQENRQLSNVLQKIL---PEH-----
LJM06       -----EDYDKVKLTGRTVYISRSKAPWFTALDNCNRLRFTFAMIKSQKENEELTNALLSVI---KSD-----
LJS142      ----DQTLIEKELTGRTVYISKIKLNWNDAFDYCIRNGLTFAKIKSAEENTELSEKLKTVI---RTE-----
LJL18       ----EEELIERKLTGKTIYISTIKLPWFQALNHCVKNGYTMVSIKTFEENKELLKELKRVI---RTE-----

LolCTLc     -----------------------------------------------------------DVPVWIN------
tfiid       NNTANNTNNDTNNGTANGTANGTANGTANGTANNTNNDTNNGTNNGTNTDTNNGTSNGANSAVWINAVWNKH
LJL91       -----------------------------------------------------------KSHIWLGGIRYDQ
LolCTLa     -----------------------------------------------------------EDHIWIGGFQFQP
LolCTLb     -----------------------------------------------------------EDHIWIGGFQFRP
LolCTLd     -----------------------------------------------------------EQHIWVGGHKDAK
Linb-63     -----------------------------------------------------------AQHIWVGGYKNLN
Linb-48     -----------------------------------------------------------DSQIWIGGFRHPN
Linb-14     -----------------------------------------------------------ATHTWIGGIRHSQ
Linb-15     -----------------------------------------------------------ATHHWIGGIRHLN
LayS127     -----------------------------------------------------------EVHVWIGGYKFQD
LolCTLe     -----------------------------------------------------------EVHCWLGGYKFQD
Linb-22     -----------------------------------------------------------EVHCWIGGYKFQD
LJM06       -----------------------------------------------------------EENVWIGGLRHDL
LJS142      -----------------------------------------------------------EFQVWIGGIEHHQ
LJL18       -----------------------------------------------------------DTQVWIGGLKHHQ

LolCTLc     ------------------------------------GHNGQHGHSNKHGNHTSYTNWAGGEPKM-----DL
tfiid       QKFPHGSHGRHWSHGPHGPHGSHWSHGRHGPAHGPNGRHGLHGPHGPHGLHQPYANWAEGEPKS-----NG
LJL91       -------------------KDFRWISDG---------T-----TV----TKTVYINWYQGEPNG---GRYQ
LolCTLa     D---------------K-PKTYRWVQNL---------R-----KI-----VDVFTDWAPGEPNH---GRQI
LolCTLb     D---------------K-PKAYRWVSDL---------K-----KI-----VDVFTDWAPGEPNH---GRNI
LolCTLd     ------------------DNDFKWNDDG---------K-----LVAKEKTQNTFANWAKGKPGN----PKG
Linb-63     ------------------GDHLIWINDG---------K-----PIPKERTQTTFSNWASGKPNS---NKKD
Linb-48     ------------------EHPFRWVINF---------K-----KI----DSTVYPNWQPKQPNY---SKND
Linb-14     ------------------ENNFRWEGTQ---------K-----QI----DSTVYTNWEQGEPNN---GRYI
Linb-15     ------------------ENFFRWIHNN---------N-----KI----ETTGYTNWQPGEPNF---GRGI
LayS127     E---------------NGENSQRWVNGG---------R-----NV-------TYTNWSSGEPNN----ANG
LolCTLe     E---------------NGFNSLRWVNGG---------R-----NI-------TFTNWAKSEPSH------G
Linb-22     E---------------NGQNSMRWINGG---------R-----KV-------DYTNFAGGEPNN------D
```

```
LJM06      ------------------DDYFRWISFG---------T-----ALSK----TSYTNWAPKEPTGRPHRTQN
LJS142     ------------------DSSFRWVSDS---------Q-----PITNK-LGYKYTNWNTGEPTN----YQN
LJL18      ------------------FANFRWVSDG---------S-----HVATA-SG--YTNWAPGEPAD---SFYY

LolCTLc    AKSCVQIK------DGGKWYTADCNTPNYYACTKRRRRSRKHKKH----------------------
tfiid      TNSCVEIK------NDGKWYTSDCKTENYYACTTEGRRGSWRRRQRHHRRRHWRH------------
LJL91      KEFCMELYFKTP---AGQWNDDICTAKHHFICQEKK-------------------------------
LolCTLa    NEFCMEIFTKKSANIDMKWNDHVCSKEQYFICEKRIK------------------------------
LolCTLb    NEFCMEFFTKKSANIDMKWNDHVCSNEQYFICEKRKN------------------------------
LolCTLd    -EACMELEFNKSKNIYGEWNSADCSKQHIFVCEKRSA------------------------------
Linb-63    -EMCMELDFSKAKDSHGEWNVNDCAKEHIFVCEKRSG------------------------------
Linb-48    -ELCLEYWNYPAKSNIFKWNDRKCALEQIFICENRNTKPKKNKSFME---YNYFPDSSVVVNSEING
Linb-14    -EFCMEYWNDVTKNVEWKWNDNDCKQEQIFVCEKRGIKKSTQHLTRK---M----------------
Linb-15    -ELCMEYWNDPTKNIEWKWNDNDCKQEQIFVCEKRD-------------------------------
LayS127    REYCLEIYYRQYRGATDKWNDRFCTDKHPFVCERKC-------------------------------
LolCTLe    NEHCLEMYYRQYRGDVATWNDRYCHDKHPFVCEKRSHGNAKRC------------------------
Linb-22    KEYCLEMYYRQYRGDTARWNDRYCTDRHPFVCEKKC-------------------------------
LJM06      DEFCMQMSF----KDGGKWSDNTCWRKRLYVCEKRD-------------------------------
LJS142     NEYCLEILFR---KEDGKWNDFPCSARHHFVCEKRTK------------------------------
LJL18      DQFCMAMLFR---KDGAPWDDLNCWVKNLFVCEKRDD------------------------------
            |__________________________________|
                  Ligand binding surface
```

C

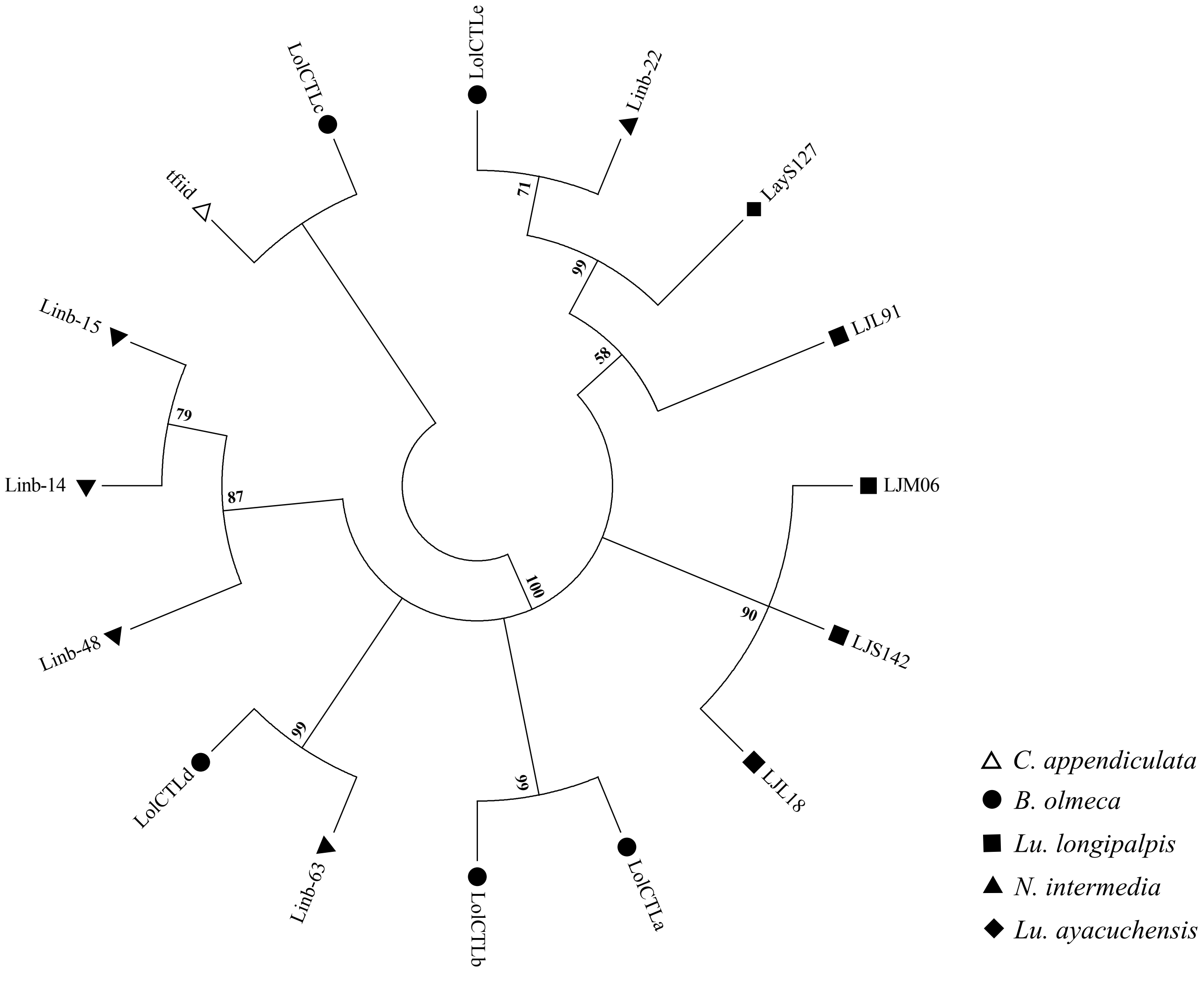

Supplement: S4 Fig — (A) Multiple sequence alignment of the different C-type lectin-like proteins (LolCTLa-e) identified from the B. olmeca salivary gland transcriptome. Black background shading represents identical amino acids. Grey background shading represents similar amino acids. (B) Multiple sequence alignment of the sand fly C-type lectin protein families. Multiple sequence alignment of the different C-type lectin-like proteins (LolCTLa-e) identified from the B. olmeca salivary gland transcriptome with homologs identified from Lu. longipalpis (LJL18, LJL91, LJS142, LJM06), Lu. ayacuchensis (LayS127), N. intermedia (Linb-14, 15, 22, 48 and 63) sand flies and C. appendiculata (tfiid) frog-biting fly. Black background shading represents identical amino acids. Grey background shading represents similar amino acids. (C). Phylogenetic tree depicts multiple distinct branches containing C-type lectin orthologs in New World sand flies as well as multiple paralogs are noticed within the branches. The evolutionary history was inferred based on the Le Gascuel 2008 model [64]. Sand fly species are indicated by the different symbols in the legend on the right. (PDF) [file pntd.0004771.s004.pdf]
