# Supplementary material for: Molecular Diversity between Salivary Proteins from New World and Old World Sand Flies with Emphasis on Bichromomyia olmeca, the Sand Fly Vector of Leishmania mexicana in Mesoamerica
Source: PLoS Negl Trop Dis. 2016 Jul 13;10(7):e0004771. doi: 10.1371/journal.pntd.0004771 (PMC4943706; doi:10.1371/journal.pntd.0004771)

A

```
Linb-45     ----NFNFLPKDRDDCFVSLMTPAPGNKDCLED---IDGKDVQNGKKTFIKCTDSK--GN
Lol14.2a    LSPQRSEVLPKDREDCIVSLMTPMS--ETCKKD---IDDKPINNGKETYIKCKTSENAGS
Lol14.2b    -IDLHLQIMPKDPNDCVINENYLLFLDTPCDSD---TTNTPVKNGKKIYIVCTNSE--GD
Lol14.2c    -SDLHLQIMPKDPNDCVIDENYLLLLDTPCDSD---TTNTPVMNGKKIYIVCDNSE--GD
LJM114      -------FLPSDPSICVKNLVLDTG--RTCEESEYFPDIKNVKNGKRVYIVCTDSD--AV

Linb-45     EYTFYDCFDINLFVSHRSP-----DPEPITYTKEAQVSFALVQKHIASKYT---------
Lol14.2a    EYTFYDCFNVNVFVSPPSV-----RPEPITYSAEAQVSYALVKKHYASQVAAGLV-----
Lol14.2b    SFKFYECFEASLLG-----------SNALTVNKVMELYRKRNEEVRV-------------
Lol14.2c    HFKFYECFDSSFLG-----------P-GVSFPVSTKLLKKLLEQLLR-------------
LJM114      DYKFYICFDMNRLSGPPYPEEEILRESTVTYAQIYELMTTETTETKKPKKKPKNSKTDPD

Linb-45     ----------------
Lol14.2a    ----------------
Lol14.2b    ----------------
Lol14.2c    ----------------
LJM114      PPAIRPGFSFRNSISV
```

B

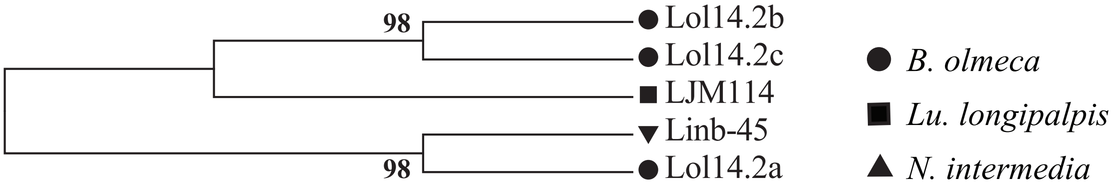

Supplement: S5 Fig — (A) Multiple sequence alignment of the different 14.2-like proteins (Lol14.2a-c) identified from the B. olmeca salivary gland transcriptome with homologs identified from Lu. longipalpis (LJM114) and N. intermedia (Linb-45) sand flies. Black background shading represents identical amino acids. Grey background shading represents similar amino acids. Gene alternative splicing was shown in the C-terminus part of the Lol14.2b and Lol14.2c sequences. (B) The phylogenetic tree shows two distinct branches, to which orthologs between B. olmeca and N. intermedia and B. olmeca and Lu. longipalpis belong. The evolutionary history was inferred based on the Whelan And Goldman model [62]. Sand fly species are indicated by the different symbols in the legend on the right. (PDF) [file pntd.0004771.s005.pdf]
