# Supplementary material for: Molecular Diversity between Salivary Proteins from New World and Old World Sand Flies with Emphasis on Bichromomyia olmeca, the Sand Fly Vector of Leishmania mexicana in Mesoamerica
Source: PLoS Negl Trop Dis. 2016 Jul 13;10(7):e0004771. doi: 10.1371/journal.pntd.0004771 (PMC4943706; doi:10.1371/journal.pntd.0004771)
Supplement: S6 Fig — Multiple sequence alignment of the different ML-domain-like proteins (LolMLa-d) identified from the B. olmeca salivary gland transcriptome. Black background shading represents identical amino acids. Grey background shading represents similar amino acids. (PDF) [file pntd.0004771.s006.pdf]

```
LolMLc          -GAWYRMYWEYDCPGYKRPLDYSIVECPPYT----TCSKISKVGDFAVMHVNYTFKVENG
LolMLa          FWWWWNDYGTTNCVSTSAPKSISIDECSSTSVSNAIVCRTV-KGNQGVMNINFEFKIDR-
LolMLb          --SEEDKYFLRSCEGVKMPIAFSIKQCPKSK--DYICNGIL-KGAIALIDVELQFKIDK-
LolMLd          -----QEFKLFKCPDIALPNKVKIAQCPSKK----PCSNII-NGKDATITGIFEFNINH-

LolMLc          PYAKLPISGQLMRYPSGK--VEKFFLG-DDACGKGKQYLLKEKCPLQNGIHWIQVKLIMR
LolMLa          ELKSLPIYANYKVRRGYEFQTTKFSLP-EDVCKNQALYKLKEPCPLKPGSYKITFPLKIP
LolMLb          EFTKLPISADVIR-NGKT---EVIPLP-NNACK----GFYKEKCPLKPGKHTITFPLLIN
LolMLd          KTSKLPITANVKRANGKS---EIIALPFGDACN----SVVKTKCPLKPGAHKIKLPLRVK

LolMLc          NVKLHEFIKVALRVNDPS---DKPLACVYAGAYANGILTYY
LolMLa          NIKLKEWVYIGLGVFDDKADTEYPFVCTYVEIFTRA-----
LolMLb          NVKKDDLLHIGVGIGDPE--TKKNFACAYFELKAK------
LolMLd          DVKRGEKLTVSVTIRDNK--N-KPIVCAAVELTAK------
```
