# Supplementary material for: Molecular Diversity between Salivary Proteins from New World and Old World Sand Flies with Emphasis on Bichromomyia olmeca, the Sand Fly Vector of Leishmania mexicana in Mesoamerica
Source: PLoS Negl Trop Dis. 2016 Jul 13;10(7):e0004771. doi: 10.1371/journal.pntd.0004771 (PMC4943706; doi:10.1371/journal.pntd.0004771)

A

```
Linb-59     -NHPQQYCIDKLAETEESCIQHCRFSYYGFTNDKFQITKKHIEKFRDILLEFNAVPKSKK
LolSOBPc    SNNPANYCIKKLAKTEEACIQHCKYRHYGFTNNNFKITKKHIENLRTVLLEFNAVPISQK
Linb-28     -ESPVRKCVREKARTQLICMTQCKYNYYGFTDEDSNITEKHMENFRDVLVKYGAVPSSDQ
LolSOBPb    -ESPQRKCLRELEGTQVNCITYCTYNHYGFTNKNYKITKKHIEKFRDILIEYKAIPLSDN
LuloOBP     -EHPEEKCIRELARTDENCILHCTYSYYGFVDKNFRIAKKHVQKFKKILVTFDAVPKKEK
LayS58      -ENPEEKCIRELARTDEVCILHCSYSYYGFTDENYRITKKHIETFRDVLISYNAVPGNEK
LayS66      -EHPERKCIRELARTDEVCILHCSYSYYGFTDENYRITKKHIETFRDVLISYNAVPGNEK
LolSOBPa    -ETPHQKCNREWGRTQEGCITHCTYKHYGFSDNNYRITKKHIEKLRDILIEYKAVPLSDK
Linb-8      -GHPEKKCIQELGRTQESCITHCQYQHYGFTDENYRITKKHMEKFRDVLIEYKSVPLSDK
Linb-7      -EHPEKKCIQELGKTQSSCILHCEYNHYGFTDENYRITKKHMEKFRDVLIEYRSVPLSDK

Linb-59     NQLFNHIKKCADKVNSLKSKDKSEKCMKILTYSRCVADGKTVSEHNYVTAIIAHDKRINV
LolSOBPc    NQLYDHLKKCADKANALKPKTRREKCYKIITYSRCAYGGKVVSEHKYVKALIEHDKLINV
Linb-28     AKIFDHIKACGQQANAKNPQNTEEKCKKLTKYYKCIVDNKTLTFSKYVQAVIKHDKTLNV
LolSOBPb    SKITNHIKACAKKAKSKVSNTTEEKCEKLVEYYKCVVDGKTLSWNLYAKVIDKYDKTFNV
LuloOBP     KKLLEHIEACADSANADQPQTKDEKCTKINKYYRCVVDGKILPWNSYADAIIKFDKTLNV
LayS58      NKLFDHIKACADAANATKPKSHNDKCYKIIHYYRCVVDGKVLSWNSYAAAIIKYDKTKNV
LayS66      NKLFDHIKACADAANATKPKSHNDKCYKIIHYYRCVVDGKILSWNSYAAAIIKYDKTKNV
LolSOBPa    SKITGHIRACADRANAKKPKSTEDKCKKIIHYYRCVVDGKTLTWNRYAHAMIPYEKTFNL
Linb-8      SKLFGHIRACGDKVNAKKPKSTQDKCMKIIEYYRCVVDGKLLTWNRYANAMIQYDKTINV
Linb-7      SKLFGHIRACGDRANAKKPKSTEDKCQKINDYHRCIVDEKFLTFNRYYLAVNKYDKTINV
```

B

```
PPTSP14     FEHPEAFCIKKH--KDTDFECILHCKFKYYNFVDDKYNIKDYHIRNLADFLINYNVVPANKRRNV
PagSP02     -ATPAKKCREGD--LRKTEVCILHCEYSHYGFAGNNFKIDEKHTKKLTDILIQYGGVAKNKAKDI
LolSOBPc    SNNPANYCIKKL--AKTEEACIQHCKYRHYGFTNNNFKITKKHIENLRTVLLEFNAVPISQKNQL
Linb-59     -NHPQQYCIDKL--AETEESCIQHCRFSYYGFTNDKFQITKKHIEKFRDILLEFNAVPKSKKNQL
Linb-28     -ESPVRKCVREK--ARTQLICMTQCKYNYYGFTDEDSNITEKHMENFRDVLVKYGAVPSSDQAKI
LolSOBPb    -ESPQRKCLREL--EGTQVNCITYCTYNHYGFTNKNYKITKKHIEKFRDILIEYKAIPLSDNSKI
LuloOBP     -EHPEEKCIREL--ARTDENCILHCTYSYYGFVDKNFRIAKKHVQKFKKILVTFDAVPKKEKKKL
LayS58      -ENPEEKCIREL--ARTDEVCILHCSYSYYGFTDENYRITKKHIETFRDVLISYNAVPGNEKNKL
LayS66      -EHPERKCIREL--ARTDEVCILHCSYSYYGFTDENYRITKKHIETFRDVLISYNAVPGNEKNKL
LolSOBPa    -ETPHQKCNREW--GRTQEGCITHCTYKHYGFSDNNYRITKKHIEKLRDILIEYKAVPLSDKSKI
Linb-8      -GHPEKKCIQEL--GRTQESCITHCQYQHYGFTDENYRITKKHMEKFRDVLIEYKSVPLSDKSKL
Linb-7      -EHPEKKCIQEL--GKTQSSCILHCEYNHYGFTDENYRITKKHMEKFRDVLIEYRSVPLSDKSKL
PduM99      -AHPFEKCKEDSKAGKFGEECILHCQYKIYGFTNKKYEISSYHIEKFYKVLSKGNIVQKNDKNKL
PpeSP11     -EPPSKKCRSGL---VKDEECILHCEYKYYGFTDDNFELDSDLRGHFRTAMRKHGAIRIDQERQL
PtSP18      -ERPSRKCRSGI---VKEEECILHCEYQYYGFTDNKFRLNADQRGNFRFAMMDYGAIGMDQEDQM
PorMSP74    -ERPSRKCRSGL---VKEEECILHCEYKYYGFTDDKFELDADQRGDFRNAMRKYGAIRIDQERQL
PorASP61    -ERPSRKCRSGL---VKEEECILHCEYKYYGFTDDKFELDADQRGDFRNAMRKYGAIRIDQERQL
ParSP06     -ERPSQKCRREL---KTEEECILHCEYKHYRFTDDQFRLNADQRGDFRNIMRRYGAIRVDQESQL
PabSP45     -ERPSQKCRREL---KKEEECILHCEYKHYHFTDDQFGLDSDQRGDFRNAMRRYGAITVNQERQL
PsSP15      -ETPENKCIAKHRANNLKETCIPQCKYEYYGFVGPDYNITYQHIRTFSNTLIKYNAIDVSKKHEL
PsSP11      -GNPSKKCREDYRAKKLDESCILHCEYKAYGFSNDKYDIKKKQIDKFVEVLINAKAVDSSNRTKL
PPTSP12     -LNPSRKCRLDYKDKVISESCILHCEYKAYGFANDKYDIKRKQIDQFVDVLINGKAVASDKRQKL

PPTSP14     EAHLKSCVTKSIK----KHRTPSCDSIFSYYTCISDE-KLIYFNDYDNAIRRYDQTLTVVTRKN
PagSP02     RRHLRNCANEALARS-ALNKDQKCTRVIDYYRCAVKT-DLFSYTSYATAVIKYDKTINV-----
LolSOBPc    YDHLKKCADKANALK-PKTRREKCYKIITYSRCAYGG-KVVSEHKYVKALIEHDKLINV-----
Linb-59     FNHIKKCADKVNSLK-SKDKSEKCMKILTYSRCVADG-KTVSEHNYVTAIIAHDKRINV-----
Linb-28     FDHIKACGQQANAKN-PQNTEEKCKKLTKYYKCIVDN-KTLTFSKYVQAVIKHDKTLNV-----
LolSOBPb    TNHIKACAKKAKSKV-SNTTEEKCEKLVEYYKCVVDG-KTLSWNLYAKVIDKYDKTFNV-----
LuloOBP     LEHIEACADSANADQ-PQTKDEKCTKINKYYRCVVDG-KILPWNSYADAIIKFDKTLNV-----
LayS58      FDHIKACADAANATK-PKSHNDKCYKIIHYYRCVVDG-KVLSWNSYAAAIIKYDKTKNV-----
LayS66      FDHIKACADAANATK-PKSHNDKCYKIIHYYRCVVDG-KILSWNSYAAAIIKYDKTKNV-----
LolSOBPa    TGHIRACADRANAKK-PKSTEDKCKKIIHYYRCVVDG-KTLTWNRYAHAMIPYEKTFNL-----
```

```
Linb-8       FGHIRACGDKVNAKK-PKSTQDKCMKIIEYYRCVVDG-KLLTWNRYANAMIQYDKTINV-----
Linb-7       FGHIRACGDRANAKK-PKSTEDKCQKINDYHRCIVDE-KFLTFNRYYLAVNKYDKTINV-----
PduM99       RKLLKDCADQAERDP----TSKDCRKINNYYRCIVNN-DLVSYNKYAEIIIAHDKTFNV-----
PpeSP11      DKHLKKCAQEAK-------KSEKCRKIIQYYRCAVNN-KLFQYNAYAKAIIALDKTINV-----
PtSP18       DEHLKKCANEAKKAP-VKSKSDKCRKIIQYYRCAVDN-KLFQYNAYAKAIIALDKTINV-----
PorMSP74     DKHLKKCANEAKKAP-VKSKSDKCRKIIQYYRCAVDN-KLFQYNAYAKAIIALDKTINV-----
PorASP61     DKHLKKCASEAKKAP-VKSKSDKCRKIIQYYRCAVDN-KLFQYNAYANAIIALDKTINV-----
ParSP06      DKHLKKCANKVAKTP-ATSRKDKCRKISRYYHCAVDN-KLFKYNDYANAIIKYDKTINV-----
PabSP45      DKHLQKCAKKVQKTP-AKTRSEKCIKITQYYRCAIDN-KLFRYNDYAQAIIKLDKSMNV-----
PsSP15       RKLMQKCEKRVKNQARNDSHWLNCRTTIEYYRCIVAD-PMINYRKFDKAIIEYDKTINV-----
PsSP11       DNLLRKCANQARSK---HSNSLNCYTTIDYYRCIVNDDSLINYRKFVGAIMAYDKTINI-----
PPTSP12      ENLLRGCANKARDK---N-PKLGCHTSIDYYRCIVADQKLINYSKFVGAIIAYDKKINLN----
```

C

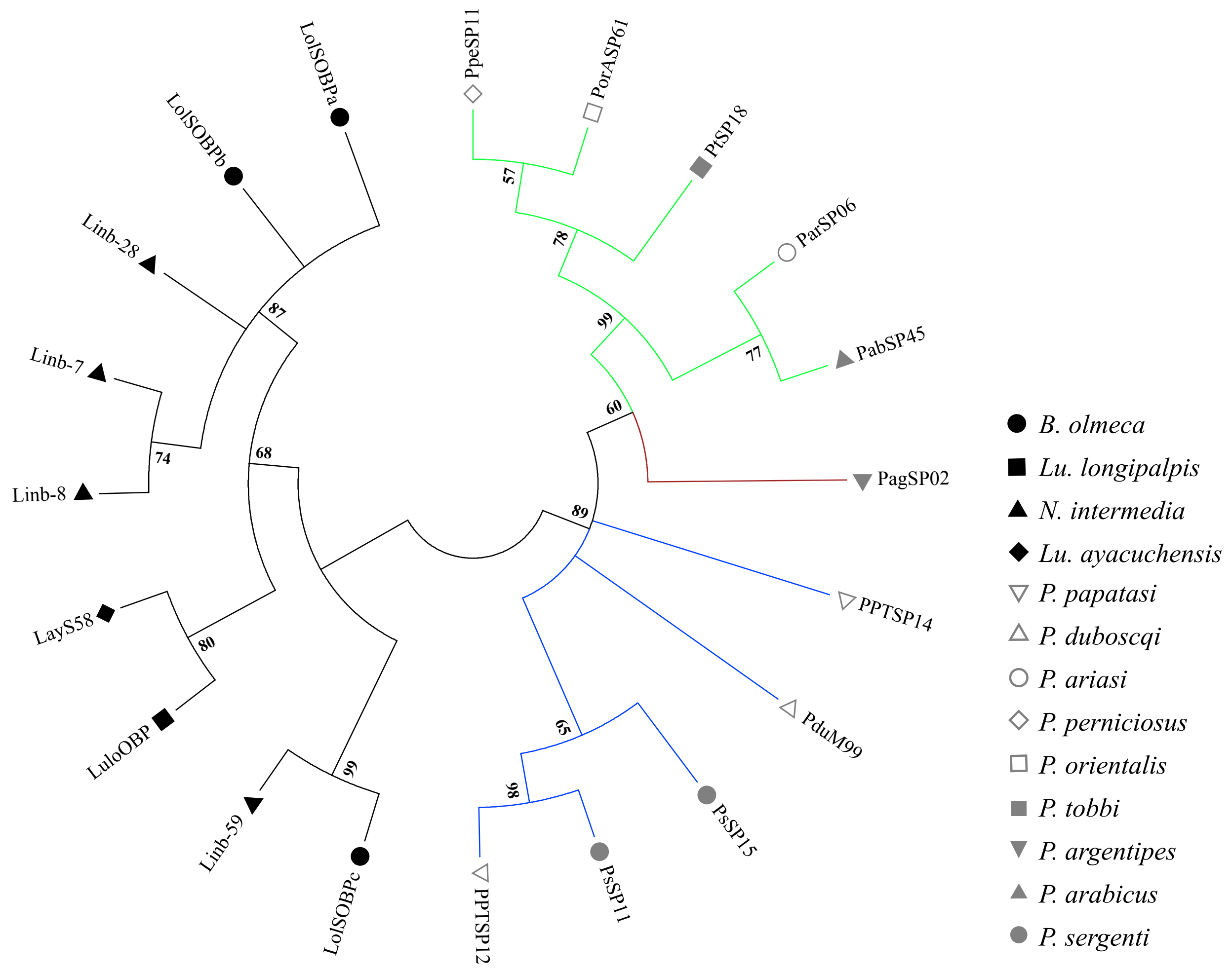

Supplement: S7 Fig — (A) Multiple sequence alignment of the different small OBP proteins (LolSOBPa-c) identified from the B. olmeca salivary gland transcriptome with homologs identified from Lu. longipalpis (LuloOBP), Lu. ayacuchensis (LayS58) and N. intermedia (Linb-7, 8, 28 and 59) sand flies. Black background shading represents identical amino acids. Grey background shading represents similar amino acids. (B) Multiple sequence alignment of the different small OBP proteins (LolSOBPa-c) identified from the B. olmeca salivary gland transcriptome with homologs identified from New World and Old World sand flies. Black background shading represents identical amino acids. Grey background shading represents similar amino acids. (C) The phylogenetic analysis shows the split of New World and Old World sand fly proteins in distinct clades. Although multiple paralogs are noticed for the New World sand fly sequences, only P. sergenti displays a paralog amongst the Old world sand fly Small OBPs. The evolutionary history was inferred based on the Whelan And Goldman model [62]. Sand fly species are indicated by different symbols. Tree branches were color-coded so as to represent specific taxa: Green color represents the Larroussius and Adlerius subgenera; Red color indicates the Euphlebotomus subgenus; Blue color points to proteins of the Phlebotomus and Paraphlebotomus subgenera; and Black color indicates the proteins belonging to New World sand flies. (PDF) [file pntd.0004771.s007.pdf]
