# Supplementary material for: Molecular Diversity between Salivary Proteins from New World and Old World Sand Flies with Emphasis on Bichromomyia olmeca, the Sand Fly Vector of Leishmania mexicana in Mesoamerica
Source: PLoS Negl Trop Dis. 2016 Jul 13;10(7):e0004771. doi: 10.1371/journal.pntd.0004771 (PMC4943706; doi:10.1371/journal.pntd.0004771)
Supplement: S8 Fig — (A) Multiple sequence alignment of the D7-like proteins (LolD7) identified from the B. olmeca salivary gland transcriptome with homologs identified from Lu. longipalpis (LJL13), Lu. ayacuchensis (LayS101) and N. intermedia (Linb-42) New World species and P. arabicus (PabSP20, 54, 59 and 84), P. tobbi (PtSP42, 54, 57 and 58), P. papatasi (PPTSP28a and 28c), P. ariasi (ParSP12 and 16), P. perniciosus (PpeSP04 and 04B), P. orientalis (PorMSP28, 38 and 43) and P. sergenti (PsSP4 and 7) Old World species. Black background shading represents identical amino acids. Grey background shading represents similar amino acids. * Indicates the essential amino acids for leukotriene binding activity in the mosquito D7 protein. (B) The phylogeny depicts a large clade encompassing D7 proteins of sand flies belonging to the Larrossius sub-genus, out-grouped by other clades belonging to either New World sand fly D7 proteins or their counterparts of members of the Phlebotomus and Paraphlebotomus sub-genera. All such sequences were out-grouped my D7 proteins belonging to sand flies of the Larrossius sub-genus. The evolutionary history was inferred according to the Jones et al. w/freq. model [63]. Sand fly species are indicated by different symbols. Tree branches were color-coded so as to represent specific taxa: Green color represents the Larroussius and Adlerius subgenera; Red color indicates the Euphlebotomus subgenus; Blue color points to proteins of the Phlebotomus and Paraphlebotomus subgenera; and Black color indicates the proteins belonging to New World sand flies. (PDF) [file pntd.0004771.s008.pdf]

A

```
                             *                               *
PabSP59     LQFPRNPDQMRWATKTCLREFRDAPPSLIKEWNEWDLPSSNLTYCFVKCNWIYAGIYNEK
PtSP42      LQFPRNPDQTRWAEKTCLREFSRAPPSLLKKWQELNFPNTNLTHCFVKCVSTYLGVYNET
PabSP84     FKYPRTAEQSLWAFRACQSEIL-PDLNLLKQWNNWDLPDNNKTHCYLKCSLMYLGAYNTK
LolD7       WQDVRNADQSLWAFRTCQRRSS--NVDLVKKWMAYVLPDDKETHCYVMCAWSLLGAYSEE
LJL13       WQDVRNADQTLWAYRSCQKNPE--DKDHVPQWRKFELPDDEKTHCYVKCVWTRLGAYNEN
Linb-42     WQDVRNADQSLWAYKSCMRDPK--DVDLVPKWRKFIFPNDERTQCYVKCLWTRLGAYNQD
PPTSP28a    WKYPRNADQTLWAFRTCQRRES--DNNILKKWYTWELPNNKETHCYVKCVWIHLGLYSKS
PPTSP28c    WKYPRNADQTLWAFRTCQRRES--DNNILKKWYTWELPNDEKTHCYVKCVWIHLGLYSKS
PsSP7       LQYPRNADQTLWAFRTCQRRES--DSSLLQNWFQWKLPNNEATHCYVQCSWIHLGLYNKK
PsSP4       WQYPRNADQALWAFRTCQRRES--DASLVLKWYQWQLPNNAATHCYVKCGWIHLGMYNRK
PabSP54     WQYPRNADQTLWAFRSCQKEKY--DQELVNKWKKFELPDHPQTHCFIKCVWTHLGMYDEK
ParSP16     WKYPRNADQTLWAWRSCQKGNY--DPELVKKWMAFEIPDDEVTHCYIKCVWTHLGMYDET
PpeSP04     WQYPRNADQTLWAWRSCQKEHIGDDQALLKKWLKFEIPDDKVTHCFIKCTWIHLGMYDEK
PorMSP28    WRYPRNADQTLWAWRSCQKEHIGGDQALLQKWLKFEIPDDKVTHCFIKCIWIHLGMYDEE
PtSP54      WQYPRNADQTLWAWRSCHKEHSGGDQALLQKWMKFEIPDDKVTHCLVKCIWIHLGMYDAK
LayS101     WQYPRNADQTLWAFKTCQREAK--DQKFLEKWKKWELPASDETYCYVKCVWKNLGSYNDA
PtSP57      WKYPRNADQTLWAFRSCQREGK--NPDLVKKWMNWQLPNDPETHCYVKCVWTNLGSYDDK
PpeSP04B    WQYPRNADQTLWAFRSCQREGK--NPDLVKKWMNWELPNDRETHCYVKCVWIHLGSYNHN
PtSP58      WKYPRNADQTLWAFRSCQREGK--NPDLVKKWMNWELPNNPETHCYVQCVWTNLGSYDDK
PorMSP43    WKYPRNADQTLWAFRSCQREGK--NPDLVKKWMNWELPNDPETHCYVHCVWNNLGSYDDD
PorMSP38    WKYPRNADQTLWAFRSCQREGK--NPDLVKKWMNWELPNDRETHCYVKCVWTNLGSYDDK
ParSP12     WKYPRNADQTLWAYRTCQREGK--DPALVSKWMNWVLPDDPETHCYVKCVWTNLGSYDDN
PabSP20     WQYPRNADQTLWAFRACQREGK--NPALVRKWMNWELPDDTETHCYVKCVWTYLGSYNED

                                               *
PabSP59     TNEFNVDAIRTQFTSQGLLPPKGLENLQRRPAKTSCKDIYRMSIGIIKKYKSDFIKAFYG
PtSP42      TKSFNVDGIRRQFESQRIPPPQGLETLRR-TSKGTCKDIYLMTVDLVKRNKLLFAKAFHA
PabSP84     TKTINVNAIKTQFATRDFNIPKDVVKLGG-ATNGSCKSIYDKIIWFINDYKEQMKRAYWA
LolD7       AKAFNINGITKQFKERGMNVPKEVQIFGG--RTDGCKAVYDKTVKVFKTQLQNFRNAFYG
LJL13       ENVFKIDVITKQFNERGLEVPAGLDQELGGSTDGTCKAVYDKSMKFFKSHFMDFRNAYYA
Linb-42     TKSFNTDRIAKQFASQGLSVPDEINYLQG--IAKGCKAVYDKSMKFFKSQGMAIRTAYYA
PPTSP28a    KKLLRVDKIEKQFTSRGVAIPKDLKSMEG-ETDGSCKAIYDKTISFFNNNVADLRTAFYG
PPTSP28c    KKSLRVNKIEKQFTSRGVAIPSDLKSMEG-ETDGSCKAIYDKTISFFNNNVADLRTAFYG
PsSP7       DGSIKVDKVKQQFTSRGIEIPGDIDSISG-PTDGSCKAIYDKTIGFFRNNVQNIRIAFYG
PsSP4       DGSIKVDKVKQQFTSRGIEIPGDIDSLSG-PTDGSCKTLYDKTIRFFKNNAQSIRFAFYG
PabSP54     AQAIRVDKVTQQFKDLNLKAPAEISKLGG-PTNGNCATIYGKTMNFLNSRMTEFRIAFYG
ParSP16     SQTIRADRVKQQFKARGLSVPAEISHLEG-STGGSCVTIYKKTRAFLETQMPNYRIAFYG
PpeSP04     TKTIRVDKVKQQFEGRKLPVPAEISKLEG-PTDGDCEKIYRKTRAFLDAQMKNYRIAFYG
PorMSP28    TKTISVDKVRQQFEGRKLPVPAEIGKLEG-PTGGSCEAIYRKTKAFLDTQMANYRIAFYG
PtSP54      TKTIRVDKVKQQFEDRKLPVPSEISQLGG-PTGGSCEKIYKKTKAFLDTQMANYRIAFYG
LayS101     DNSIKIEAIAQQFRSRGLNVPTSLQKVAG-PTSGSCKDVYQKTIKFFLEQKANIQHAYYG
PtSP57      TGSISIGKVREQFSSRNLKVPAEVKKLKG-PTNGSCKEVYDKTIAFFKSQKTSLQKAYYG
PpeSP04B    KHSIKIDKVKNQFSSRGVQIPPELEKIGG-PTSGSCKDVYDKTIAFFKSQKTNLQKAYYG
PtSP58      YGSIKIDKVKIQYSSRGLHIPVGLRKLSE-PTNGFCKDVYDKPIDFFKSQKSNLQKAYYG
PorMSP43    TGSIKIDKVEKQFSSQGVPIPAELEKIGG-KTSGSCKDIYDKTIDFFKSQKSNLQKAYYG
PorMSP38    YGSIKIDKVKIQFSSRGVHIPAGFRKIGE-PTNGFCKDVYDKTIDFFKSQKSNLQKAYYG
ParSP12     TGSIMINTVATQFITRGMKVPAEVNNLSG-STSGSCSDIYKKTIGFFKSQKANIQKAYYG
PabSP20     RKSIKIDTVKKQYKARGVTIPNGISKIGG-PTDGSCTGVYKKTIDFFISQKTNLQKAYYG

                                       *            *
PabSP59     NSGETAKWYNDNRGTVKGKYQKASEFCKSKE------KECQLHCRFYYYRLVDEDYQI-F
PtSP42      ISAETVKWYNENKGSVKAKYQKASEFCKSQS------DECRVHCRFYYYRLVDEDYQI-F
PabSP84     TRQDAGKWYAANKGYVKALNQTASDFCNGFKK----DPDCNLDCRFYYYRWIDEDHLFYK
LolD7       TKESSDEWFKANPN-TKPKGTKISTFCEAKGI-----GDCIHSCSFYYYRLIDEDNLIIP
LJL13       TYDGSDEWFSKNPD-VKPKGTKVSEYCKNKDD-----GDCKHSCSMYYYRLIDEDNLVIP
Linb-42     TNESEEKWFNAHPD-TKPKGPKISTFCKSKGD-----GGCEHSCSFYYFRLIDEDNLIIP
PPTSP28a    TIEESNKWYAQNPD-AKPKGTKISNFCKANNREQGK-NNCKHACSAYYYRLVDEDFEPIY
PPTSP28c    TIEESNKWYSENPD-AKPKGTKISKFCKDNNREQGE-SNCKHACSAYYYRLVDEDFEPIY
PsSP7       TIPESDKWFAEHPE-VKPKGTKISQFCNAE-REKGN-KDCKHACSAYYYRLVDEDYKPIY
PsSP4       TTAESNKWFAEHPE-VKPKGTKISQFCNAE-REKGN-KDCKHACSAYYYRLVDEDYKPIY
PabSP54     TVEGSNKWFSAHPE-TKPKLTKISAFCKG--REGGKEGTCKHACSMYYYRLVDEDNLVIP
ParSP16     TVEESDKWFANNPE-TKPKRIKISDFCKG--REAGTEGTCKHACSMYYYRLVDEDNLVIP
PpeSP04     TYDGSDAWFAEHPE-TKPKNTKISEFCKG--REGGKEGTCKHACSMYYYLLVDEDNLVIP
```

```
PorMSP28  TYDGSDEWFAKHPE-TKPKKTKISDFCKDKNREGGKEGTCKHACSMYYYRLVDEDNLVIP
PtSP54    TYDGSDEWFAKHPE-TKPKKTRISEFCKG--REGGKEGTCKHACSMYYFRLVDEDNLVIP
LayS101   TKAESDKWFAAHPE-TKPKGVKISQFCKGKEK-----SGCKHYCSMYYFRLVDEDNLVVP
PtSP57    TKEESNKWYSENPE-TKPKGAKISVFCKDKNREGGKEGTCKNACSMYYYRLVDEDNLVIP
PpeSP04B  TKEDSNKWYSENPD-TKPKGTKISVFCKDKNREGGKEGTCKHSCSMYYYRLVDEDNLVIP
PtSP58    SKEESDKWYSENPE-TKPKGTKISVFCKDKNREGGKEGTCKHACSMYYFRLVDEDNLVIP
PorMSP43  TKEDSNKWYSENPE-TKPKGTKISVFCKDKNREGGTEGTCKHACSMYYYRLVDEDNLVIP
PorMSP38  TKEDSNKWYSENPE-TKPKGTKISVFCKDKNREGGTEGTCKHACSMYYYRLVDEDNLVIP
ParSP12   TKEESDNWYSKHPN-VKPKGTKISDFCKG--REGGTEGTCKHACSMYYYRLVDEDNLVIP
PabSP20   TIEASNEWYSKNPN-VKPKGTKISDFCKAENREGGKEGTCKHACSMYYYRLVDEDNLVIP

                          *         *        *
PabSP59   NRKFKIYGISDAQLRQCREKASQAKGCRVAKTLKQCLEKIDSEKVKRALKSLDEISATY-
PtSP42    NRNLKINGITNAQLQQCRMKASQAKGCQVAKVLRNCLKEINPEM----------------
PabSP84   DIKLRVDGIPRDKVKKCKREASKERKCKVARVLYECLGKADKDALDRSLKLLDNLSLSTY
LolD7     FSYL--PGYPETKLKECRRQVQDLAGCK-SDALFLCLKNADKAALEASLKQLDDQSKVY-
LJL13     FSNL--PDYPEDKLEECRNEAKSANECK-SSVIYQCLENADKSALDASLNILDEFSGRY-
Linb-42   FSDL--PGYPKSALEECRNQVKSTNGCK-SSELYECLSKADKPALDSALEQLDNWSERY-
PPTSP28a  FRLLEIKGFSNKDIDECIKHASGRQGCQRSDALYDCLINKNSAALKAALQILDDQSARTY
PPTSP28c  FRLLEIKGFSNEDIDECIKQTSGRQGCQSSDALYDCLKNKNSAALKAALQILDDQSARTY
PsSP7     FRKLEIPGISNDKIAECRKEASGQRGCKVSDALYDCLERSNAAGLKAALKILDDQSAKY-
PsSP4     FRKLEIPGISNDKINKCRKEASGQQGCKVSDALYDCLERSNAAGLKAALKILDDQSTKY-
PabSP54   FRKL--SGFKEADLKKCRDKASSKSGCKVADKIYNCLNTINPDGLKEALRTFDEQSATMY
ParSP16   FRKL--PGILDSQLEQCRDQASSETGCKVGDTIYNCLNRINPEGLKKALNTLDEQSLTLY
PpeSP04   FRKL--PGISESDLKQCRDAASKKSGCQVADTIYDCLNKINPTGLKTALNTLDEQSLTNY
PorMSP28  FRKL--PGISESDLKECRDAASKKTGCKVADEIYECLHNVNPKGFEDALKTLDEQSLTNY
PtSP54    FRKL--PGISESDLKECRDAASKKTGCKVADEIYECLHNVNRKGFEDALKTLDETSLTNY
LayS101   FRKL--PGYPEPKLQECRNKARATTGCKVADVLYECLKRDFPTYLSMILQNYDNESEYY-
PtSP57    FRKL--PGISESDLKECRDVASKKTGCEVADKLYECIDKANSKAFRDALKKLDDESAVY-
PpeSP04B  FRKL--PGISESDLKECRDAASKKTGCKVADEIYECLHNVNRKGFEDALKKLDGESAVY-
PtSP58    FRKL--PGISESDLKECRDAASKKTGCKVADEIYECLHNVNPIGFENALKKLDDESAVY-
PorMSP43  FRKL--PGISESDLKECRDAASKKTGCKVADEIYECLHNVNPKGFEDALKKLDDESAVY-
PorMSP38  FRKL--PGISESDLTECRDAASKKTGCKVADEIYECLHNVNPKGFEDALKKLDDESAVY-
ParSP12   FRKLKIPGIPGPKIDECRRKASSKTGCKVADALYKCLKAINGKSFENALKKLDEESSRTY
PabSP20   FRKLNIQGIPGPKIEECRRIASSKSGCKVSDALYSCLNKINSQGFIAALKKLDEESSRSY

PabSP59   ------
PtSP42    ------
PabSP84   KCTSKN
LolD7     ------
LJL13     ------
Linb-42   ------
PPTSP28a  ------
PPTSP28c  ------
PsSP7     ------
PsSP4     ------
PabSP54   ------
ParSP16   ------
PpeSP04   ------
PorMSP28  ------
PtSP54    ------
LayS101   ------
PtSP57    ------
PpeSP04B  ------
PtSP58    ------
PorMSP43  ------
PorMSP38  ------
ParSP12   ------
PabSP20   ------
```

B

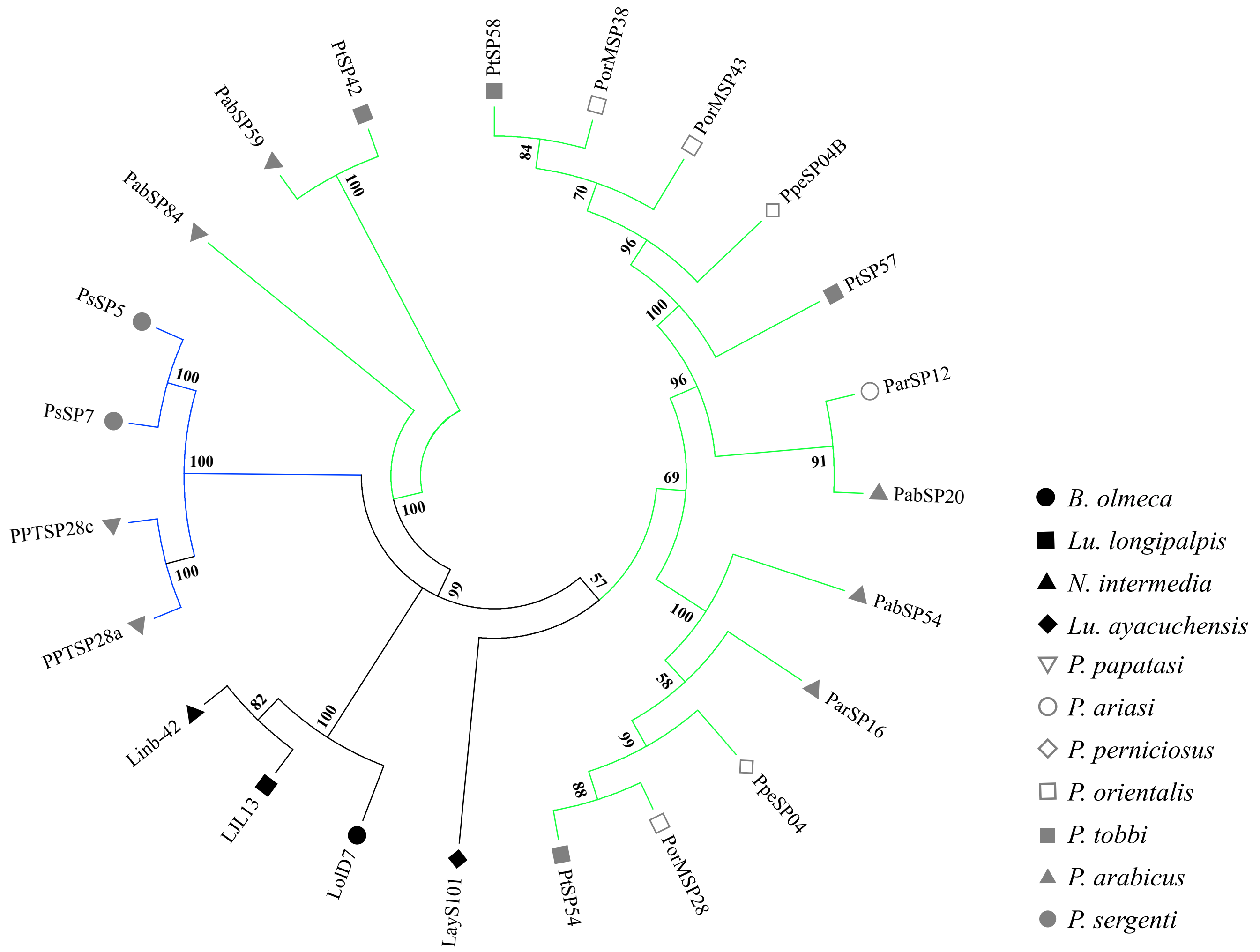
PtSP58
PorMSP38
PorMSP43
PpeSP04B
PtSP57
ParSP12
PabSP20
PabSP54
ParSP16
PpeSP04
PorMSP28
PtSP54
LayS101
LolD7
LJL13
Linb-42
PPTSP28a
PPTSP28c
PsSP7
PsSP5
PabSP84
PabSP59
PtSP42
B. olmeca
Lu. longipalpis
N. intermedia
Lu. ayacuchensis
P. papatasi
P. ariasi
P. perniciosus
P. orientalis
P. tobbi
P. arabicus
P. sergenti
