# Supplementary material for: Molecular Diversity between Salivary Proteins from New World and Old World Sand Flies with Emphasis on Bichromomyia olmeca, the Sand Fly Vector of Leishmania mexicana in Mesoamerica
Source: PLoS Negl Trop Dis. 2016 Jul 13;10(7):e0004771. doi: 10.1371/journal.pntd.0004771 (PMC4943706; doi:10.1371/journal.pntd.0004771)
Supplement: S10 Fig — (A) Multiple sequence alignment of the Antigen 5-like protein (LolAg5) identified from the B. olmeca salivary gland transcriptome with homologs identified from Lu. longipalpis (LuloAg5), Lu. ayacuchensis (LayS79) and N. intermedia (Linb-13), P. ariasi (ParSP05), P. arabicus (PabSP4), P. orientalis (PorASP74), P. perniciosus (PpeSP07), P. argentipes (PagSP05), P. papatasi (PPTSP29), P. duboscqi (PduM48, PduK107). Black background shading represents identical amino acids. Grey background shading represents similar amino acids. (B) The phylogenetic analysis displays New World and Old World sand flies in distinct branches. For the Old World sand flies, Antigen-5 protein belonging to sand flies of closely related sub-genera clustered together. The Whelan And Goldman model [62] was used to infer the evolutionary history of the sand fly Antigen-5 proteins. Sand fly species are indicated by different symbols. Tree branches were color-coded so as to represent specific taxa: Green color represents the Larroussius and Adlerius subgenera; Red color indicates the Euphlebotomus subgenus; Blue color points to proteins of the Phlebotomus and Paraphlebotomus subgenera; and Black color indicates the proteins belonging to New World sand flies. (PDF) [file pntd.0004771.s010.pdf]

A

```
ParSP05     --NDYCEPKLCKFNNQV--KTHIGCKNDGKFVESTCPKPNDAQMIDMTEQRKNLFLKIHN
PabSP4      --NDYCQPKLCTNGQRV--SAHIGCKNDGKFVAGNCP--NDAQMVNMSPERKNLFLKIHN
PorASP74    --NDYCQPKLCTNGKTV--RPHIGCKNNGDFDRSACP--NDAELLEMTQERKNLFLKIHN
PpeSP07     --NDYCQPKLCTNGKTV--KPHIGCRNNGDFDRSACP--NDAQMVEMTQQRKELFLKIHN
LolAg5      -QSNYCAQEPCTSRG----SKHIGCQNSGQFS-PTCP--RDAQIIKINKKNQNLLVKIHN
Linb-13     -QSDYCAQEPCTSRG----KQHIGCRNNGQFV-STCP--RDAQIIKINKKNQNLLVKIHN
LuloAg5     -QSNYCKQESCSSGG-V-ERPHIGCKNSGDFS-ETCS--GDAEIVKMDKKKQNLLVKMHN
LayS79      --QDYCKQESCSSSG-V-AKPHIGCKNNGQFA-QTCP--KDAEIIPMNKKNKNLLVKIHN
PagSP05     -QTNYCQPKLCTFGPGLPARPHIGCKNSGQFDRKTCP--KDAQLMPITEDTKKLFLHIHN
PduK107     QNEKYCDQNLCTSN-NV-VKPHIGCKNDGQFK-KSCP--GDAEVVNLSKKQKNLFLKIHN
PPTSP29     -QTDYCDKELCKSGNGE-VRPHIGCNNGGQLA-GNCP--SDTEIVVLTEKQKNLFLKIHN
PduM48      -QTNYCDQKLCTSGYGD-VKPHIGCNNDGQLT-KNCP--SDAKIVELSEKQKNLFLKIHN

ParSP05     RLRDRLARGSVSNFKSAAKMPMLKWDNELARLAEYNVRTCKFAHDQCRSTKACPYAGQNL
PabSP4      RLRDRFARGSVPNYKSAAKMPMLKWDNELARLAEYNVRTCNFAHDQCRATKACPYAGQNL
PorASP74    RLRDRFARGSVPNFKSAAKMPVLKWDNELAKLAEYNVRTCKFAHDQCRATTACPYAGQNL
PpeSP07     RLRDRFARGSVPNFKSAAKMPMLKWDNELAKLAEYNVRTCKFAHDQCRATTACPYAGQNL
LolAg5a     RLRDRFARGAVKGYQPAAKMPMLKWNDELAKLAEYNVRTCKFAHDQCRATDICQYAGQNL
Linb-13     RLRDRFARGAVSGYQPAAKMPMLKWNDELAKLAEYNVRTCKFAHDQCRATNICQYAGQNL
LuloAg5     RLRDRFARGAVPGFAPAAKMPMLKWNDELAKLAEYNVRTCKFAHDKCRAIDVCPYAGQNL
LayS79      RLRDRFAKGAVPGFQSAAKIPMLKWNDELAKLAEYNVRTCKFAHDKCRATNICPYAGQNL
PagSP05     RLRDRFARGSMSPFQSAAKMPMLKWNDELAKLAEYNVRTCEFKHDQCRSANICPYAGQNL
PduK107     RLRDRLAHGSVTPFQPAAKMPMLKWNDELAKLAEYNVRTCKFAHDQCRATKICRYAGQNL
PPTSP29     RLRDRFARGAVKPFKPAAKMPMLKWNDELAKLAEFNVKTCKFAHDKCRSTEICRYAGQNL
PduM48      RLRNRFAGGKVQPFKSAAKMPMLKWNDELAKLAGYNVKTCKFEHDKCRSTEICRYAGQNL

ParSP05     GQMLSSPDFLDPNYVIKNITREWFLEYKWANQGHTDKYMTGSGKNGKAIGHFTAFIHEKS
PabSP4      GQMLSSPDYLAPEYVIKNITREWFLEYQWANQARTDYFMAGSGKDGKQIGHFTAFVHEKS
PorASP74    GQMLSSPDYLDPGYAIKNITREWFLEYQWANQERTNTYTAGSGKNGKQIGHFTAFVHEKS
PpeSP07     GQMLSSPDYLDPGYAIKNITREWFLEYKWADQQRTNTFTGGPGKDGKQIGHFTAFVHEKS
LolAg5a     AVMYSSPDHREMNYVIKNLTREWFWEYRWAKQSQLDKYVGGPGAGGKQIGHFTAFVHEKS
Linb-13     ATMMSLPEHRDINYTIKNLTREWFWEYRWAKQSQLDKYVGGPGKDGKQIGHFTAFVHEKS
LuloAg5     AQMMSYPTHRDLNYVLKNLTREWF-EYRWAKQSQLDNYVGGPGKDNKQIGHFTAFVHEKT
LayS79      AQMMSSPTHRDLNYVIKNLTREWFWEFRWAQQSHTENYVGGPGAGGKQIGHFTAFIHEKS
PagSP05     GQMTSYPDYLDLNYVIKNITREWFLEYKLASQSHTDMFTTGSGKNGKAIGHFTAFIHEKS
PduK107     GQMQSFPEFLNTNIAIKNITREWFREYKDATQDNTDMFTSGRN-GRKQIGHFTAFIHEKS
PPTSP29     GQMQSYPSFLDINIAIKNITREWFREYKDATQANTNKFTSGSN-KGKQIGHFTAFIHEKS
PduM48      GQMQSYPNFLDINIAIKNITREWFREYKDATQANTDRFTSGNN-RGKQIGHFTAFIHEKS

ParSP05     DKVGCAVAKLTNQQYNMKQYLVACNYCYTNMLKEGIYTTGKPCSQCQGKKCDSVYKNLCD
PabSP4      DKVGCAVAKLTNRQFDMKQYLVACNYCYTNMQKEKIYTTGKPCSQCQSKKCDTTYKSLCD
PorASP74    DKVGCAVAKLTNRQFNMKQYLIACNYCYTNMMNERVYSTGAPCSKCQSKKCDSKYKNLCD
PpeSP07     DKVGCAVAKLTNRQFNMKQYLIACNYCYTNMMNEKIYSTGAPCSKCQSKKCDSKYKNLCD
LolAg5a     DKVGCAIARYTNN-QNFKETLLACNYCYTNMMNEKIYTKGKPCSQCHNKKCGPVYKNLCD
Linb-13     DKVGCAIARFTNS-KNFKETLLACNYCYTNMLNERIYTSGKPCSQCHNKKCGPIYKNLCD
LuloAg5     DKVGCAIARFTNE-HNFKETLLACNYCYTNMMKERIYTQGKPCSQCQSKKCGPVYKNLCD
LayS79      DKVGCALARYTNQ-HNFKETLLACNYCYTNMLKEKIYTKGKPCSECQSKKCGPVYKNLCD
PagSP05     DKVGCAISKFVNK-YKFKEYLVACNYCYTNMMKEKVYTKGQPCSQCQSKKCDSVYKHLCD
PduK107     DKVGCAVAKFTNN-NKFKEYLIACNYCYTNMMKEPIYTKGPPCSQCKNKKCGTVYKNLCP
PPTSP29     DKVGCAVAKFTND-HSFKEYLIACNYCYTNMMNEPIYTKGKPCSQCRKKQCGLVYKNLCP
PduM48      DKVGCAVAKFTNK-NNFKEYLIACNYCYTNMMKEPIYTKGPPCSQCKNKKCGTVYKNLCP

ParSP05     ASEKVDPIPDIFKQSRQQRSRK-------------------
PabSP4      ANEKVDPIPDIFKQRRRT-----------------------
PorASP74    ASEKVEPIPDIFLKKPRT-----------------------
PpeSP07     ASEKVEAIPDIFLKKRRT-----------------------
LolAg5a     ASEPVDPTPDVLKQRRGK-----------------------
Linb-13     ENEPVDPTPDLLKQNRGK-----------------------
LuloAg5     PSEKVDPTPDVLKQWKHGK----------------------
LayS79      PSEKVDPTPDVLKQWKQQG----------------------
PagSP05     ASEVIEPIPDILKNPRNGK----------------------
PduK107     ADEKVDPTPEILKNQPRRG----------------------
PPTSP29     EDEEVDTTPEVLKKHRRGGQ---------------------
PduM48      SDEEVDPTPDVFKNQQSRG----------------------
```

B

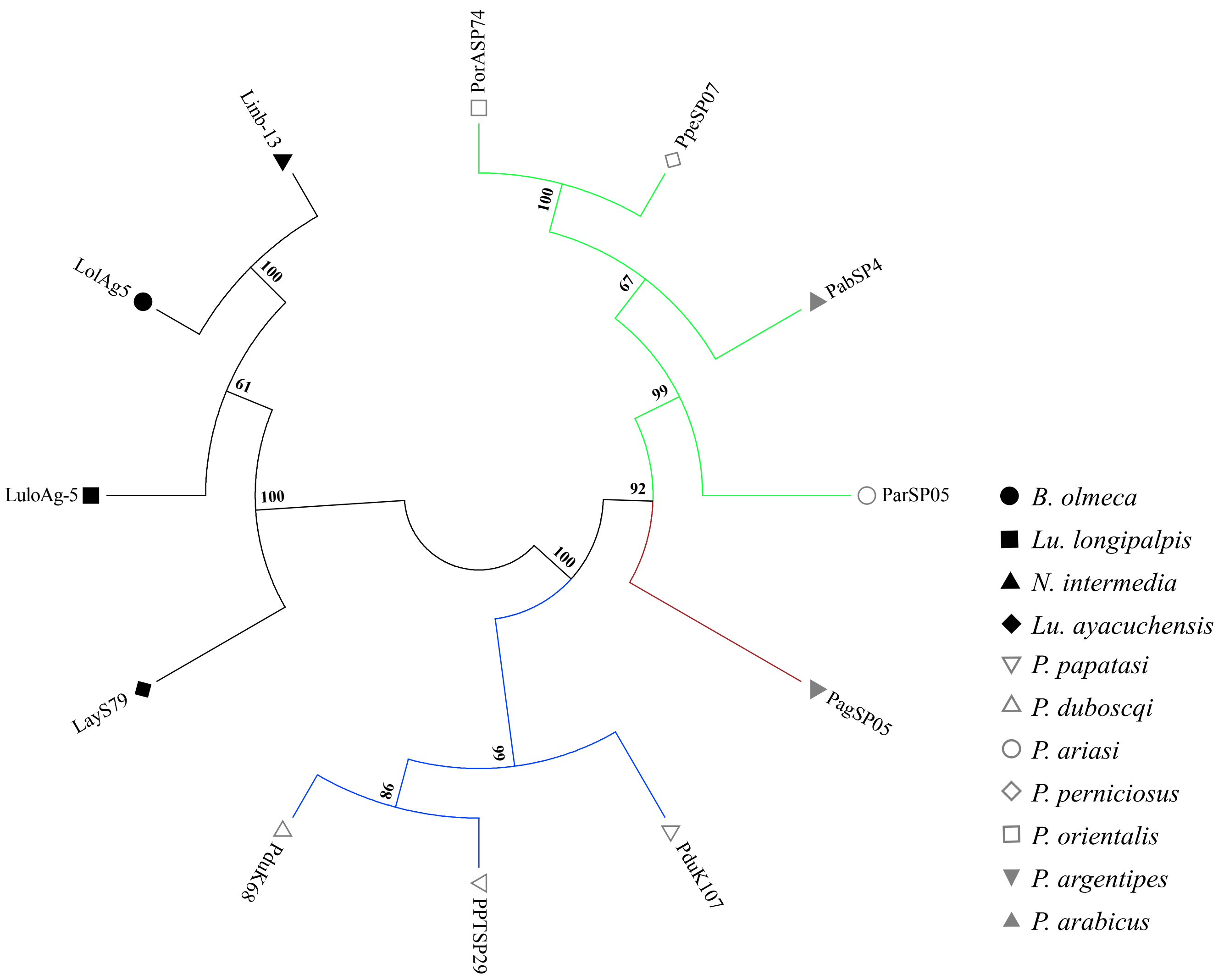
PorASP74
PpeSP07
Linb-13
LolAg5
PabSP4
LuloAg-5
ParSP05
LayS79
PagSP05
PduK68
PPTSP29
PduK107
100
100
67
61
99
100
92
100
99
86
B. olmeca
Lu. longipalpis
N. intermedia
Lu. ayacuchensis
P. papatasi
P. duboscqi
P. ariasi
P. perniciosus
P. orientalis
P. argentipes
P. arabicus
