# Supplementary material for: Molecular Diversity between Salivary Proteins from New World and Old World Sand Flies with Emphasis on Bichromomyia olmeca, the Sand Fly Vector of Leishmania mexicana in Mesoamerica
Source: PLoS Negl Trop Dis. 2016 Jul 13;10(7):e0004771. doi: 10.1371/journal.pntd.0004771 (PMC4943706; doi:10.1371/journal.pntd.0004771)
Supplement: S11 Fig — (A) Multiple sequence alignment of the Silk-related protein (Lolsilk) identified from the B. olmeca salivary gland transcriptome with homologs identified from Lu. longipalpis (LJL04), Lu. ayacuchensis (LayS89) and N. intermedia (Linb-26), P. argentipes (PagSP06), P. tobi (PtSP29), P. perniciosus (PpeSP05), P. orientalis (PorMSP15, PorASP86), P. ariasi (ParSP02), P. arabicus (PabSP30), P. sergenti (PsSP44), P. duboscqi (PduM33, PduK46), P. papatasi (PPTSP32). Black background shading represents identical amino acids. Grey background shading represents similar amino acids. (PDF) [file pntd.0004771.s011.pdf]

```
PagSP06      ARPAIPINKQGKDFPVPTADEK--VTDDYFDDRFYPDIDDEKIGVRKDNGQGSKGGSASQ
PtSP29       APPAIPIAKQGKDFPVPFVSEEN-NPDDYFDDQYYPDINDGGVGSRAPQGTRKPSNRETN
PpeSP05      ALPAIPIARQGKDFPVPFVSEDN-NPDDYFDDQYYPDINDAGVGSKAPQGSRKPPNRGTI
PorASP86     ALPAIPIAKQGKNFPVPFVSEEN-SPDDYFDDQYYPDINDAGVSSKAPQGTRKPPNRETI
ParSP02      APPAIPIAKQGNDFPVPIVDEK--ETDDFFDDRFYPDIDDERVGARAPVGGKQTSNRGTS
PabSP30      APQVIPIAKQGKDFPVPIVDEK--TTDDFFDDRYYPDIDDERVGPKAPTGTRQKPGSKGP
Linb-26      -AKVIPIKQQGKNFPVPQANPAN--SDDYFDDQFYPDINDEKIGEAPRDNRGKTGGGGTG
Lolsilk      -AKVIPIKKQGQNFPVPIVKPGS--SDDYFDDQYYPDINDETIAEAPKDNKRSSVGP---
LayS89       -ANEIPINQQGKNYPLPIADPDK--SDDYFDDRFYPDINDESIVEAPKDNRGKPGGGSKP
LJL04        -ANEIPINRQGKDYPVPIIDPNKSSSDDYFDDRFYPDIDDEGIAEAPKDNRGKSRGGGAA
PsSP44       -AKTIPISKQGKNFPVPFVDPKE--TDDFFDDQYYPDIDDESITEIVRDNQ------GYQ
PduK46       ----IPIKEQGENFPVPFVSQQN--DDDFFDNAFYPDINDESVNKVVRDNGDKRGDRGSQ
PPTSP32      --STIPIQSQGQDFPVPFVSEQT---DDFYDDKFYPDISDDNINEVVRDNGRKGGDRGSQ
PduM33       --KPIPINNQGKHFPVPFVSQQN--DGDFYDDNYYPDINDESINTAVRDNGGKGDSRGSQ

PagSP06      S---RPPAPDK------------------------SPGKTNVSDKRADPPK--------
PtSP29       P------R--GDQSKG---PVSGGRT---L--PG-------------------------
PpeSP05      P------PPRGDQV------SSGGRT---P--PGRVGQGTSPTKDKRARPQI------NR
PorASP86     P------PPRGDQV------S-GGRT---P--PERVGQGAS------------------
ParSP02      SQSDKVPRPQGS-NRG---PSSQTTDKVPR--PQWPSRGTNSQNDKVPRPQG-SSGQTPP
PabSP30      Q-----PTPGGR-T-P---PGSKGPQATP--------GGRTPPGSKG--PQATPGGRTPP
Linb-26      A------SSGG--R--QGGARPGKGGKRPGQGSRRP----------------G---GTR-
Lolsilk      -----------------SGGKLGKGNKKPGHGSGGP----------------S---VTR-
LayS89       A------AAPGGARLGAGGTTPGRGATTPGGGGTRPSAGGSRQNTGRTRPAAG---GTR-
LJL04        G------AR--EGRLGTNGAKPGQGGTRPGQGGTRPGQGGTRPGQGGTRPGQG---GTR-
PsSP44       S------KPSGDKSRPSATPNSGQRPPGRAET---PPASP----ASS----------ASP
PduK46       S------NVPGGASRPSGAPTSGRRPSQSPKGESRPSGAP----TGDRRPSQYPRGESRP
PPTSP32      S------TPSGKESHPSATQTGGRRPSQSPCGESRPSGSA----TSGRRPSQSPRGESLP
PduM33       S------KPSGKETRPSATQTGGRRQSNPSKGESRPSATP----TGGRRPSKSPGGELPP

PagSP06      -ASPCDRKSGR-----------------------------------KGVRD---------
PtSP29       ----------SVG-----------TKDQKPGPQ----INRNPT----GSGA---------
PpeSP05      NPTGTVGQGGSPG-----------TKDKRARPQ----INRNPT----GSGT---------
PorASP86     -----------TG-----------TNNRRPGAQ----NNRNPT----GSGQ---------
ParSP02      RTPGKVEQSGRTN-----------TKDQIPRPL----TNRNPTKNPTEQAR---------
PabSP30      ---GRGGQGGKPG-----------GKDQRTGPA----TGK--------WGK---------
Linb-26      -----PGQGGIA------------------------------------------------
Lolsilk      -----PGQSGSS------------------------------------------------
LayS89       -----RGQGGTRADQGRQRPGN---------------N------------Q---------
LJL04        -----PGQGRTKPAQGTTRPAQ---------------GTRNPGSVGTKEAQ---------
PsSP44       ------------------------------------------------------------
PduK46       SGRPTSGRGPPQYPIGESRPSGSSTSDRRP-PQSPRGESRPPAIFPSSSGRGSFPLPGGQ
PPTSP32      P----------------------------------------ATL---------------
PduM33       R----------------------------------------TTFPS-SGWGSSQVPLEE

PagSP06      ----------------------RTKN------RELVIDESTARV---RQNSQDRKQNHKQ
PtSP29       ----------------------KPKD------RELVIRDKTPSG---DQGGK----PGRL
PpeSP05      ----------------------KPRD------RELVIRDKPPSG---SQGGK----PGRQ
PorASP86     ----------------------KPKD------RELQIKDKTPSG---SQGGK----PGGQ
ParSP02      ----------------------RPGN------RELLIRDKTPG-------SQ----GGKQ
PabSP30      ----------------------GSQG------KELRIREKTT--------------PVRQ
Linb-26      ------------------------TQ----------GDT--------SSVGQRKPQKGGR
Lolsilk      ---------------------ARPAS----------GGT--------SSVGSRNPNK--R
LayS89       ------------GGTRQGGGASRPAQ----------G----------AAGGRKQGTKGTK
LJL04        ------------DASKQGQGKRRPGQ----------VGGKRPGQANAPNAGTRKQQKGSR
PsSP44       ---------------A-------------------------PPCRNPGQQGRK----QGK
PduK46       LPLPETFPTKGVDSLPSPCGESRPSDTFPDSGRRQWDGSETPHRQNSRQQGRR-------
PPTSP32      -----------------------------------------AGRQNSRQQDRR-------
PduM33       SQPSATFPTKSWDSLPLPGRGSRPSDTLPSSARRPCDGFDTSSRQNSRQPGRQ-------
```

```
PagSP06     NRPVQRNLQSYKDAPATYVFKSYDFRENGRTP-IVKLFETNKAEEVIAKGGRND-EYVLD
PtSP29      GKGSKEDLSRYKNAPAKLIFKSSNINTAGKTPNAVRLFKTKKAKTVIAKGGPND-VYVVE
PpeSP05     VRGPKEDLSRYQNAPAKLIFKSSNINTAGKTPSAVKLFKTKKDKTVVAKGGPND-VYEVE
PorASP86    VRGSKEDLSRYKNAPAKLIFKSSNINTTGKTPNAVKLFKTKKAKTVVAKGGPND-VYEVE
ParSP02     GTGNRQKLSSYKDAQPKLIFKSSQFNTDGQNPYLTRLFKTKKVEEVIAKGSPTD-EYVLE
PabSP30     GRGNRQDLSSYKNAQPKLIFKSSQFSTNGKIPSAVKLFRTKKSEEVITTGSPTD-EFVVE
Linb-26     G-NGQGLKAKYANTPVKSIFKSPHFNDAGITP-TVKYFKTKNKEHIMARGGAND-EFVLE
Lolsilk     G-NGQKLKSKYANTPAKYIFKSPKFNDAGKTP-IVTYFKTKNKQHIVARGSPND-EYVME
LayS89      GANRRSDLSKYKDSPAKYIFKSPSFNEEGKTP-IVNYFKTSKKEYTAAGGGPND-EYVLE
LJL04       G-VGRPDLSRYKDAPAKFVFKSPDFSGEGKTP-TVNYFRTKKKEHIVTRGSPND-EFVLE
PsSP44      KGQKKQDLSRYKNSPAKYIFRTGNIDPGKTPD-DVRLFSTSQPEYVIASGNPYD-DYVVE
PduK46      QDRKQQNLSKYRDSPARYIITTGNVDSGNTPN-EIRIFRTNRAEYEIATGDPYKNNYLVE
PPTSP32     QNKKQPDLSKYKNSPARYIFTTGNVDSGKTPD-EERIFRTNRAEYVLATGGPYD-NYLVE
PduM33      QNRNQPSLSNYRNSPAKYIFTSGYVDSSKKPD-EERLFRTNKKEYTIATGDPYT-NYLVE

PagSP06     ILDGK-PYKLSLK-MDATG----TVTVSNPDRERIVGRLKTYKA-------
PtSP29      LLDGN-FNNMSLR-IQIMDRKSSTAILSNPDRNSIVGRVKTYRGLR-----
PpeSP05     LLDEN-FNNMSLR-IQIMDRKSSTAILSNPDRNLIVGRVKTYRGLR-----
PorASP86    LLDGN-FNNMSLR-IQIMDRKSSTAILSNPDRNLIVGRVKTYRGLR-----
ParSP02     LLDGK-PDNLSLV-IRTNGK-TSQAVLRNPTRNRIVGRIKSYNPGPRRMSY
PabSP30     LLDGR-LDNLSLR-IETMGQ-NSKVILRNPNRNRIVGRVKTYKNAYSG---
Linb-26     ILEGD-TSGLRMS-VETVGS-ESRAVLKNPNEKSIVGRVKTYKDGYRRSG-
Lolsilk     ILEGD-PSGIVLS-IQTIGN-ESQVIVKNPNGKPIVGRMKVYKNGYRG---
LayS89      IVDGD-PSGLGLA-VQTIGK-DSRLILKNPKGNNIVGRVKIYRGAYTG---
LJL04       ILDGD-PTGLGLK-SETIGK-DTRLVLENPNGNSIVARVKIYKNGYSG---
PsSP44      IIEGPT-SDLKLKQATIMGR-ESRLILDNPSRGKVIGRVKTYKA-------
PduK46      IIEGPNPNEINLKQITVMGG-DSKIILENPTRPQIVGPY------------
PPTSP32     IIDGPNPNDISLKQSTTMGG-DSKLILDNPNRNTIVGRIKTFKA-------
PduM33      IIQGPDPNDIGLKQLTTMDG-DSRLILENPTGETVVGRVKLTGRERKGN--
```
