# Supplementary material for: Molecular Diversity between Salivary Proteins from New World and Old World Sand Flies with Emphasis on Bichromomyia olmeca, the Sand Fly Vector of Leishmania mexicana in Mesoamerica
Source: PLoS Negl Trop Dis. 2016 Jul 13;10(7):e0004771. doi: 10.1371/journal.pntd.0004771 (PMC4943706; doi:10.1371/journal.pntd.0004771)
Supplement: S13 Fig — Multiple sequence alignment of yellow-related proteins from B. olmeca (LolYLWa-c) Black background shading represents identical amino acids. Grey background shading represents similar amino acids. * Indicates essential amino acids for binding biogenic amines. (PDF) [file pntd.0004771.s013.pdf]

```
                                       *
LolYLWc   AYVEIEYSWSNITYEGLDIKSYKPRYNVPTAFAYDAVNHKLFLTIPRRLPYVPYTVAELD
LolYLWa   ADVSKGYMWNHISLEDIDKGAYDPSHILPTAFAHDANDHTMYLAIPRKVSDIPYTLAEFD
LolYLWb   VDINEGYIWKQILYNDVKPGTYNPDGNIPTAFAHDAISHTLFLTFPRKFPNIPYTIAEVD

                                        **
LolYLWc   TIRHPGFPVERAPQLSKFSGKS-KKDFVPVYQPVIDECRRLWILDVGAIEYNGDDANKYP
LolYLWa   TTKNPGVEGNQEPLVHRFSGHKTGKELTSIYQPVIDECRRMWIVDVGVVEYTED-PKVHP
LolYLWb   TARYPGLKGKQGPLLHKFSGHRTGNELTSVYQPVIDDCRRLWVVDVGSVEYRSKGAKNFP

                                                                    *
LolYLWc   KQKPALIVHDLTKNNNPEIARYEIPKNVAAKPTAFGGFAVDVNKKNGDCSQTFVYITNFE
LolYLWa   IRNPSIVAYDLKTPGRPEVVRYDFPDISAEKPSFFGGFTVDVVNPSGDCSNTFVYITNFD
LolYLWb   SHRPAIVAHDLSKQGHPEIIRYHFPVRLVEKPTYFGGFAVDVV--SRDCSETYVYVTNFL

                                                     *
LolYLWc   ENSLVVFNQKTKTSWKFTDKTFRPDKESTFSHLGDQTTYKVGIFGITLGDRDKKGDRPAY
LolYLWa   TNALIIYDQKNNQFWKVSDPSFAPDVKSTFKHAGKEYKYEFGLFAITLGDRDNEGNRPAY
LolYLWb   SNALFIYDHKNRQSWNITHPTFRGERLSEFNHAGKEYTYNAGIFGITLGDRDAYGNRPAY

                                                *
LolYLWc   YIAGSSTKVYSINTKQLKTKGTKLKPKLHGDRGKHTDAIALAYDAKHRVIFFAESDTRQV
LolYLWa   YLAGSSHKVYSVNTNQLKQKGATLSPVLLGERGEHSDAIALVYDSNTKVIFFAESNTGKI
LolYLWb   YIAGSSIKVYSVNTRELKRQGSTLNPELLGNRGKRSDAIALVYDPKTKVIFFAESNTRQV

                                   * * *            * *
LolYLWc   SCWHI-DMPLKPENTDVIYSYARFIFGTDISIDSENNLWFLSNGYPPIENAEKLKFYERK
LolYLWa   TCWNSQKKPLKPENTHAIFNNEDFIFMTDISVDSKGTLWFMANGMPPIDNSHNFQYEKPR
LolYLWb   SCWNTQKMPLRMQHTDVIYSSAKFIFGTDISVDSKGTLWFMSNGFPPVDNSHKFKFQSSR

LolYLWc   IRLMRVDTDIVLKHAKCNLNYKKPQEIPV
LolYLWa   FRVMQVDTKTAIAGTNCE-----------
LolYLWb   YRLTKVDTKAAIAGTNCEIKP--------
```
