# Supplementary material for: Molecular Diversity between Salivary Proteins from New World and Old World Sand Flies with Emphasis on Bichromomyia olmeca, the Sand Fly Vector of Leishmania mexicana in Mesoamerica
Source: PLoS Negl Trop Dis. 2016 Jul 13;10(7):e0004771. doi: 10.1371/journal.pntd.0004771 (PMC4943706; doi:10.1371/journal.pntd.0004771)
Supplement: S14 Fig — (A) Multiple sequence alignment of the Apyrase (LolApy) identified from the B. olmeca salivary gland transcriptome with homologs identified from Lu. longipalpis (LJL23), Lu. ayacuchensis (LayS17) and N. intermedia (Linb-35) New World species and Psergenti (PsSP42), P. papatasi (PPTSP36), P. duboscqi (PduM38 and 39), P. argentipes (PagSP03), P. orientalis (PorMSP3,4 and PorASP15), P. perniciosus (PpeSP01 and 01B), P. tobbi (PtSP4), P. ariasi (ParSP01) and P. arabicus (PabSP40) Old World species. Black background shading represents identical amino acids. Grey background shading represents similar amino acids. (B) Le_Gascuel_2008 model [64] was used to infer the evolutionary history of the sand fly Apyrase protein family. The phylogenetic tree depicted New World and Old World sand fly Apyrases in distinct branches. For the Old World sand flies Apyrases, sequences from phylogenetically close sand flies clustered together. Larroussius and Adlerius sequences are out-grouped by a Euphlebotomus one, which is out-grouped by Phlebotomus and Paraphlebotomus sequences. Sand fly species are indicated by different symbols. Tree branches were color-coded so as to represent specific taxa: Green color represents the Larroussius and Adlerius subgenera; Red color indicates the Euphlebotomus subgenus; Blue color points to proteins of the Phlebotomus and Paraphlebotomus subgenera; and Black color indicates the proteins belonging to New World sand flies. (PDF) [file pntd.0004771.s014.pdf]

A

```
LayS17    APPGVEWYHFGLIADMDKKSISPSDRNTFNSILKIDELRHNTRTGRYNFVISR-VKKPVSTRFGYKGRGAELSEILIFNKQLYTFDDKTGIVFRMTKDGKLIPWVVLANGDGKQPDGFK
LJL23     APPGVEWYHFGLIADMDKKSIA-SDKTTFNSVLKIDELRHNTKTDQYIYVRSR-VKKPVSTRYGFKGRGAELSEIVVFNNKLYTVDDKSGITFRITKDGKLFPWVILADADGQRPDGFK
LolApy    APPDVEWYHFGLIADMDKKAISKSDPTTFNSDLKIDELQHNTKTDKYTYVISR-VKKPVTTRFAYKGRGAELSEIVVFKNRLYTFDDKSGITFRLTKDGELHPWVILANGNGDSQEGYK
Linb-35   APPGVEWYHFGMIADMDKKSISKSEKNTFNSDLKIDELRHDVKSDRYSYVMSR-IKKPVTTKYGFNGRGGELSEVVVYNNRLYTFDDKTGITFRMTKDGELHPWVILANGDGNRPDGFK
PsSP42    APQGGKSFNFAIIADLDKKSISKTDANNFKSIVKLGELTQVG--TKYDIVMKNKEDREIFTKYAYRGRGAELSEFLRFNRKLYSFDDKSGIVFQLKDNADLVPWVVLANGDGNQKDGFK
PPTSP36   APRSGTIYNFAIIADLDKKSISPKNDNNYKSIVKVGELIQVG--DKYSVKMKK-EDHEIFTKYAYKGRGAELSEFLIYKWKLYTFDDKSGIIFRLKTNADLIPWVTLANGNGDQTDGFK
PduM39    APSSETIYKFAIIADLDRKSISQKNDNNYKSIVKIGQLNQVG--RKFNFAMEN-KDHEIFTKYAYKGRGAELSEFLVYKWKLYTFDDKSGIVFKLKNNADLVPWVILANGNGDQVDGFK
PduM38    APRSGTTINFAIIADLDKKSISKKNDNNYKSIVKLGELYKVA--DKYSFSMKD-EHHEVFTKYAYKGRGAELSEFLVYKWKLYTFDDKSGIVFKLKNNADLVPWVILANGDGDQVDGFK
PagSP03   APRALRFIPFAVIADLDKDSIKD-AGKQFTSIVKYGELRDNG--ENYDLTMKS-QNLHYFTRFAYNGRGAELSELLNFNSKLFTVDDKTGIVFEVKYGGNLIPWVILANGNSNKQEGMK
PorMSP3   APRPTRFIPFAIISDLHRKAMHD-EKNRFTSIVKYGQLKYNG--EKYTLSIRS-ENLHYFTQDTYKGTGAEMSELIYFNNKLYTLNDETGTIYEVKHGGELIPWVTLKNDDGNDKDGFK
PpeSP01   APRPTRFIPFAIISDLHRKAMHD-EKNRFTSIVKYGQLKYNG--EKYTLSIRS-ENLHYFTKNTYKGTGADMSELIYFNDKLYTLNDETGTIYEVKHGGELIPWITLKNDDGNQKDGFK
PpeSP01B  APRATRFIPFALIADLDRKSIKE-DQKSFTSIVKYGELKDNG--ERYTLSLKS-ENLHYFTRYAYNGRGAELSELLYFNDKLYTIGDKTGIVFEVVHGGDLIPWVILSNGPGNQKDGFK
PtSP4     APRATKFIPFALIADLDKKSIKE-DQKSFTSIVKYGELKHNG--ERYTLSLKS-ENLHYFTRYAYNGRGAELSELLYFNDKLYTIGDKTGIVFEVKHGGDLIPWVILANGPGNQKDGFK
PorASP15  APRATKFIPFALIADFDKKSIKE-DQKSFTSIVKYGELKHNG--ERYTLSLKS-ENLHYFTRYAYNGRGAELSELLYFNDKLYTIGDKTGIVFEVKHGGDLIPWVILSNGPGNQKDGFK
PorMSP4   APRATKFIPFALIADFDKKSIKE-DQKSFTSIVKYGELKHNG--ERYTLSLKS-ENLHYFTRYAYNGRGAELSELLYFNDKLYTIGDKTGIVFEVKHGGDLIPWVILSNGPGNQKDGFK
ParSP01   APRATRFIPFAVISDLDKKSIKS-DQKSFTSIVRYGELKDNG--ERYTLSIKS-ENLHYFTRYAYNGRGAELSELLYFNNKLYTIDDKTGIIFEVKHGGDLIPWVILSNGDGNQKNGFK
PabSP40   APRATRFIPFAIVADLDKKSIKS-DQKSFTSIVKYGELKDNG--ERYTLTMKS-ENLHYFTRYAYKGRGAELSELLYFNNKLYSIDDKTGIIFEVKHGGDLIPWVILSNGDGNQKDGFK

LayS17    AEWATVRNDKIYVGSTGITFKD-EKGNANTQSLWVKEITKDGSVTSHDWSQKYKKIREAMKLP-EQGFVWHEAVIWSQIRKEWIFLPRKCSHLAFTPSSEEASGCNLIITADEKFQNIKV
LJL23     GEWATIKDDTIYVGSTGMLK--------FTSSLWVKKITKDGVVTSHDWTDKYRKILKALNMP--NGFVWHEAVTWSPFRKQWVFMPRKCSRHPFSQELEERTGCNKIVTADENFNDIQV
LolApy    AEWATKKGDAIYVGSTGVVFRD-RSGKLSTKALWIKKISKDGAVTSIDWTDIYQKIRNAAKIP--NGFIWHEAATWSDIFKKWVFMPRKCSKDPLSQDNEETTGCNKIIIADENFNDIQV
Linb-35   AEWATIKSNTIYVGSIGVIFKD-KNGKPSPQSQWIKKISKDGTVTSEDWSAIYQKIRNAMKMP--NGFVWHEAAMWSPLRKEWVFLPRKCSKDPISQENEEKTGCNKIITANENFKNIKV
PsSP42    AEWATAKDGKMYVGSTGISWTD-KSGIPNTSSLWIKEIDKEGRVQNKNWEKYYEAVKKAMNIP--NGFVWHEAVNWSPIKKQWVFLPRKCSDLPYNTETEENIGCNEIIIADAVFKTVKS
PPTSP36   AEWATTKGDKMYVGSTGISFTD-KTGKLNSNSLWIKEIDQDGKVQSLDWKEQYDKIKSAMKIP--NGFIWHEAVNWSKLKNQWVFLPRKCSDRPFDTKTEENIGCNKIIIASENFEIIKS
PduM39    AEWATTKGDKMYVGSTGISWSD-STGKLNSNSLWIKEINQDGKVLSSNWKEYYDKMKSAMNMP--KGFIWHEAVNWSKKKNQWVLLPRKCSELPFDTETEETIGCNKIIIASENFQKINS
PduM38    AEWATTKGDKMYVGSTGISWSD-STGKLNSNSLWIKEIDQDGKVLSLNWKQYYDKMKSVMKIP--NGFIWHEAVNWSKLKNQWVLLPRKCSELPFDTNTEETIGCNKIIIASENFQIVRS
PagSP03   AEWATRKGDKMYVGSTGLMWYNEKTKETNSDSMWVKEISRNGEVKSIDWHKQYEAVKKALGMT--NGFVWHEAVTWSSHKKLWVFLPRKCTAEKYSRQIEETTGCNKIITANEDFTKVNA
PorMSP3   AKWATVKGDKLIVGSAGMAFLDAKTMNIDRDALWVKEISESGHITNKYWDSEYKKVRDAMGLF--SGFVWHEAVNWSPRKNVWVFIPRKCTNEPYTVRLDKNTGCNQIITANEDFSDIKT
PpeSP01   AKWATVKGDKLIVGSAGMAFLDAKTMNIDRDALWVKEISESGHVTNKYWDSQYKKVRDAMGLV--SGFVWHEAVNWSPRKNLWVFMPRKCTNEPYTVRLDKKTGCNQIITANEDFSEIKT
PpeSP01B  AEWATVKGDKLYVGSTGMTFLDKRTGTISTNALWVKVIDHNGEVTSINWENQYKKVKDAMGMS--SGFVWHEAVNWSPRKNLWVFMPRKCSRQPFSAQIEEHTGCNQIITANENFNDVRV
PtSP4     AEWATVKDDKLYVGSTGMTFLDKRTGNISKNALWVKEIDKNGEVISINWENQYKKVKDAMGIS--SGFVWHEAVNWSPRKNLWVFMPRKCTNQAFSAQIEENTGCNKIITANENFSDVKA
PorASP15  AEWATVKDDKLYVGSTGMTFLDKRTGNISKNALWVKEINKEGEVISINWENQYKKVKDAMRIS--SGFVWHEAVNWSPRKNIWVFMPRKCSKQQFSAQIEENTGCNQIITANENFSDVKA
PorMSP4   AEWATVKDDKLYVGSTGMTFLDKRTGNISKNALWVKEINKEGEVISINWENQYKKVKDAMGIS--SGFVWHEAVNWSPRKNIWVFMPRKCSKQQFSAQIEENTGCNQIITANENFSDVKA
ParSP01   AEWATVKGDKLIVGSTGIPWFEEKTQSLNTYSLWVKEISKEGEVTNINWKSQYSKVKNAMGIPSSVGFVWHEAVNWSPRKNLWVFMPRKCTTEYFTSQVEEKTGCNQIITANEDFTQVKA
PabSP42   AEWATVKGDKLIVGSTGMPWFNDKHQILDSNALWIKEISTEGEVTNINWKSQYSKVKNAMGIPSSVGFVWHEAVNWSPRKNLWVFMPRKCTTEYFTALVEERTGCNQIITANEDFSQVKA

LayS17    IPVRDHPAEIASGFSAFKFIPGTNNEKLLALRTIEQGEKIATYAVVIDMEGNVLMPEPKLYDEKYEGVAFFGGSQKK------------
LJL23     IHIQDQPYNLASGFSSFRFIPGTKNERLLALRTVEQEDQVKTWAVVMDMKGTVLMYEKELYDEKFEGLAFFGGIKKN------------
LolApy    FDIKDTPKHSASGFSAFRFIPGTYNNRILALRTIEQGKTVETSVVVINIRGRVFMNEKKLYDDKYEGLAFFGGVRKNKS----------
Linb-35   IDIKDTPRNPASGFSTFRFIPGTNNGRILALRTVEKDDLIETSAVVIDMSGKVLMPEKKLYNDKYEGLAFFGGVRRNKS----------
PsSP42    IQIDKNPIDSASGFSSFKFLPDTNDQILIALKTVEKGDKTATYITVIDINGKVLMPEKLINNDKYEGIELLKNAKGLFKRYAL------
PPTSP36   IQIKGKSINRAAGFSSFKFLPDSDDQILLALKTIEKDDKTATYITVIDITGRVLMPEMQINSDKYEGIVLLKSTEGFLKRSQ-------
PduM39    IDIKGTPFDPAAGFSSFKFLPDSDDQILIALKTIEKNGKTATYLTVIDITGKVLMSDKIVNKDKFEGIVLLKSTEGFLKRKE-------
PduM38    IRIKGKSIDPAAGFSSFKFLPESDDQILIALKTIEKNGKTATYLTVIDITGRVLMPDKIINEDKFEGIVLLKNTEGFLKRKE-------
PagSP03   VSITDNKNDPASGFSSFKFIPGTNNEHILAIKTIEKDGATATYAKVITLTGKTLLS-KKILDTKNEGVEFMRNPQGIV-----------
PorMSP3   IKIEGDIKDQASGFSSFKFIPGTKNNDIFALKTIEKNGKIATYGTVIDINGKTLMPERRIIDDKYEGLVFFRHPIGIK-----------
PpeSP01   IKIEGDIKDPASGFSSFKFIPGTKNNDIFALKTIEKNGKIAAYGTVIDINGKTLMPERRILDDKYEGLVFFKHPAGIKKLEHHHHHHHH
PpeSP01B  IHINRAAADSASGFSSFKFIPNTRNNDIFAIKTIERNGQTATYGTVIDINGKTLLPDQRILDDKYEGIAFFKDPKGIKLEHHHHHHHH-
PtSP4     IRIDRDAVDRASGFSSFKFIPNTRNNDIFAIKTIERDGKTATYGTVIDINGKTLLPDKKILDDKYEGIAFFKDPKGIK-----------
PorASP15  INIDRAAVDPASGFSSFKFIPNTRNNDIFAIKTIERNGQTATYGTVIDINGKTLLPDKRILDDKYEGIAFFKDPKGIK-----------
PorMSP4   INIDRAAVDPASGFSSFKFIPNTRNNDIFAIKTIERNGQTATYGTVIDINGKTLLPDKRILDDNMKELHFSGS-QSIK-----------
ParSP01   IRIDGPVQDQAAGFSSFKFIPGTQNNDIFALKTIERNGQTATYGTVINIEGKTLLNEKRILDDKYEGVAFFKNPEGII-----------
PabSP42   IRINGPVEDSASGFSSFKFIPGTQNNDILALKTIEKNGGTATYATVINIEGKTLLQEKWIINDKYEGVAFFKNPKGII-----------
```

B

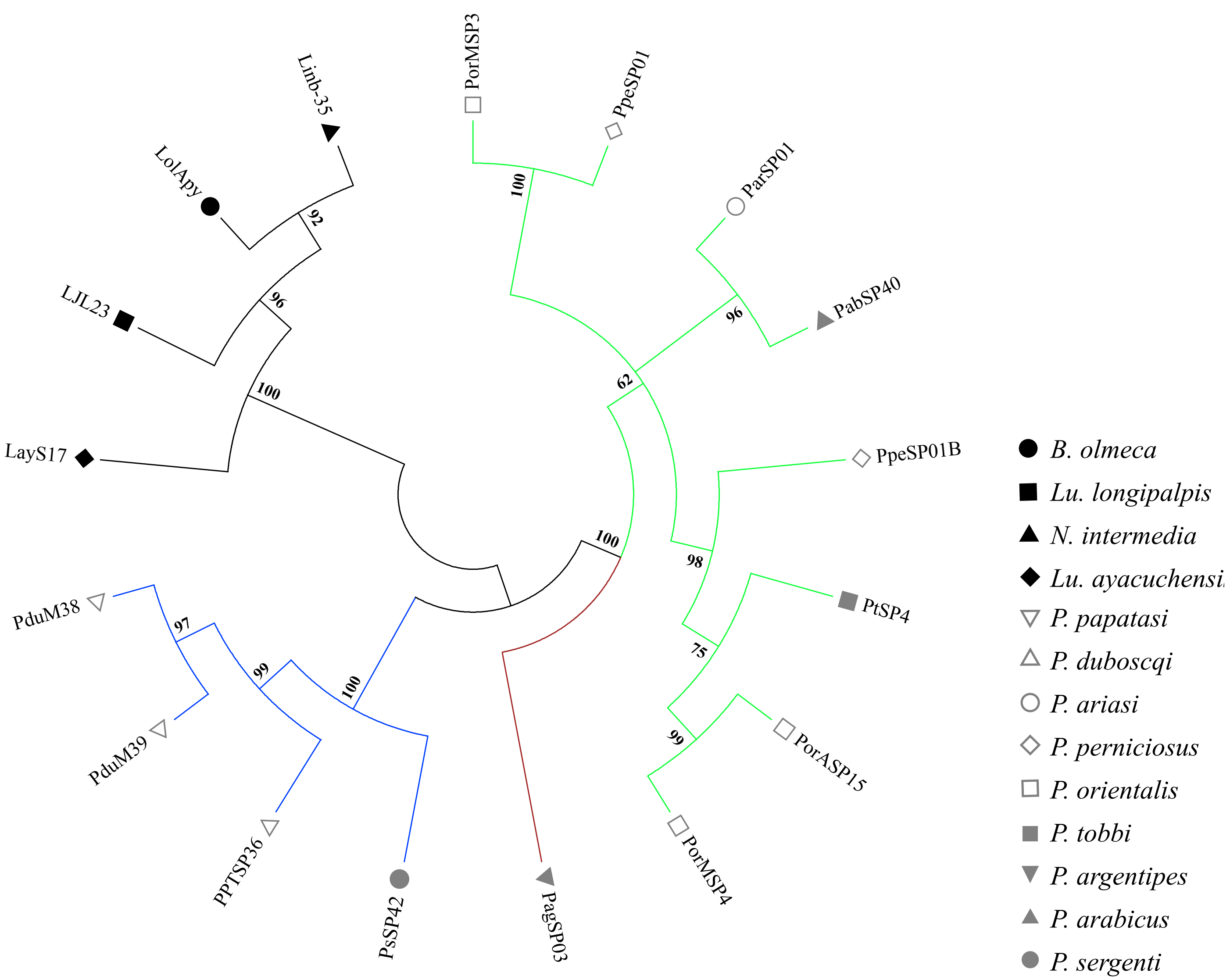

PorMSP3
PpeSP01
ParSP01
PabSP40
PpeSP01B
PtSP4
PorASP15
PorMSP4
PagSP03
PsSP42
PPTSP36
PduM39
PduM38
LayS17
LJL23
LolApy
Linb-35
100
92
96
100
96
62
98
75
99
100
100
97
99
B. olmeca
Lu. longipalpis
N. intermedia
Lu. ayacuchensi.
P. papatasi
P. duboscqi
P. ariasi
P. perniciosus
P. orientalis
P. tobbi
P. argentipes
P. arabicus
P. sergenti
