# Supplementary material for: Molecular Diversity between Salivary Proteins from New World and Old World Sand Flies with Emphasis on Bichromomyia olmeca, the Sand Fly Vector of Leishmania mexicana in Mesoamerica
Source: PLoS Negl Trop Dis. 2016 Jul 13;10(7):e0004771. doi: 10.1371/journal.pntd.0004771 (PMC4943706; doi:10.1371/journal.pntd.0004771)
Supplement: S1 Table — The protein families that bear a scaffold of cysteine residues are listed as well as the number of cysteine residues, the presence or absence in New World and Old World sand flies, and the cysteine residue signatures. (PDF) [file pntd.0004771.s019.pdf]

Supplemental Table 1: Cysteine residues signature of sand fly salivary protein families.

Protein Family	Number of cysteine residues	New World Sand flies	Old World Sand flies	Cysteine Residues Signature
**SALO**	6	Y	N	$CX_{14}CX_{26\text{-}41}CX_{8\text{-}11}CX_{8}CX_{6}C$
**C-Type lectin**	5	Y	N	$CX_{70\text{-}91}CX_{8\text{-}14}CX_{4}CX_{7}C$
**14.2kDa**	4	Y	N	$CX_{11\text{-}13}CX_{18\text{-}21}CX_{12\text{-}14}C$
**ML domain**	6	Y	N	$CX_{13}CX_{5\text{-}10}CX_{42\text{-}47}CX_{5\text{-}11}CX_{39\text{-}43}C$
**Spider toxin**	6	Y	N	$CX_{6}CX_{4\text{-}9}CCX_{4}CX_{5}C$
**Small OBP-like**	5	Y	Y	$CX_{10}CX_{3}CX_{46}CX_{15}CX_{8}$
**D7 protein**	10	Y	Y	$CX_{25\text{-}27}CX_{3}CX_{44\text{-}46}CX_{49\text{-}50}CX_{6\text{-}12}CX_{3}CX_{13\text{-}16}CX_{9}CX_{8}C$
**Antigen-5**	14	Y	Y	$CX_{4}CX_{9\text{-}13}CX_{9\text{-}10}CX_{59}CX_{6}CX_{5}CX_{71}CX_{18}CX_{2}CX_{15}CX_{2}CX_{4}CX_{7}C$
